# Retinal cells derived from patients with DRAM2-dependent CORD21 dystrophy exhibit key lysosomal enzyme deficiency and lysosomal content accumulation

**DOI:** 10.1016/j.stemcr.2024.06.002

**Published:** 2024-07-03

**Authors:** Rozaliya Tsikandelova, Eldo Galo, Edvinas Cerniauskas, Dean Hallam, Maria Georgiou, Rodrigo Cerna-Chavez, Robert Atkinson, Pavel Palmowski, Florence Burté, Tracey Davies, David H. Steel, Martin McKibbin, Jacquelyn Bond, Jennifer Haggarty, Phil Whitfield, Viktor Korolchuk, Lyle Armstrong, Chunbo Yang, Birthe Dorgau, Marzena Kurzawa-Akanbi, Majlinda Lako

**Affiliations:** 1 Biosciences Institute, Newcastle University, Newcastle, UK; 2 Electron Microscopy Research Services, Newcastle University, Newcastle, UK; 3 Leeds Teaching Hospitals NHS Trust, Leeds UK and Leeds Institute for Medical Research, St. James’s University Hospital, University of Leeds, Leeds, UK; 4 Shared Research Facilities, College of Medical, Veterinary and Life Sciences, University of Glasgow, Glasgow G12 8QQ, UK; 5 Glasgow Polyomics and Institute of Infection, Immunity and Inflammation, College of Medical, Veterinary and Life Sciences, University of Glasgow, Glasgow, UK

**Keywords:** DRAM2, CORD21, lysosomes, vesicular transport, autophagy, retinal organoids, RPE cells, lipd metabolism

## Abstract

Biallelic mutations in *DRAM2* lead to an autosomal recessive cone-rod dystrophy known as CORD21, which typically presents between the third and sixth decades of life. Although DRAM2 localizes to the lysosomes of photoreceptor and retinal pigment epithelium (RPE) cells, its specific role in retinal degeneration has not been fully elucidated. In this study, we generated and characterized retinal organoids (ROs) and RPE cells from induced pluripotent stem cells (iPSCs) derived from two CORD21 patients. Our investigation revealed that CORD21-ROs and RPE cells exhibit abnormalities in lipid metabolism, defects in autophagic flux, accumulation of aberrant lysosomal content, and reduced lysosomal enzyme activity. We identified potential interactions of DRAM2 with vesicular trafficking proteins, suggesting its involvement in this cellular process. These findings collectively suggest that DRAM2 plays a crucial role in maintaining the integrity of photoreceptors and RPE cells by regulating lysosomal function, autophagy, and potentially vesicular trafficking.

## Highlights

• CORD21-ROs are characterized by impaired autophagic flux
• CORD21-ROs exhibit lipopigment accumulation reminiscent of *CLN1* and *CLN10* deficiency
• CORD21-ROs and RPE present with CTSD, PPT1, and NPC2 depletion
• Our data suggest a putative role for DRAM2 in clathrin-mediated transport

## Graphical abstract

**Figure:**
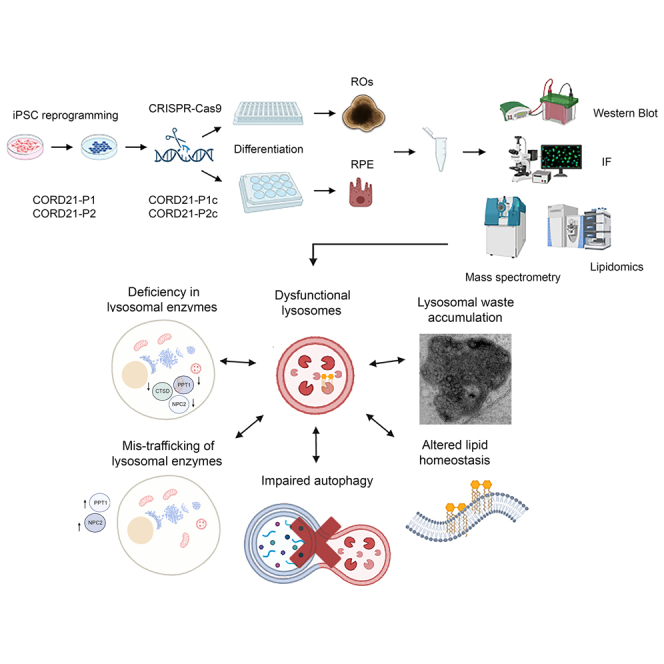

## Teaser

CORD21 autosomal recessive cone-rod dystrophy is associated with biallelic mutations in *DRAM2*. We report that CORD21 patient-derived ROs and RPE cells demonstrate aberrant autophagy, a significant depletion of lysosomal enzymes, and lysosomal contents accumulation, which may underlie CORD21 disease.

## Introduction

Inherited retinal diseases (IRDs) are a primary cause of irreversible vision loss affecting 5.5 million people worldwide (Hanany et al., 2020). Next-generation sequencing has facilitated the identification of IRD-causing variants (Dockery et al., 2021; Neveling et al., 2012; Farrar et al., 2017) resulting in the annotation of a total of 341 IRD-associated genes (RetNet, the Retinal Information Network). Gene identification requires functional studies to prove their causative role and shed light onto pathogenic mechanisms. Validated human patient-derived retinal models are therefore crucial, underscored by the inability of murine models to fully recapitulate human retinal disease due to the lack of a macula (Volland et al., 2015), and the inconvenience of large animal models relating to higher cost, longer lifespan, and time required for disease manifestation (Winkler et al., 2020). The generation of patient induced pluripotent stem cell (iPSC)-derived retinal models, which are more representative of human retinal biology and display molecular features similar to those of the fetal human retina (Foltz and Clegg, 2019; Maeda and Takahashi, 2023), has advanced IRD understanding.

Biallelic mutations in the novel lysosome and autophagy regulatory gene, DNA damage regulated autophagy modulator 2 (*DRAM2*), are associated with an autosomal recessive form of retinal dystrophy known as CORD21 (OMIM **#** 616502), affecting both cones and rods (El-Asrag et al., 2015; Sergouniotis et al., 2015; Abad-Morales et al., 2019; Kuniyoshi et al., 2020; Krašovec et al., 2022). Most CORD21 patients present with macular involvement and loss of cone photoreceptors by the third decade of life. At more advanced stages, the disease presents with macular atrophy, peripheral retinal degeneration, and the inability to see in dim-light conditions (El-Asrag et al., 2015; Sergouniotis et al., 2015; Kuniyoshi et al., 2020; Abad-Morales et al., 2019). A total of 19 unique pathogenic *DRAM2* variants have been reported in fewer than 30 patients (Krašovec et al., 2022); however, the UK Biobank repository has an unpublished record of ∼2,900 individuals with pathogenic or predicted pathogenic variants at the *DRAM2* locus, suggesting greater importance of *DRAM2* than currently recognized.

*DRAM2* encodes a 266-amino-acid lysosomal membrane protein (O’Prey et al., 2009; Park et al., 2009), which facilitates the conversion of endogenous LC3-I (microtubule-associated protein light chain 3 I) to LC3-II (microtubule-associated protein light chain 3 II) (Yoon et al., 2012; Zeng et al., 2014) and the binding of lysosomal proteins LAMP1 and LAMP2 during autophagy (Kim et al., 2017). In the murine retina DRAM2 localizes to photoreceptor inner segments (ISs) and the apical surface of retinal pigment epithelium (RPE) cells (El-Asrag et al., 2015). A recent single-cell RNA sequencing study has shown that *DRAM2* is ubiquitously expressed in the human eye, but the expression is low and comparable between the different retinal cell layers (Jones et al., 2023). Two human *DRAM2* isoforms (a and c) are ubiquitously expressed in all tissues, with the brain and retina displaying increased expression of isoform c compared to other tissues (Abad Morales et al., 2019).

To date, only one published study has investigated the function of DRAM2 in the context of retinal degeneration, showing significant loss of cone photoreceptors in *Dram2* knockout mice from 4 months of age. No difference in photopic or scotopic responses was detected in 21-month-old mice, suggesting that loss of Dram2 in mice leads to age-related degeneration, but the severity of the retinal phenotype does not impair visual function even in relatively old mice (Jones et al., 2023). To gain insights into the pathomechanisms underlining DRAM2-dependent cone-rod dystrophy, we generated iPSCs from two CORD21 patients and differentiated those to retinal organoids (ROs) and RPE cells. Both models showed loss of DRAM2 protein associated with abnormal lipid metabolism, autophagy flux defects, reduced lysosomal enzyme activity, and accumulation of aberrant lysosomal content.

## Results

### DRAM2 expression is significantly reduced in CORD21-ROs and RPE cells

Dermal skin fibroblasts obtained from two CORD21 patients herein named as CORD21-P1 (homozygous for a loss-of-function variant at c.140delG) and CORD21-P2 (compound heterozygote for an intron-exon missense change c.131G>A and a nonsense frameshift variant c.494G>A, Table S1) were reprogrammed into iPSCs (Figure S1A). CORD21 iPSCs were free of Sendai transgenes and displayed the expression of pluripotency markers (Figures S1B and S1C). CRISPR-Cas9 *in situ* gene editing for one mutation (c.140delG in CORD21-P1 iPSCs and c.131G>A in CORD21-P2 iPSCs, Figures S2A and S2B) enabled the generation of heterozygous controls named herein as CORD21-P1c and CORD21-P2c (Figure S1D). CORD21 and corrected iPSCs were pluripotent and lacked genomic instabilities (Figures S1E–S1G) and off-target effects (Figures S2C and S2D).

**Figure 1:**
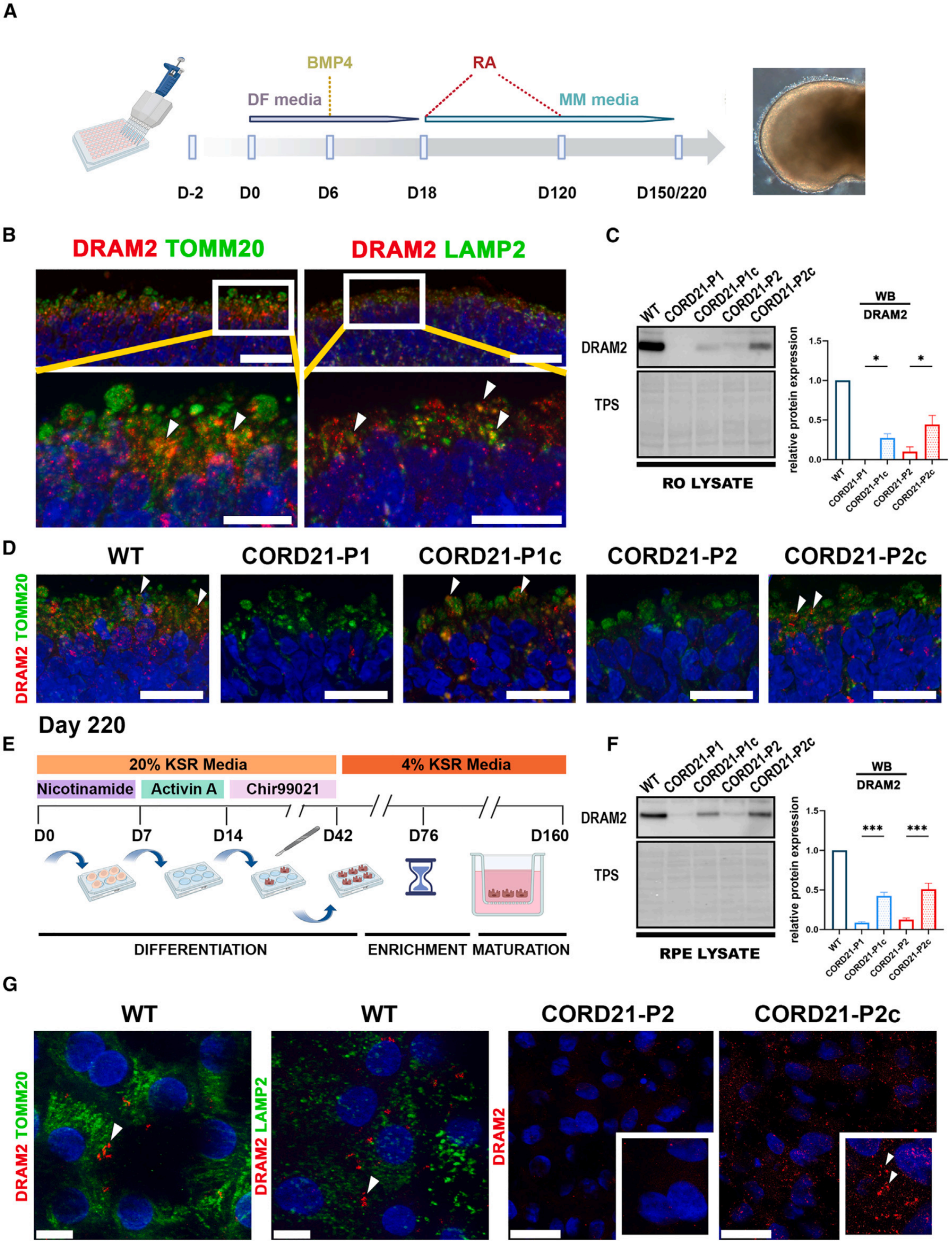
A significant reduction in DRAM2 expression is observed in CORD21-ROs and RPE cells (A) Schematic diagram showing the RO differentiation procedure. DF-differentiation and MM-maintenance media. (B) DRAM2 co-localizes with mitochondrial marker TOMM20 and lysosomal marker LAMP2 in the ISs of wild-type ROs (white arrowheads). These are representative images from 15 ROs imaged from three different differentiation experiments. Scale bars represent 20μm and 10μm in the top and bottom magnified panels, respectively. (C) WB shows a significant reduction in DRAM2 expression in day 220 CORD21-ROs. Data are presented as mean +SEM (*n* = 3 different differentiation experiments each consisting of 48 ROs/sample), ^∗^*p* <0 .05. (D) DRAM2 localizes to the ISs of wild-type and isogenic control (as indicated by white arrowheads) but is absent in CORD21-ROs. Scale bar, 20 μm. These are representative examples from 15 ROs imaged from three different differentiation experiments/sample. (E) Schematic diagram of iPSC-directed differentiation to RPE cells. (F) WB shows a significant reduction of DRAM2 protein abundance in CORD21-RPE cells. Conversely, DRAM2 is detected in the wild-type and the isogenic control ROs. Data are presented as mean +SEM (*n* = 3 different differentiation experiments each consisting of 2 wells of a 12-well plate of RPE cells/sample), ^∗∗∗^*p* <0 .001. (G) Co-localization of DRAM2 with TOMM20 and LAMP2 in RPE cells derived from WT iPSCs and absence of DRAM2 protein in CORD21-P2 RPE cells. A punctate pattern of protein expression can be seen in the CORD21-P2c isogenic control (white arrows). These are representative examples from 15 RPE transwells imaged from three different differentiation experiments. Scale bar, 20 μm.

CORD21 iPSCs and isogenic controls were differentiated to ROs (Figure 1A) and RPE cells (Figure 1E) alongside wild-type iPSCs (referred herein as WT) (Dorgau et al., 2022; Regent et al., 2019). Immunofluorescence (IF) analysis at day 220 of RO differentiation demonstrated the presence of all major retinal cell types in CORD21 and control-derived retinal tissues (Figures S3A–S3E). No changes in the abundance of cells immunostained with markers of all mature retinal cell types including cones and rods were observed between the CORD21- and control ROs (Figure S3F). Qualitative IF characterization indicated the preservation of apico-basal polarity and the presence of tight junction zonula occludens (ZO-1) staining in CORD21 and control RPE cells (Figures S4A and S4B). No changes were observed in the apical and basal secretion of pigment epithelium-derived factor (PEDF) and vascular endothelial growth factor (VEGF), respectively, or ability to phagocytose photoreceptor outer segments (POSs) between CORD21- and control RPE cells (Figure S4C).

To corroborate the specificity of DRAM2 protein detection by western blot (WB), we subjected day 220 WT ROs to *DRAM2*-small interfering RNA (siRNA) knockdown (Figure S5A). Approximately a 50% reduction in DRAM2 expression was confirmed by quantitative reverse-transcription PCR (RT-qPCR) and WB following 72 h of siRNA treatment (Figures S5B and S5C). IF analysis demonstrated punctate DRAM2 localization in the photoreceptor ISs specifically in the mitochondria and lysosomes marked by TOMM20 and LAMP2 immunostaining, respectively (Figure 1B). DRAM2 was localized to the mitochondria and lysosomes in RPE cells (Figure 1G). Complete loss of DRAM2 expression in CORD21-P1 and a significant DRAM2 reduction in CORD21-P2 ROs and RPE cells (Figures 1C and 1F) were observed by WB; however, no DRAM2 expression was noted by IF in CORD21-ROs and RPE cells (Figures 1D and 1G).

### Autophagy impairment and accumulation of lipopigments in CORD21-ROs

**Figure 2:**
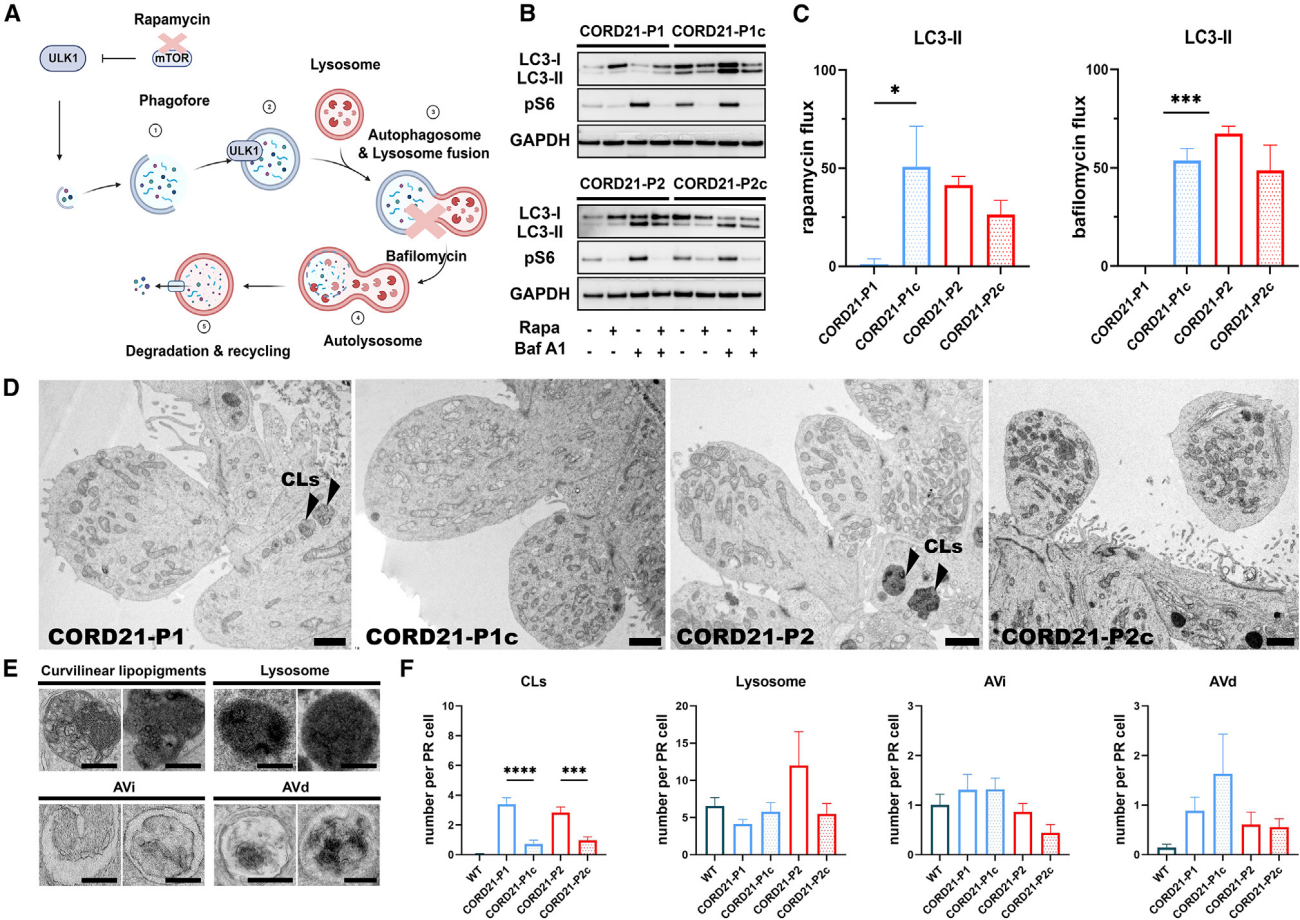
CORD21-ROs present with an autophagy impairment and the accumulation of CLs on TEM (A) Diagram summarizes the effect of rapamycin and bafilomycin drugs on the autophagy pathway. Rapamycin, an mTOR inhibitor, relieves ULK1 from inhibition by mTOR. This leads to the assembly of phagophore double membrane around autophagic cargo and its subsequent maturation to a nascent autophagosome. Bafilomycin blocks the fusion of autophagosomes with lysosomes thereby preventing the formation of autolysosomes. LC3-II is attached to the double membrane of the autophagosome and serves as a marker for autophagosome turnover and degradation. An increase in pS6 expression suggested an inhibition of autophagy following bafilomycin treatment. Schematic was generated using BioRender. (B and C) CORD21-P1 day 150 ROs show reduced rates of autophagic flux compared to isogenic control as apparent by the failure to accumulate LC3-II following single bafilomycin or rapamycin treatment. Data were normalized to GAPDH expression and presented as mean +SEM (*n* = 3 different differentiation experiments each consisting of 48 ROs/sample). (D) CORD21-ROs display increased numbers of CLs (black arrowheads). By contrast no such features were present in the isogenic controls. Scale bar, 1 μm. These are representative examples from 10 ROs imaged from three different differentiation experiments/sample. (E) Magnified images of quantified cellular structures (CL, scale bar, 500 nm; Lysosomes, scale bar, 125 nm; AVi, scale bar, 1.5 μm; 250 nm; AVd, scale bar, 500 nm, 250 nm). (F) Bar charts showing an increase in CLs per photoreceptor in CORD21-ROs, without statistically significant changes in the numbers of lysosomes, early (AVi) and late autophagic vehicles (AVds). Data plotted as mean +SEM, assessed for normality and analyzed by non-parametric Kruskal-Wallis test (*n* = 10 ROs from three different differentiation experiments/sample), ^∗^*p* <0 .05^∗∗∗^*p* <0 .001, ^∗∗∗∗^*p* < 0.0001.

To evaluate the effect of *DRAM2* mutations on the state of autophagy flux in ROs, LC3 expression was evaluated following the sequential addition of 500 nM rapamycin for 24 h and/or 100nM bafilomycin over the course of the last 4 h of the treatment (Figure 2A). Untreated CORD21-P1 and -P2 ROs showed reduced conversion of LC3-I to LC3-II, as demonstrated by the retention of LC3 mainly in the form of LC3-I (Figure 2B). A complete loss of flux in CORD21-P1 ROs was indicated by the failure to accumulate LC3-II following single bafilomycin or rapamycin treatment in contrast to CORD21-P2 ROs, which showed a dramatic increase in LC3-II conversion (Figures 2B and 2C).

Transmission electron microscopy (TEM) revealed a significant accumulation of lysosome-like structures in the photoreceptors of CORD21-ROs (Figures 2D–2F), which were identified as curvilinear lipopigments (CLs) due to their strong resemblance to the granular lipopigments and curvilinear profiles associated with lipid deposition in various forms of neuronal ceroid lipofuscinoses (Anderson et al., 2013). No statistically significant changes in the abundance of lysosomes, the number of early (AVi) and late autophagic vehicles (AVd) (Figures 2E and 2F), and the number of mitochondria or gross alterations in mitochondrial shape between CORD21 and control photoreceptors (Figures S6A and S6B) were observed. The form factor was significantly higher in CORD21-P1 photoreceptors compared to controls, suggesting enhanced mitochondrial branching (Figures S6A and S6B).

### CORD21-ROs show a consistent reduction in CTSD expression

**Figure 3:**
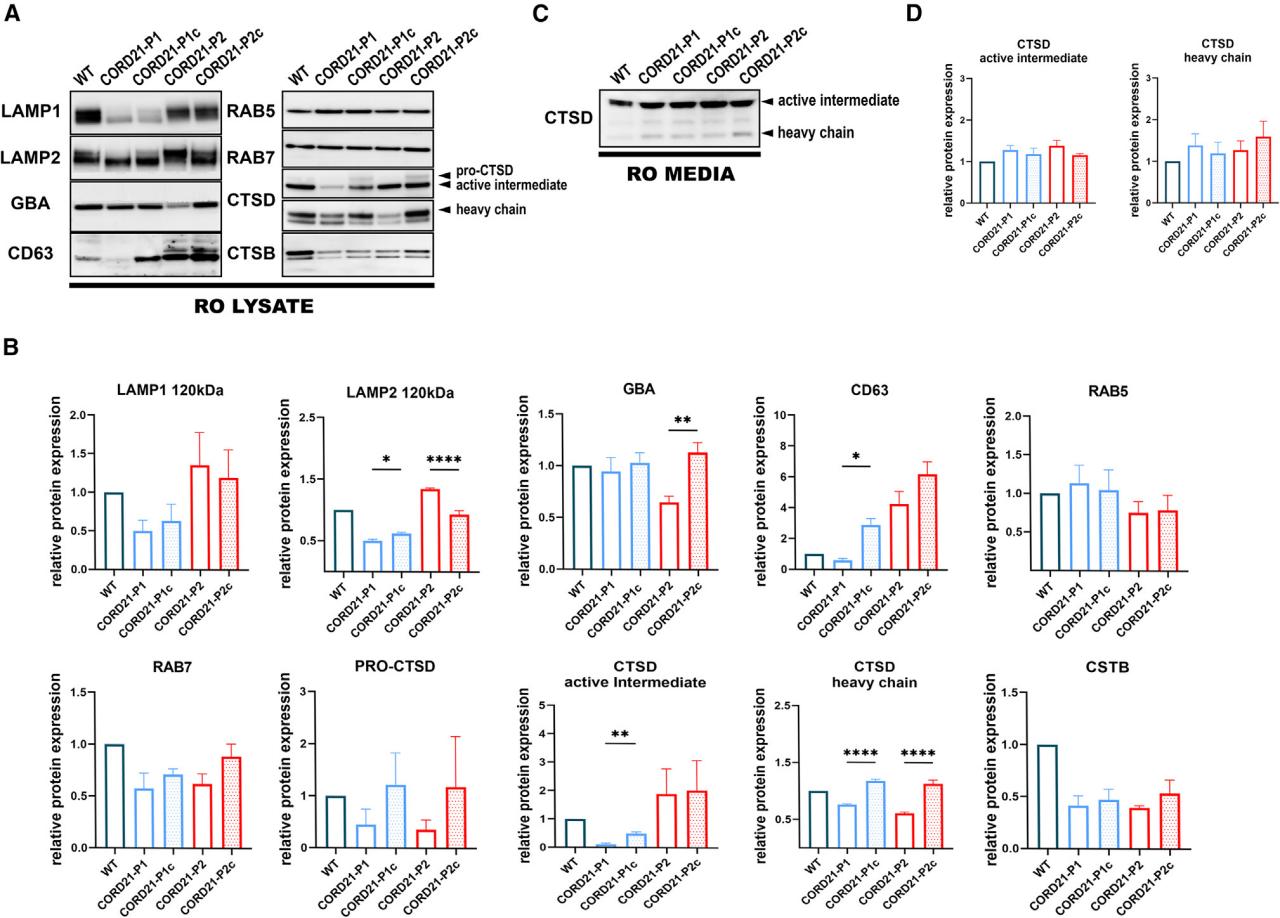
CTSD expression is reduced in day 220 CORD21-ROs lysates (A and B) WB panel followed by quantitative analyses shows a significant CTSD heavy chain downregulation in CORD21-P1 and -P2 ROs. CD63 and GBA protein expression is significantly reduced in CORD21-P1 and -P2 ROs, respectively. (C and D) Secreted CTSD heavy chain and active intermediate show no changes in expression levels between CORD21- and control ROs. (B and D) All data were presented as mean +SEM and normalized to the WT sample (n = 3–5 different differentiation experiments each consisting of 48 ROs/sample), ^∗^*p* <0 .05, ^∗∗^*p* <0 .01, ^∗∗∗∗^*p* < 0.0001.

Extensive WB screening of key lysosomal markers and transport receptors in RO and RPE lysates (Figures 3 and S7) revealed the likely hypoglycosylation of LAMP1 and LAMP2 in CORD21-P1 ROs (Nabi and Dennis, 1998) (Figures 3A and 3B). LAMP1 was likely hypoglycosylated in CORD21-P1 RPE cells, but no changes in LAMP2 expression were observed in either of the patient RPE cells (Figures S7A and S7B). A marked reduction was seen in the protein expression of endosomal sorting marker CD63 and lysosomal enzyme glucocerebrosidase (GBA) in the lysates of CORD21-P1 and CORD21-P2 ROs, respectively (Figures 3A and 3B). No changes in the expression of early and late endosomal markers RAB5 and RAB7 or the lysosomal enzyme cathepsin B (CTSB) were observed between CORD21- and control ROs (Figures 3A and 3B). A key finding was the significant depletion of lysosomal aspartyl protease cathepsin D (CTSD) heavy chain in CORD21-ROs (Figures 3A and 3B) and CORD21-P1 RPE lysates (Figures S7A and S7B). This was not accompanied by enhanced secretion in the media of any CTSD form for either ROs (Figures 3C and 3D) or RPE cells (data not shown). The GBA receptor LIMP2 was downregulated and hypoglycosylated in CORD21-ROs; however, no significant changes in the expression of CI-M6PR (cation-independent mannose 6-phosphate receptor), CD-M6PR (cation-dependent mannose 6-phosphate receptor), or M6PR-independent transport proteins, sortilin and VPS35, were noted (Figures S7C and S7D).

**Figure 4:**
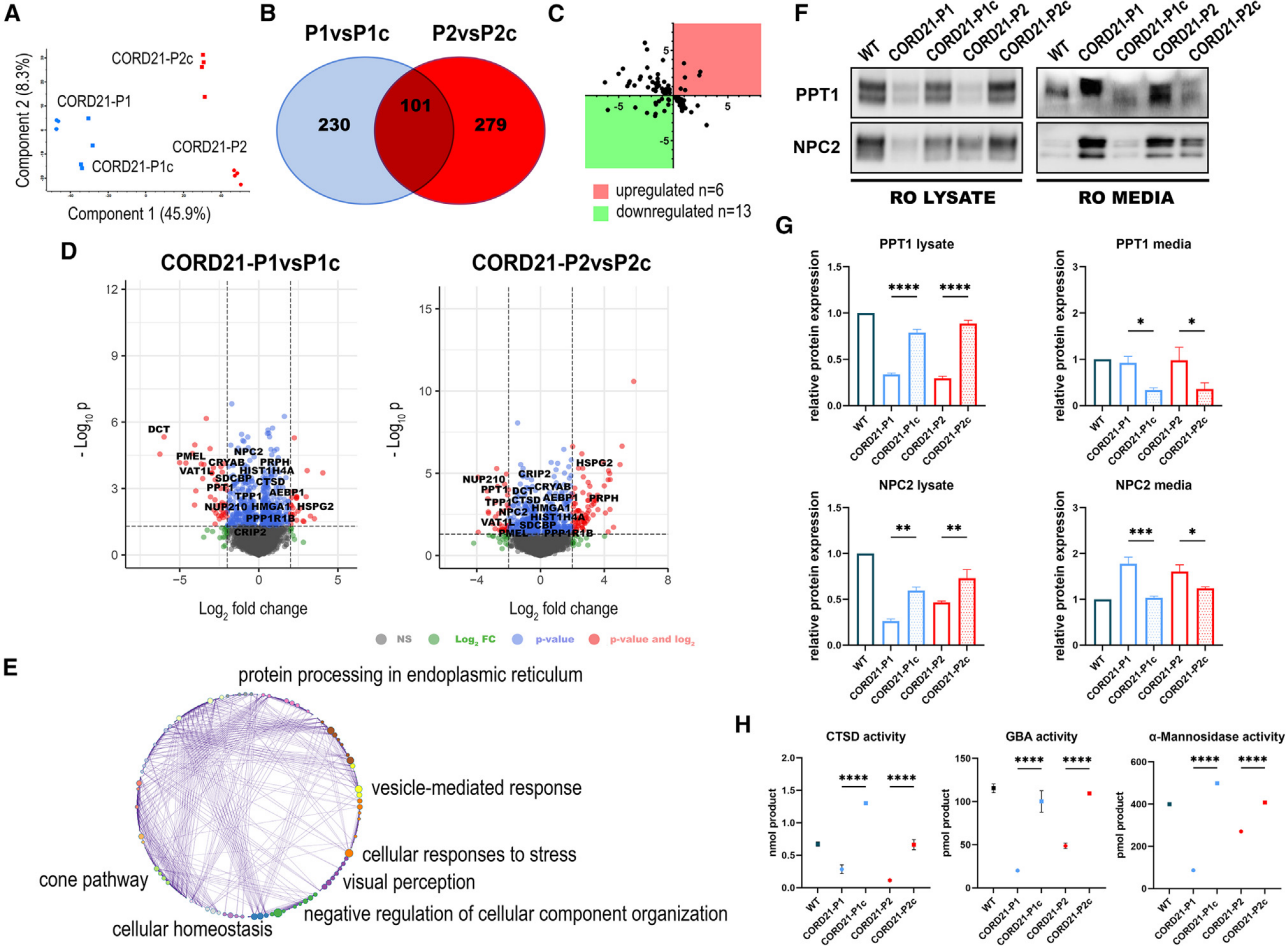
Differential protein analysis identifies vesicle-mediated response as a key biological process in CORD21-ROs (A) Principal-component analysis reveals clear separation between CORD21-P1/P1c (outlined in blue) and CORD21-P2/P2c ROs (red). (B) Venn diagram shows an overlap of 101 commonly changed proteins following Tuckey’s *post hoc* test (false discovery rate [FDR]<0.05) (n = 3–4 different differentiation experiments each consisting of 48 ROs/sample). (C) Dot plot highlighting commonly upregulated (red) and downregulated (green) proteins (*n* = 19). (D) Volcano plots enable visual identification of tandemly changed proteins with logged fold change cutoff >2 that are also statistically significant (logged *p* < 0.05) (*n* = 19). (E) Gene Ontology (GO) term enrichment of significantly changed proteins following the same trend by Metascape revealed major affected biological processes. (F and G) PPT1 and NPC2 enzymes are severely deficient in the lysates of CORD21-P1 and CORD21-P2-ROs relative to controls. This deficiency associates with PPT1 and NPC2 hypersecretion to the extracellular media of matched CORD21-ROs. A total protein stain was used to confirm equal protein loading, and normalization was conducted to the WT sample. Data are presented as mean +SEM, *n* = 3 different differentiation experiments each consisting of 48 ROs/sample. (H) Kinetic assay for the activity of CTSD shows reduced enzymatic activity in CORD21-ROs lysates relative to isogenic control (ANOVA). Endpoint enzymatic activity assays for GBA (ANOVA) and α-Mannosidase (ANOVA) demonstrated a similar reduction in enzymatic activities in day 220 CORD21-ROS. Data were shown as mean +SEM (*n* = 3 different differentiation experiments each consisting of 48 ROs/sample).

**Figure 5:**
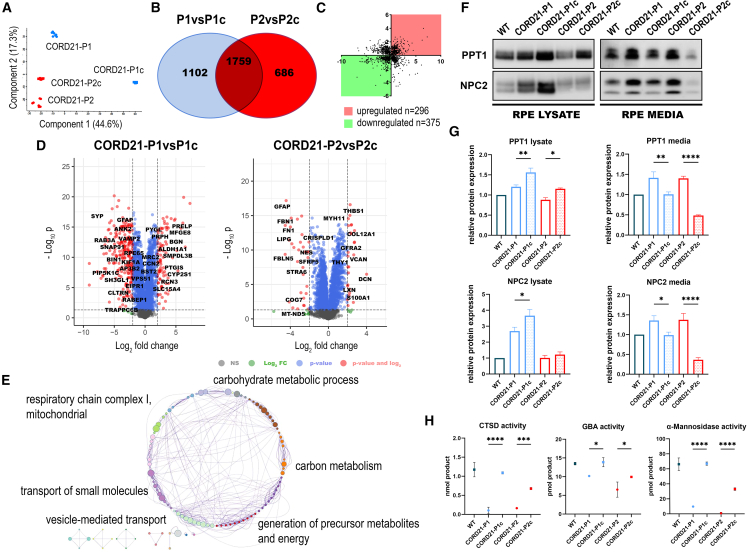
RPE proteome analysis of CORD21-RPE cells identifies changes in key proteins linked to vesicular-mediated transport (A) Principal-component analysis (PCA) showed a distinct separation between CORD21-P1/P1c (blue) and CORD21-P2/P2c RPE cells (red). (B) Venn diagram illustrates the 1,759 proteins that are commonly changed between P1/P1c and P2/P2c RPE cells (Tukey’s *post hoc*, FDR<0.05, *n* = 7 different differentiation experiments each consisting of 2 wells of a 12-well plate of RPE cells/sample). (C) Dot plot revealed a total of 296 (red) and 375 (green) proteins that are up- and downregulated, respectively. (D) Volcano plots show key targets involved in cellular transport in both comparison groups with logged FC > 2 that represent statistically significant changes (*p* < 0.05) (P1/P1c, *n* = 32) (P2/P2c, *n* = 20). (E) GO enrichment analysis of RPE differentially expressed proteins conducted using Metascape identified respiratory chain complex I, carbohydrate metabolic process, carbon metabolism, generation of precursor metabolites and energy, transport of small molecules, and vesicle-mediated transport as affected biological processes. (F and G) WB data showed PPT1 and NPC2 RPE intracellular deficiency is likely due to aberrant secretion to the extracellular media. Equal protein loading was visualized by the total protein stain, and data were normalized to the WT sample. Data are shown as mean +SEM (n = 3–4 different differentiation experiments each consisting of 6 wells of a 12-well plate of RPE cells/sample). (H) Reduced CTSD enzymatic activity in CORD21-RPE lysates relative to isogenic controls. Endpoint activity assays for GBA and α-Mannosidase demonstrated a similar enzymatic reduction in CORD21-RPE cell lysates. Data are shown as mean +SEM (*n* = 3 different differentiation experiments each consisting of 2 wells of a 12-well plate of RPE cells/sample).

We performed mass spectrometry protein analysis of both ROs and RPE cells generated in this study (Figures 4 and 5, Table S2). A total of 4,559 and 3,321 proteins were identified in RPE cells and ROs, respectively (Figures 4B and 5B). Out of the 101 commonly changed proteins in ROs, 19 followed the same tendency for up- or downregulation (Figures 4C–4E, and; Table S2), revealing a common deficiency of CTSD, PPT1, and NPC2 lysosomal proteins in both CORD21-ROs (Table S2). The significant depletion of CTSD and PPT1 was corroborated by RPE proteomics for both patients (Figures 5C–5E, and Table S2). Metascape enrichment analyses in both retinal models outlined vesicle-mediated transport/response as a major affected biological process, whereas mitochondrial respiration (respiratory chain complex I) was shown to be specifically affected in the RPE cells (Figures 4E and 5E).

Following the identification of PPT1 and NPC2 by liquid chromatography-tandem mass spectrometry (LC-MS/MS) as key downregulated proteins, we validated the intracellular PPT1 and NPC2 deficiency (Figures 4F, 4G, 5F, and 5G) and the concomitant accumulation of PPT1 and NPC2 in the media of CORD21-ROs and RPE cells by WB (Figures 4F, 4G, 5F, and 5G). Further, CORD21-ROS and RPE cell lysates were characterized by statistically significantly reduced enzymatic activities for lysosomal degradation enzymes CTSD, GBA, and α-Mannosidase (Figures 4H and 5H).

### Altered membrane-lipid composition and aberrant accumulation of lipids in CORD21-ROs and RPE cells

**Figure 6:**
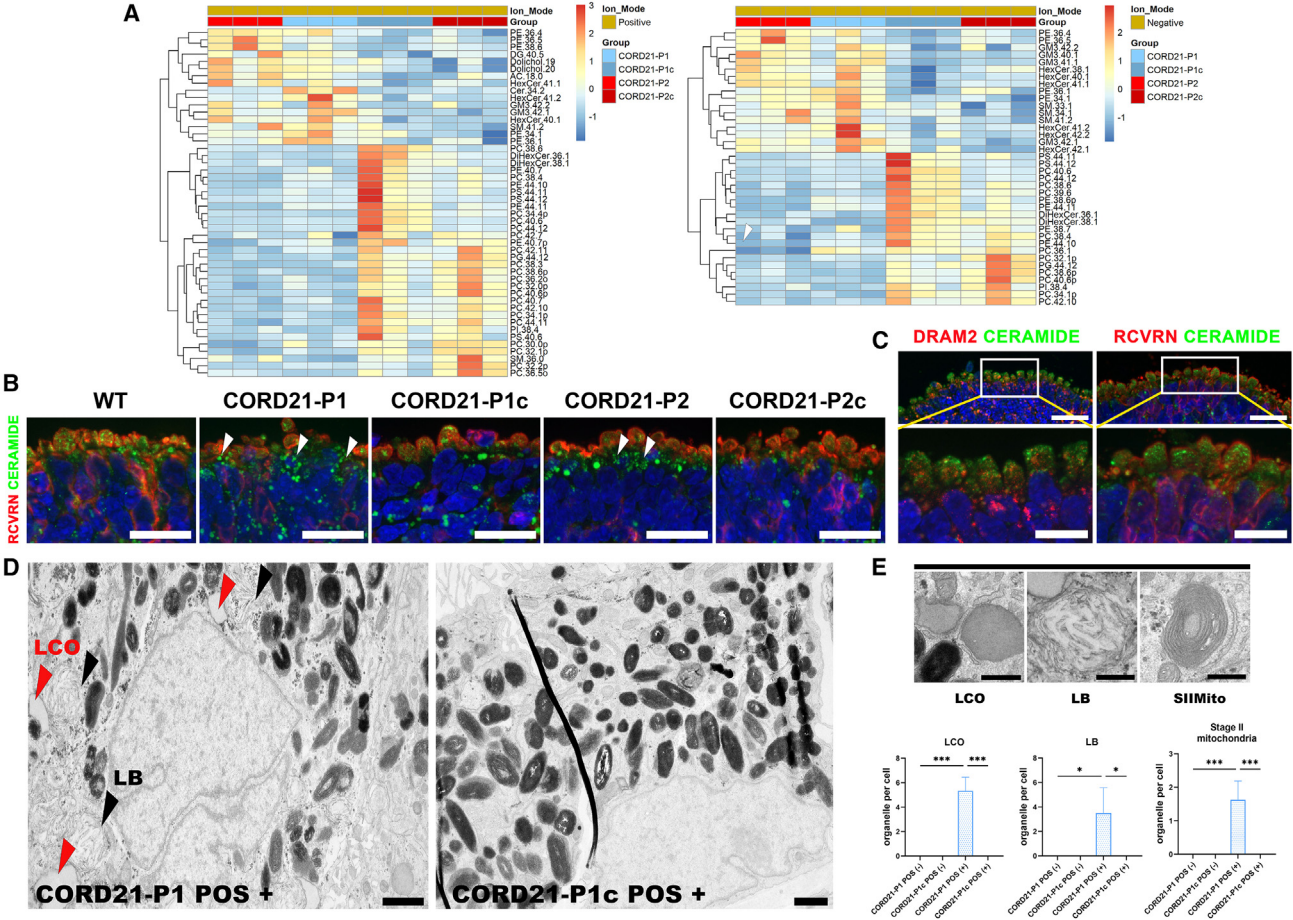
Lipidomic analysis of CORD21 and isogenic control ROs (A) Heatmaps demonstrate statistically significant changes in lipid species under positive and negative ion modes of data acquisition based on t test and *p* value <0.05 cutoff (*n* = 4 different differentiation experiments each consisting of 48 ROs/sample). (B) Recoverin (RCVRN) and ceramide double staining in day 220 ROs shows ceramide accumulation basally to photoreceptor ISs. In WT and isogenic controls, ceramide expression is mostly limited to the IS of the photoreceptor layer indicated by RCVRN staining. By contrast, in CORD21-Ros, ceramide accumulation extends beyond the ISs into the photoreceptor cell bodies, and further basally into the reaches of secondary neurons at the apical edge. These are representative examples from 15 ROs imaged from three different differentiation experiments. Scale bars represent 20 μm. (C) Ceramide expression (green) pertains to the RCVRN+ photoreceptor layer (red, right image) where ceramide is co-expressed with DRAM2 within the IS region of wild-type ROs (red, left image). These are representative examples from 15 ROs imaged from three different differentiation experiments/sample. Scale bars top images 20 μm; bottom magnified images scale bars 10 μm. (D) POS treatment leads to the accumulation of lipid-containing organelles (LCOs, red arrowheads) and lamellar bodies (LBs, black arrowheads) in CORD21-P1 POS (+) relative to untreated CORD21-P1 POS (−) or CORD21-P1c POS (+) RPE cells (scale bar, 1 μm). (E) TEM analysis showed accumulation of LCOs, stage II abnormal mitochondria, and LBs in POS-treated CORD21-P1 RPE cells. LCO (scale bar, 0.5 μm), LBs (scale bar, 0.25 μm). Stage II aberrant mitochondria (SIIMito) (scale bar, 0.5 μm). Plots show the significant accumulation of LCOs, SIIMito, and LBs in CORD21-P1 POS (+) relative to CORD21-P1 POS (−) and CORD21-P1c POS (+) RPE cells (Kruskal-Wallis test). Data are presented as mean +SEM (*n* = 10–12 images of RPE cells from three different differentiation experiments/sample). Statistical comparisons for CORD21-P1 POS (−) vs. CORD21-P1 POS (+), CORD21-P1c POS (−) vs. CORD21-P1c POS (+) and CORD21-P1 POS (−) vs. CORD21-P1c POS (+) are denoted by ^∗^*p* <0 .05, ^∗∗^*p* <0 .01, ^∗∗∗^*p* <0 .001.

To ascertain whether lipid imbalance is associated with the observed abnormalities in crucial lysosomal proteins, CORD21-ROs and isogenic controls were subjected to lipidomics analysis. CORD21-ROs were significantly depleted in phosphatidylethanolamine (PE), phosphatidylserine (PS), phosphatidylcholine (PC), phosphatidylinositol (PI), and phosphatidylglycerol (PG) glycerophospholipids when compared to isogenic controls (Figure 6A). Consistent with a sphingolipid homeostasis defect, CORD21-ROs presented with an increase in monosialodihexosylgangliosides (GM3). A specific finding from the positive ion mode analysis was the accumulation of the isoprenoid alcohol dolichol (Figure 6A). Further, the lipidomic analysis revealed the enrichment of ceramide species in CORD21-ROs (Figure 6A). The buildup of ceramide in CORD21-ROs was shown to occur close to the area of photoreceptor cell bodies as established by IF analysis (Figure 6B) in contrast to control ROs where ceramide was found to co-localize with the IS area of photoreceptor cells, where DRAM2 is also abundant (Figure 6C). Ceramide accumulation was further corroborated in CORD21- RPE cells (Figure S4D).

TEM analysis of CORD21-RPE cells did not show any detectable ultrastructural abnormalities, particularly those associated with lysosomal content accumulation (data not shown); hence we assessed their ability to respond to POS-induced metabolic stress. The POS treatment revealed a variety of aberrant ultrastructural findings in CORD21-P1 POS (+) RPE (Figures 6D and 6E), including the accumulation of lipid-containing organelles (LCOs), lamellar bodies (LBs), and stage II mitochondria in the CORD21-P1 POS (+) sample relative to CORD21-P1 POS (−) and -P1c POS (+) RPE cells. TEM images from the CORD21-P2/P2c comparisons did not show any significant changes, corroborating the more severe phenotype observed in CORD21-P1 ROs and RPE cells throughout this study.

### DRAM2 deficiency in CORD21-ROs and implications for clathrin-mediated transport

**Figure 7:**
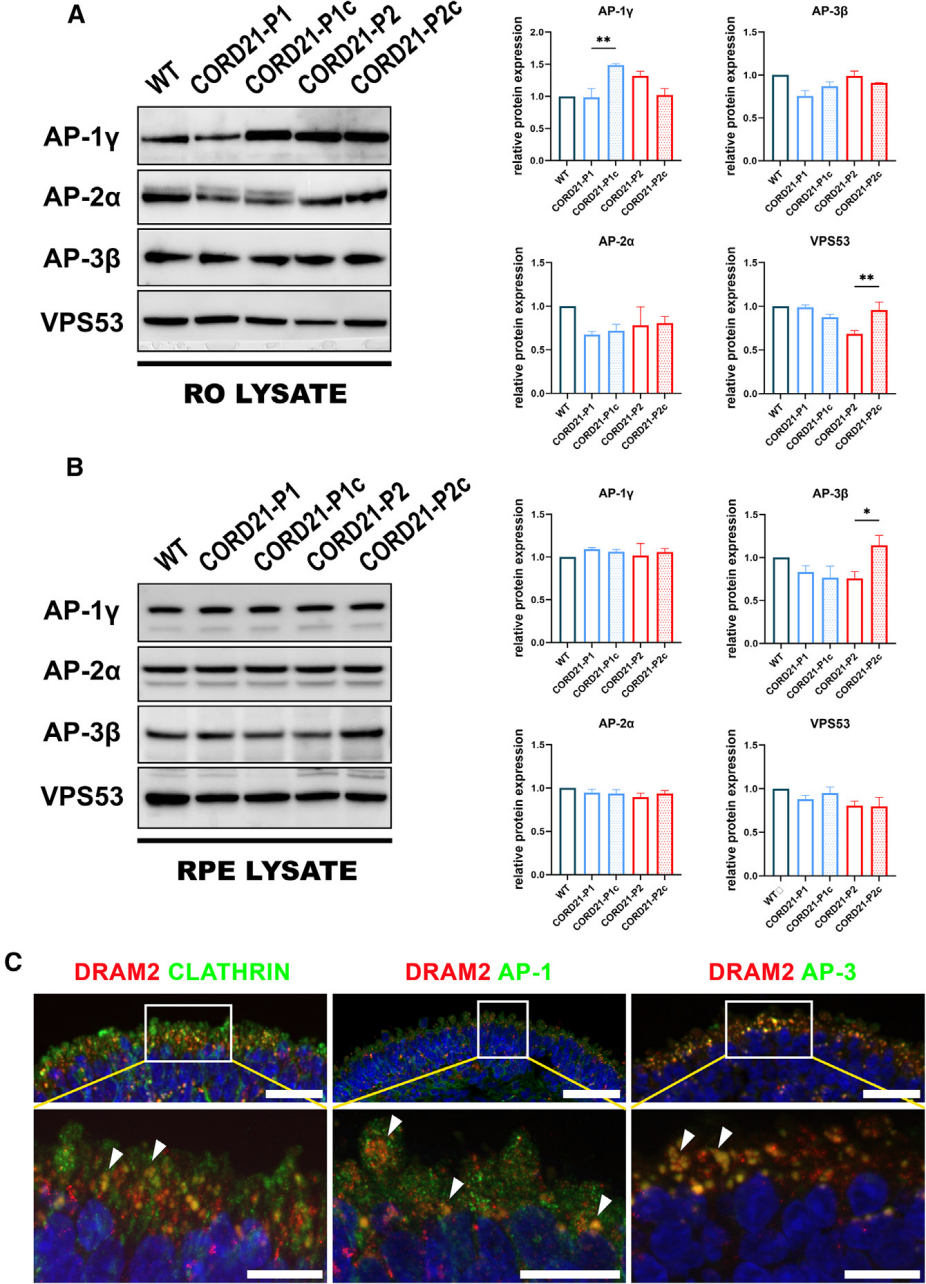
DRAM2 co-localizes with clathrin vesicle adaptors AP-1 and AP-3 and affects their expression (A) A significant reduction in protein expression was established for GARP component VPS53 and AP-1γ adaptor for CORD21-P2 and -P1 RO lysates, respectively. Scale bars represent 20 μm. Data are presented as mean +SEM (n = 3–4 different differentiation experiments each consisting of 48 ROs/sample) and normalized to the WT sample. (B) RPE cell analysis by WB demonstrated a significant downregulation of clathrin transporter AP-3β in the lysates of CORD21-P2 RPE cells relative to isogenic controls. Total protein stain was used to visualize equal protein loading, and data were normalized to the WT sample. Scale bars represent 20 μm.Data are shown as mean +SEM (n = 3–4 different differentiation experiments each consisting of 2 wells of a 12-well plate of RPE cells/sample). Statistical comparisons for CORD21-P1vs -P1c and CORD21-P2vs-P2c are denoted by ^∗^*p* <0 .05, ^∗∗^*p* <0 .01. (C) Clathrin (green) is detected in a dotty-like pattern across the entirety of the photoreceptor IS and can be seen to only partially co-stain with DRAM2 (red, left image). DRAM2 expression (red) strongly overlaps with that of transport vesicle proteins AP-1 (green, middle) and even more so with AP-3 (green, right) at the IS of ROs. Hoechst (blue) counterstains nuclei; white arrowheads show DRAM2 (red) co-localization with clathrin, AP-1, and AP-3 markers (green). These are representative examples from 15 ROs imaged from three different differentiation experiments. Scale bars represent 20 μm. Top panels scale bar, 20 μm; bottom panel scale bar, 10 μm.

Proteomics analyses demonstrated downregulation of multiple subunits of the clathrin adaptors AP-1, AP-2, and AP-3 and vesicle-mediated transport/response as a major affected biological process in both CORD21-ROs and RPE cells (Table S2).Thus to ascertain a putative vesicular trafficking defect, we assessed the expression of major subunits of the clathrin adaptors, AP-1γ, AP-2α, and AP-3β in the lysates of ROs and RPE cells, revealing a significant downregulation of AP-1γ in CORD21-P1 ROs and of AP-3β in CORD21-P2 RPE relative to isogenic controls (Figures 7A and 7B). The GARP (Golgi-associated retrograde protein) component VPS53 was found to be specifically downregulated in CORD21-P2 ROs lysate (Figure 7A). IF experiments in WT ROs demonstrated partial co-localization of DRAM2 with clathrin and a stronger co-staining with AP-1 and AP-3 (Figure 7C).

## Discussion

CORD21 retinopathy is a form of recessive cone-rod dystrophy, typically presenting with a sight impairment, photoreceptor loss, and macular atrophy between the third and the sixth decades of life. A recent study using a *Dram2* knockout mouse model has shown age-related retinal degeneration but no visual dysfunction in relatively old mice (Jones et al., 2023). The difference between the clinical phenotypes of CORD21 cone-rod dystrophy and the *Dram2* knockout mouse model may be due to fundamental differences in retinal structure between mice and humans as well as the short lifespan in mouse, which does not allow modeling of the progressive age-related changes in man (Volland et al., 2015). To gain insights into the pathomechanism of the disease, we generated ROs and RPE cells from two human CORD21 patients, heterozygous isogenic and unaffected iPSC controls. Our data demonstrate a key lysosomal deficiency in CTSD, NPC2, and PPT1 enzymes in CORD21-ROs and RPE cells, associated with reduced lysosomal enzyme activity, impaired autophagic flux, abnormal lipid metabolism, and aberrant lysosomal content accumulation. Based on recent studies on mir144^∗^ in human monocytes, loss of DRAM2 expression in CORD21-ROs and RPE could lead to decreased autophagy (Kim et al., 2017), in line with our data demonstrating the requirement of DRAM2 in the conversion of LC3-II. Together our data suggest a key role for DRAM2 in lysosomal and autophagic function in the retinal cells.

TEM and WB findings for CORD21-P1 ROs and RPE (c.140delG) correlated with a more exacerbated phenotype consistent with an early onset (22 years) and rapid disease progression observed in the CORD21-P1 patient (Table S1) (Sergouniotis et al., 2015; El-Asrag et al., 2015). Conversely, the CORD21-P2 patient (c. 131G>A, c.494 G>A), initially diagnosed at the age of 29, developed central macular atrophy characterized by severely attenuated electroretinograms at the age of 47 (Table S1). The slow-progressing DRAM2 phenotype in CORD21-P2 ROs and RPE is in line with residual DRAM2 protein expression which could be detected by WB (∼30 kDa). Even though c.131G>A is predicted to impact mRNA splicing, it does not affect the retinal-specific *DRAM2c* isoform, underscoring the significance of *DRAM2a* as a disease-causing isoform in the development of CORD21-P1 and -P2 retinal phenotypes (El-Asrag et al., 2015).

We observed the accumulation of aberrant lysosomal structures in CORD21-ROs, a significant depletion of key lysosomal enzymes, namely CTSD, PPT1, and NPC2, and a significant reduction in CTSD activity. CTSD deficiency is associated with mutations in the *CLN10* gene which cause *CLN10* disease, affecting visual and neuromotor development (Williams and Mole, 2012; Schulz et al., 2013). Indeed, DRAM2 deficiency reported here recapitulates *Cln10* mouse phenotypes as inferred by the decreased CTSD activity, the early presentation of lipopigments, and the associated autophagic impairment in retinal cells. The predominant cone phenotype preceding the loss of rods in *Cln10*^−/−^mice (Bassal et al., 2021) is consistent with the classical presentation of CORD21 macular dystrophy (El-Asrag et al., 2015; Sergouniotis et al., 2015; Abad-Morales et al., 2019; Bassal et al., 2021); however, the CORD21-ROs did not reveal changes in the expression of mature cone and rod markers in agreement with the adult onset of CORD21 clinical phenotype (Krašovec et al., 2022).

In contrast to CTSD, NPC2 expression is significantly downregulated in the cytosol, but increased in the media, which could be due to impaired vesicular trafficking. NPC2 is a lysosomal lumen protein which sequesters free cholesterol and removes it from lysosomes (Infante et al., 2008; Kwon et al., 2009). *NPC2* mutations cause a cholesterol and sphingolipid storage disorder known as Niemann-Pick disease type C (NPC), which has severe implications for cognitive, liver, and spleen function (Ribeiro et al., 2001; Park et al., 2003; Vanier and Millat, 2003; Pentchev, 2004; Chang et al., 2005; Evans and Hendriksz, 2017). Disease phenotype arises from the inability of NPC mutants to downregulate cholesterol biosynthesis through the generation of oxysterols (Frolov et al., 2003). As cholesterol is an indispensable component of phospholipid bilayers, its aberrant distribution across cell membranes is likely to perturb the dynamics of cell trafficking (Puri et al., 1999; Radhakrishnan et al., 2008). Our lipidomics analysis conducted on CORD21-ROs and controls did not demonstrate the accumulation of cholesterol or ergosterol esters as outlined by Frohlich et al. (2015) but showed an accumulation of GM3 ganglioside and hexosylceramide species in CORD21-ROs, consistent with the neuronal phenotype of NPC2 deficiency (Vanier, 1999). The lysosomal accumulation of sphingolipids may incur toxicity by inhibiting glycerolipid synthesis (Wu et al., 1993; Contreras et al., 2006) and cause profound defects in lysosomal trafficking (Lloyd-Evans et al., 2008). Indeed, we observed increased levels of ceramide in both CORD21-ROs and RPE cells. Aberrant levels of ceramide have been shown to be toxic to retinal ganglion cells (Fan et al., 2021), photoreceptor (German et al., 2006; Chen et al., 2013), and RPE cells (Levitsky et al., 2020), providing a likely explanation for the retinal toxicity associated with CORD21 retinal dystrophy. Furthermore, an underlying sphingolipid defect is also in agreement with the decrease of GBA enzymatic activity in the lysates of both CORD21 retinal cell models.

An interesting finding from our lipidomics data was the increase of dolichol species in CORD21-ROs. Dolichol, a compound found in ceroid lipofuscin pigments, is implicated in the aging process and various lipid storage disorders (Leloir, 1977). A phosphorylated form of dolichol is involved in N-linked glycosylation of proteins (Rip et al., 1983). Congruent with this, we observed that lysosomal receptors LIMP2, LAMP1, and LAMP2 are likely hypoglycosylated in CORD21-P1 ROs, while no major changes were observed in the glycosylation pattern of CORD21-P2 ROs. Notably, LAMP1 is also likely hypoglycosylated in CORD21-P1 RPE cells (Figure S7A), suggesting that protein glycosylation abnormalities are typical only for CORD21-P1 retinal cells. The complete absence of DRAM2 expression in CORD21-P1 retinal cells, as noted previously, could be the underlying cause. This deficiency may be associated with glycosylation abnormalities occurring upstream in the cellular pathway, possibly within the endoplasmic reticulum (ER) or Golgi apparatus. These abnormalities could disrupt the normal metabolism of lysosomal membranes, which are rich in dolichol. Consequently, this disrupted metabolism could lead to the accumulation of dolichol within the cells.

Similar to NPC2, the cytosolic PPT1 deficiency was associated with extracellular release in CORD21-ROs and RPE cells. PPT1, which exhibits high enzymatic activity in the brain and the retina, is known to facilitate the lysosomal breakdown of lipidated proteins (Chattopadhyay and Pearce, 2000; Dearborn et al., 2016). PPT1 is encoded by *CLN1*, and mutations in this gene are associated with a severe neurodegenerative disorder known as infantile neuronal ceroid lipofuscinosis (INCL, also known as Batten disease) (Santavuori et al., 1973; Weleber, 1998, 2004; Jalanko and Braulke, 2009). Visual deterioration presents as one of the earliest signs of *CLN1* disease and is accompanied by the accumulation of ceroid in the retina (Santavuori, 1988), photoreceptor degeneration, and impairment of second-order neurons and retinal ganglion cells (Weleber et al., 2004). PPT1 has been shown to regulate lysosomal acidification via the lysosomal targeting of the V0a1 vacuolar ATPase (vATPase) (Bagh et al., 2017), and altered lysosomal pH underlies many lysosomal storage disorders (Hu et al., 2015). Hence, the downregulation of PPT1 in both CORD21-ROs and RPE cells could contribute to lysosomal malfunction, leading to the accumulation of CLs.

Comparative proteomic analyses indicated the involvement of DRAM2 in vesicular trafficking, as inferred from the downregulation of multiple AP-1 and AP-3 subunits observed in CORD21-ROs and RPE cells. These findings, combined with DRAM2 deficiency leading to reduced levels of critical lysosomal proteins including CTSD, NPC2, PPT1, MAN2B1 (Mannosidase alpha class 2B member 1), and GBA in ROs and RPE cells, suggest potential vesicular mis-trafficking within the clathrin pathway, resulting in abnormal delivery of lysosomal resident proteins. The identified vesicular pathways implicated in the depletion of these essential lysosomal proteins likely converge on the regulation of lysosomal trafficking by clathrin adaptors AP-1 and AP-3 (Berger et al., 2007), despite discrepancies observed for AP-1 and AP-3 in our WB analyses. Notably, co-localization of DRAM2 with AP-1 and AP-3 in WT ROs further supports a role for DRAM2 in lysosomal function and the regulation of LC3-II conversion. Further experimental work is necessary to definitively establish the regulation of DRAM2 via clathrin-mediated transport, such as co-immunoprecipitation studies with AP-1 and AP-3. Future studies could also benefit from determining the precise subcellular localization of DRAM2 within the endolysosomal and vesicular transport systems.

In summary, our study provides novel insights into the cellular effects of DRAM2 deficiency in photoreceptors and RPE cells, contributing to our understanding of biological mechanisms underlying retinal degeneration.

## Experimental procedures

### Resource availability

#### Lead contact

Further information and requests for resources and reagents should be directed to and will be fulfilled by the lead contact, Majlinda Lako (majlinda.lako@ncl.ac.uk).

#### Materials availability

iPSCs generated in this study are available from the lead contact with a completed Materials Transfer Agreement.

#### Data and code availability

The proteomics data have been submitted into MassIVE:MSV000094178.

### Ethics permission

Dermal skin fibroblasts were isolated from individuals diagnosed with CORD21 retinal dystrophy following acquisition of informed written consent in accordance with the Yorkshire and the Humber Research Ethics Committee (REC ref. no. 15/YH/0365).

### Statistical analyses

Data were assessed for normality using a Shapiro-Wilk’s test. Assuming a normal distribution, the data points across different groups were compared using a parametric one-way ANOVA test (Šídák’s multiple comparisons test). When the data did not meet the normality criteria, a non-parametric Kruskal-Wallis test was applied. Comparisons were performed in pre-selected pairs (CORD21-P1 vs. P1c and CORD21-P2 vs. P2c), whereby the WT control was omitted from the statistical analysis. Statistical analysis was conducted using GraphPad Prism version 9.5.0. All data were presented as mean +SEM values, and statistical significance was assumed when *p* ≤ 0.05 (^∗^*p* ≤ 0.05, ^∗∗^*p* ≤ 0.01, ^∗∗∗^*p* ≤ 0.001, ^∗∗∗∗^*p* ≤ 0.0001).

For details on various assays performed during the course of this study, please refer to the supplemental information.

## Supplemental information

Document S1. Figures S1–S7

Table S1. A summary of CORD21 patient clinical information and the reagents used for CRISPR-Cas9 screening, RT-PCR, western blot, and immunofluorescence analyses

Table S2. A summary of proteomics analyses conducted in CORD21-ROs and RPE cells and the heterozygous isogenic controls

Document S2. Article plus supplemental information
