# Supplementary material for: Retinal cells derived from patients with DRAM2-dependent CORD21 dystrophy exhibit key lysosomal enzyme deficiency and lysosomal content accumulation
Source: Stem Cell Reports. 2024 Jul 3;19(8):1107–21. doi: 10.1016/j.stemcr.2024.06.002 (PMC11368688; doi:10.1016/j.stemcr.2024.06.002)
Supplement: Document S1. Figures S1–S7 [file mmc1.pdf]

Stem Cell Reports, Volume *19*

Supplemental Information

Retinal cells derived from patients with DRAM2-dependent CORD21 dystrophy exhibit key lysosomal enzyme deficiency and lysosomal content accumulation

Rozaliya Tsikandelova, Eldo Galo, Edvinas Cerniauskas, Dean Hallam, Maria Georgiou, Rodrigo Cerna-Chavez, Robert Atkinson, Pavel Palmowski, Florence Burté, Tracey Davies, David H. Steel, Martin McKibbin, Jacquelyn Bond, Jennifer Haggarty, Phil Whitfield, Viktor Korolchuk, Lyle Armstrong, Chunbo Yang, Birthe Dorgau, Marzena Kurzawa-Akanbi, and Majlinda Lako

Retinal cells derived from patients with DRAM2-dependent CORD21 dystrophy exhibit key lysosomal enzyme deficiency and lysosomal content accumulation

Rozaliya Tsikandelova [1], Eldo Galo [1], Edvinas Cerniauskas [1], Dean Hallam [1], Maria Georgiou [1], Rodrigo Cerna-Chavez [1], Robert Atkinson [1], Pavel Palmowski [1], Florence Burté [1], Tracey Davies [2], David H Steel [1], Martin McKibbin [3], Jacquelyn Bond [3], Jennifer Haggarty [4], Phil Whitfield [5], Viktor Korolchuk [1], Lyle Armstrong [1], Chunbo Yang [1], Birthe Dorgau [1], Marzena Kurzawa-Akanbi [1] and Majlinda Lako [1#]

1. Biosciences Institute, Newcastle University, UK
2. Electron Microscopy Research Services, Newcastle University, UK
3. Leeds Teaching Hospitals NHS Trust, Leeds UK and Leeds Institute for Medical Research, St. James's University Hospital, University of Leeds, UK
4. Shared Research Facilities, College of Medical, Veterinary and Life Sciences, University of Glasgow, Glasgow G12 8QQ.
5. Glasgow Polyomics and Institute of Infection, Immunity and Inflammation, College of Medical, Veterinary and Life Sciences, University of Glasgow, Glasgow, UK.

[#] Lead contact and corresponding information:

Majlinda Lako

Biosciences Institute

Newcastle University

United Kingdom

Email: majlinda.lako@ncl.ac.uk

Supplementary Information contains:

Detailed Experimental Procedures

Supplementary Figures 1-8

Supplementary Table Legends

Detailed Experimental Procedures

Cell culture

Dermal skin fibroblast culture

Dermal skin fibroblasts were isolated from individuals diagnosed with CORD21 dystrophy following acquisition of informed written consent in accordance with the Yorkshire and the Humber Research Ethics Committee (REC ref. no. 15/YH/0365). Dermal fibroblasts were maintained in advanced Dulbecco's modified Eagle Medium (Thermo Fisher Scientific) supplemented with 10% FBS (Thermo Fisher Scientific), 1% Glutamax (Thermo Fisher Scientific) and 1% pen/strep (Thermo Fisher Scientific).

iPSC reprogramming

Dermal fibroblasts were transduced at a density of 30,000 cells/cm^2 using the CytoTune™-iPS 2.0 Reprogramming Kit (Life Technologies, A16517) following the manufacturer's instructions. The established patient iPSCs were assessed for pluripotency and clearance of Sendai virus-associated transgenes using primers shown in **Table S1**. The *DRAM2* gene mutations were corroborated by PCR using primer pairs shown in **Table S1**, followed by Sanger sequencing. In addition, iPSCs derived from dermal fibroblasts from two age-matched individuals (WT1 and WT3, collectively named WT) with no history of retinal disease were used as healthy controls as outlined in Buskin et al. 2018. Further information about the cell lines is available in **Table S1.**

iPSC culture

iPSCs were maintained at standard cell culture incubator conditions at 95% humidity, 5% C02 and 37°C. CORD21- and WT-iPSCs were grown and expanded on Matrigel™ Growth Factor Reduced Basement Membrane Matrix (Corning) using mTeSR™ Plus cell culture media (STEMCELL Technologies) supplemented with 1% penicillin-streptomycin (Thermo Fisher Scientific). Cells were passaged in a 1: 6 ratio every 4-5 days using 0.02% Versene- EDTA (Lonza) for 3–5 minutes at 37 °C. All iPSCs as well as differentiated retinal cultures were routinely tested for the presence of mycoplasma using the MycoAlert® Mycoplasma Detection Kit (Lonza, LT07-118) every 2-3 months.

Generation of the CORD21-P1c and -P2c heterozygous isogenic control iPSCs

The generation of CORD21-P1c and CORD21-P2c isogenic control iPSCs was enabled by the single nucleotide CRISPR/Cas9 correction of the c.140delG and the c. 131G>A mutations present in the CORD21-P1 and -P2 iPSCs, respectively. Target gRNA and ssODN sequences were designed using the Benchling CRISPR online tool (https://www.benchling.com/crispr) (**Table S1**). gRNA was generated using the GeneArt Precision gRNA Synthesis Kit (Thermo Fisher Scientific, A29377) following the

manufacturer's instructions. ssODN was synthesized by Thermo Fisher Scientific. Nucleofection was performed using P3 Primary Cell 4DNucleofectorTM X Kit according to the manual's instructions (Lonza, V4XP-3024). To boost the efficiency of HDR recombination, ~ 1x10^6 CORD21-iPSCs were treated with 10 µM of NHEJ inhibitor SRC-7 (Sigma Aldrich, SML1546) for 8 hours prior to nucleofection. 200pM gRNA and 100pM TrueCut™ Cas9 Protein v2 enzyme (Thermo Fisher Scientific, A36497) were combined at room temperature (RT) for 10 minutes to allow for the formation of ribonucleoprotein complexes (RNPs). iPSCs were dissociated using StemPro Accutase for 5 minutes at 37° C (Gibco, A11105- 01) and centrifuged in the presence of 10µM Rock inhibitor (Y-27632, Fischer scientific, CD0141). gRNA/Cas9 RNPs and 200pM ssODN were added to iPSCs (Cas9 to gRNA/ssODN 1:2 ratio) and nucleofection was performed on a 4D-Nucleofector™ (Lonza). Matrigel™-coated Petri dishes were seeded with a drop of nucleofected solution and single cells were cultured in the presence of 10µM SRC-7 and 10µM Rock inhibitor. Medium was replaced within 24 hours and replenished every two days. Upon expansion of single-cell colonies, individual colonies were cultured on Matrigel™-coated 24-well plates followed by restriction digest analysis (CORD21-P1c, *HpyCH4V*, NEB, R0620L; CORD21-P2c, *Alu I*, NEB, R0137S). Clones showing correct band sizes were further sequenced to corroborate the successful correction of *DRAM2* mutant alleles (primers shown in **Table S1**).

Off-target sequencing

The online platform http://www.rgenome.net/cas-offinder/ was used to identify genomic regions bearing high sequence homology to the sites of CRISPR/Cas9 correction. Query sequences were allowed a maximum of up to three mismatches. Top 10 sequences exhibiting the highest sequence homology were selected and primers enclosing the respective genomic regions were designed for off-target sequencing as shown in **Table S1**.

Retinal Organoid Differentiation

iPSCs cultured in 6 well-plates were washed with PBS, dissociated using Accutase at about 80%-90% confluence (Gibco, Thermo Fisher Scientific) and seeded at a density of 7000 cells/well on U-bottom Lipidure pre-coated 96-well plates (AMSbio) in the presence of 10 µM ROCK inhibitor (Y27632, Tocris) (D-2). Upon formation of embryoid bodies (D0), differentiation medium was added to each well (41% IMDM, 41% HAM's F12, 15% KOSR, 1% GlutaMAX, 1% Chemically Defined Lipid Concentrate, 1% Pen/Strep (Thermo Fisher Scientific), and 225 µM 1-thioglycerol (M6145; Sigma)) and cultures were fed by carrying out half-media changes every two days. Differentiation medium was supplemented once with 2.25nM BMP4 (R&D, 314-BP-050/CF) at day 6, and medium was changed every three days until day 18. Subsequently, ROs were cultured in maintenance media (DMEM/F12, 10% FBS, 1%

GlutaMAX, 1% N2, 1% Pen/ Strep, 0.1 mM Taurine, 0.25 µg/ mL Fungizone), whereby 0.5 µM retinoic acid (RA) was added from day 18 to day 120 (Sigma Aldrich, R2625). Cultures were maintained by partial medium changes three times a week. A schematic of the RO differentiation protocol is shown in **Figure 1A**.

Immunofluorescence analysis of ROs

The organoid sections were left to dry at RT for an hour and washed 3x5 minutes with PBS (Thermo Fisher Scientific) to remove any remaining OCT. To minimize non-specific binding, sections were blocked for 1 hour at RT with a solution of PBS containing 10% goat serum and 0.3% Triton-X100. Sections were then double stained overnight at 4°C in a humidifier chamber using primary antibodies diluted in AD (Antibody diluent) buffer (0.001% BSA-PBS and 0.3% Triton-X, Sigma Aldrich, USA) (**Table S1**). Following incubation overnight, RO sections were washed 3x 10 minutes with AD buffer. Secondary antibodies diluted in PBS (1:1000) (**Table S1**) were applied to the sections and incubated for 2 hours at RT. Finally, the sections were washed three times with PBS for 15 minutes. Nuclei were counterstained with a 1:1000 dilution of Hoechst (cat. No. 33342, Thermo Fisher Scientific) in Vectashield (cat. No. H-1000-10, Vector Laboratories, CA). The slides were then covered with 24x60 mm coverslips, sealed on each side with nail polish, and stored at 4°C in the short term. For quality control, sections stained only with a secondary antibody were analysed for each staining to ensure the specificity of the fluorescent antibody signal. High-resolution fluorescence imaging was facilitated by optical sectioning on an Axio Imager microscope (Zeiss, Apotome, 10-15 Z-stacks). Images were acquired as maximum intensity projections on Zen (Zeiss). Quantification of immunostained RO sections were carried out on MATLAB (Mathworks) based on a protocol by Dorgau et al. (2019).

RPE differentiation

The differentiation procedure was commenced at 100% iPSC confluency (**Figure 1E**). During the initial 7 days of differentiation (D0-D7), the cells were cultured in DMEM/F-12, GlutaMAX™ medium (Thermo Fisher Scientific, 10565018) supplemented with 50µM β-mercaptoethanol (Sigma-Aldrich, M3148), 1xMEM NEAA (Thermo Fisher Scientific, 11140068), 20% KnockOut Serum Replacement (Thermo Fisher Scientific, 10828028) and 10 mM Nicotinamide (Sigma Aldrich, N0636). From day 7 to day 14 (D7-D14), nicotinamide was replaced by 100 ng/mL Activin A (Preprotech, 12014E-250UG). Between D14-D42, Activin A was substituted by 3 µM CHIR99021 (Sigma-Aldrich, SML1046). CHIR99021 supplementation was withdrawn at D42-84. Nascent RPE cells were allowed further time to mature in DMEM/F-12, GlutaMAX™ (Thermo Fisher Scientific, 10565018) medium containing 50µM β-mercaptoethanol (Sigma-Aldrich, M3148), 1xMEM NEAA (Thermo Fisher Scientific, 11140068) and 4%

KnockOut Serum Replacement (Thermo Fisher Scientific, 10828028). Fully mature and pigmented RPE cells seeded on hanging inserts showing TEER values > 250 Ω cm^2 were used for experiments described in this study.

Immunofluorescence analysis of RPE cells

Immunofluorescence analyses of RPE cells were performed as qualitative experiments using Zen software (Zeiss). In short, transwells were rinsed in PBS and incubated with 4% PFA for 30 minutes. The tissue was subsequently washed 3x5 minutes with PBS and flat RPE sheets were cut into multiple pieces. Additional fixation by methanol was performed at 4°C for 20 minutes when staining for tight junctions (ZO-1) and Collagen IV. To remove melanin pigment from the RPE a bleaching procedure was carried out using a Melanin Bleach kit (cat. No 24883, Polysciences), following the manufacturer's instructions. To minimize non-specific binding, RPE were then blocked and permeabilized simultaneously for 1 hour in PBS containing 10% Donkey Serum (cat. No. 7332100-LAM, Stratech) and 0.3% Triton-X-100. Primary antibodies were diluted in PBS containing 0.1% Triton-X-100 and 1% Donkey Serum-PBS and applied at 4°C overnight. The tissue was subsequently rinsed with PBS (3x5 minutes). Secondary antibodies diluted in PBS were incubated for 1 hour at RT. RPE sections were washed 3x5 minutes in PBS and counterstained for nuclei using Hoechst (1:1000 in PBS) for approximately 20 minutes. After an additional PBS wash, RPE sections were mounted on slides using Vectashield®. A secondary antibody only control was included in each set of experiments. For all antibody details please refer to **Table S1**.

Transepithelial electrical resistance (TEER)

Transepithelial electrical resistance was routinely conducted as a functional measurement of RPE barrier function using a volt-ohm meter (Millipore, MERS00002). RPE cells were equilibrated to RT, and a sterilized electrode was placed on either side of the transwell membranes. To determine unit area resistance (Ω · cm2), values pertaining to inserts containing media but devoid of cells (blanks) were subtracted from sample readings. Obtained values were multiplied by the surface area of the insert (0.33 cm^2 for a 24-well plate insert). RPE monolayer was considered mature for further analyses at TEER > 250 Ω cm^2. Measurements were taken in triplicates every two to three weeks.

Phagocytosis assay

<u>Labelling of photoreceptor outer segments (POS) with fluorescein isothiocyanate</u>

Bovine rod POSs (InVision BioResources, 98740) were labelled with 0.4mg/mL fluorescein isothiocyanate (FITC) (Sigma Aldrich, F7367) in basal RPE medium. The light sensitive POS solution was

placed on a shaking incubator shielded from light for 1 hour at RT. FITC-labelled POSs were washed 3x5 minutes with PBS, reconstituted at 10^6 POSs/mL in 73mM sucrose-PBS (Sigma Aldrich, S0389) and stored at -80°C.

<u>Flow cytometry analysis of phagocytic activity</u>

FITC-labelled POS were diluted in 10% FBS containing RPE medium and incubated with RPE on transwell inserts for 4 hours at 37°C. RPE cells were rinsed with PBS and dissociated into a single-cell suspension using TrypLE™ Select Enzyme (10x). The live cell dye DRAQ5™ (Abcam, ab108410) was resuspended in flow buffer (2% FBS in PBS) at a ratio of 1:40 and incubated with the dissociated cells for 10 minutes at 37°C. To quench any residual FITC fluorescence, cells were treated with 0.2% Trypan Blue for 10 minutes (Sigma Aldrich, 93595). Samples were centrifuged and further rinsed with 2% FBS-PBS. Flow cytometry analysis was conducted on a BD™ LSR II flow cytometer (BD Biosciences) with 10,000 events acquired for each sample. A sample kept at 4°C served as a negative control.

POS treatment of RPE cells

RPE were treated with 20 POSs/cell every day for 14 days and subjected to transmission electron microscopy analysis. An untreated sample was included as an internal control for each RPE cell line.

ELISA Detection of VEGF and PEDF secretion

A 96-well microplate was coated overnight with diluted Capture Antibody. Unbound antibodies were removed by washing and plate was blocked to prevent non-specific binding using Reagent Diluent at room temperature (RT). Following a round of washing, 100 µL of basal RPE media samples and standards were applied to the wells and incubated for 2 hours at RT. Following an additional washing step, the wells were treated with 100 µL Streptavidin-HRP for 20 minutes at RT in the dark. The plate was rinsed further to remove unbound HRP. To facilitate detection, substrate solution was added to the samples for 20 minutes. The reaction was terminated by the addition of a Stop Solution and optical density was measured at 450 nm using a Varioskan LUX Multimode Microplate ELISA reader (Thermo Fisher Scientific). An analogous procedure was carried out to determine PEDF levels in the apical compartment of RPE cells (insert media) using a Human Serpin F1/PEDF DuoSet ELISA kit by adhering to the manual’s instructions (Biotechne, DY1177-05).

PCR

<u>RNA extraction</u>

RNA extraction from RO pellets was performed in a laminar flow hood. The hood was UV sterilized and decontaminated from RNA nucleases using RNaseZap™ (Thermo Fisher Scientific, AM9780). RNA was extracted using the ReliaPrep™ RNA Cell Miniprep System following the manufacturer's instructions (Promega, Z6012).

cDNA synthesis

First strand cDNA synthesis was initiated by converting up to 5 µg of RNA in a 5 µL reaction containing primer Oligo(dT)15 and Nuclease-Free Water (NFW). The same amount of RNA was converted across all biological replicates based on the sample showing the lowest RNA concentration. Samples were placed onto a pre-heated block at 70°C for 5 minutes and subsequently pre-chilled at 4°C for another 5 minutes. Each of the samples was centrifuged to remove lid condensation and placed briefly on ice. A reverse transcriptase mix was prepared using GoScript™ 5X Reaction buffer, $MgCl_2$, dNTPs, RNasin Ribonuclease Inhibitor and GoScript Reverse Transcriptase in accordance with the guidance manual. 5 µL RNA and 15 µL of the mix were then carefully mixed and RNA was converted to cDNA at the thermocycler conditions outlined in the manual. cDNA was stored at -20°C until further use.

RT-qPCR

To determine *DRAM2a* isoform expression by RT-qPCR, RNA samples were cleared from any potential DNA contamination using a TURBO DNA-free™ kit (Thermo Fisher Scientific, AM1907). Equal amounts of cDNA template were used to set up a 384-well qPCR reaction using a GoTaq qPCR Master mix kit (Promega, A6002) on Quantstudio™ 7 Flex Real-Time PCR system (Applied Biosystems). Data were normalized to the expression of *GAPDH* and gene expression was calculated using the $2^{(-\Delta\Delta CT)}$ method. Samples were run in triplicates and data were presented as means + SEM. *DRAM2* oligonucleotide sequences are shown in **Table S1**.

DRAM2 siRNA knockdown

To improve siRNA uptake efficiency, day 230 WT ROs were dissected and cultured on Matrigel™ Growth Factor Reduced Basement Membrane Matrix (Corning), and poly-L-ornithine (10 µg/mL) (Sigma Aldrich, A-004-C) coated 24-well plates for a single round of passaging. siRNA transfection was performed at 50% confluence using a Lipofectamine™ RNAiMAX Transfection reagent (Thermo Fisher Scientific, 13778030). 20µM *DRAM2* Silencer Select siRNA (4392420, Assay ID: s43281) and scrambled control (Thermo Fisher Scientific, 4390849) were incubated with the dissected ROs in an antibiotic and serum-free maintenance media for 72 hours and collected for qPCR and WB analysis.

Western blot

BCA protein assay

Cell pellets stored at -80°C were lysed for 5 minutes at RT in PhosphoSafe™ extraction buffer (Merck Millipore, 71296) supplemented with protease inhibitor cocktail Complete Mini EDTA-free (Roche, 04693159001). Rigorous pipetting was alternated by vortexing and rest on ice for 20 minutes until samples were completely lysed. Samples were spun at 4°C for 10 minutes at 1000xg, and the concentration of the sample supernatants was determined using a Pierce™ BCA Protein Assay Kit (Thermo Fisher Scientific, 23225). Absorbance was measured at 562 nm on a Varioskan LUX Multimode Microplate Reader (Thermo Fisher Scientific).

Media sample collection and concentration

Media samples were collected for ROs at D220 and from RPE which had been starved for 7 days. Prior to use, Amicon 10 kDa MWCO ultrafiltration units (Merck, UFC801096) were washed with 4 mL sterile distilled water and centrifuged for 10 minutes at 3000xg. 5 mL of media was loaded onto membranes and concentrated to a final volume of 500 µL, whereby protease inhibitors cocktail was added to each sample (Complete Mini EDTA-free, Roche, 04693159001).

SDS-PAGE

4x NuPAGE™ LDS Sample Buffer (Thermo Fisher Scientific, NP0007) and 10x NUPAGE Sample reducing agent (Thermo Fisher Scientific, NP0004) were added to 10 µg of total protein lysate. Samples were subsequently heated for 10 minutes at 70°C and loaded onto precast 4-12% gradient polyacrylamide Bis-Tris gels (Thermo Fisher Scientific, NP0321PK2, WG1403BOX). NuPAGE™ MES SDS Running Buffer (20x) (Thermo Fisher Scientific, NP0002) and MOPS SDS Running Buffer (20x) (Thermo Fisher Scientific, NP0001) were used for the separation of small (<70kDa) to large (>70kDa) molecular targets, respectively. PageRuler™ Plus Prestained Protein Ladder 10 to 250kDa (Thermo Fisher Scientific, 26619) or SeeBlue™ Plus2 Prestained Protein standard (Thermo Fisher Scientific, LC5925) served as molecular weight reference. Please refer to **Table S1** for a comprehensive description of the Western blot conditions for individual protein targets.

Gel transfer

Dry transfer was performed on an iBlot 2 Dry Blotting system (Thermo Fisher Scientific) using Blot™ 2 Transfer PVDF Stacks (Thermo Fisher Scientific, IB24002 and IB24001) (20V for 1 minute, 23V for 4 minutes and 25V for 2 minutes). Transfer of autophagy markers, including DRAM2, was carried out at 10V for a total of 7 minutes.

Reversible total protein staining

Equal loading of protein samples for WB was corroborated using the Pierce™ Reversible Protein Stain Kit following the manual's instructions (Thermo Fisher Scientific, 24585). PVDF membranes were washed with ultrapure ddH20 and incubated with Memcode™ Sensitizer for 2 minutes under shaking conditions. Subsequently, membranes were stained with a total protein stain for 1 minute and rinsed three times with Memcode™ Destain. Rigorous washing was carried out on a shaker with a 1:1 Methanol/Destain solution, followed by an additional brief 8 washes with ddH20 to reduce the background staining produced by the reversible total protein stain. Membranes were imaged colorimetrically on an Amersham Imager 600 (GE Healthcare). Finally, the staining was erased following a 10 minute incubation with a 1:1 Eraser/Methanol solution and membranes were briefly washed with ultrapure ddH20.

Membrane blocking and antibody incubation

Membranes were blocked for non-specific binding with 5% milk TBST (Tris-buffered saline with 0.1% Tween®20 detergent) at RT for 1 hour. Incubation with primary antibodies in 5% milk TBST was carried out on a rotating platform overnight at 4°C (**Table S1**). Membranes were then washed 3x 5 minutes with TBST at RT. Secondary antibodies were incubated at 1:1000 for 1 hour at RT (**Table S1**) and washed 3x 5 minutes with TBST. To enable visualization, chemiluminescence substrate was prepared using the SuperSignal™ West Pico PLUS Chemiluminescent Substrate kit (Thermo Fisher Scientific, 34579). Image acquisition was carried out on an Amersham Imager 600 (GE Healthcare). Band quantitation analysis was performed on Image J (NIH).

Detection of low-abundance protein

For detection of low-abundance proteins, PVDF membranes post protein transfer were washed with ultrapure ddH20 briefly and pre-treated with an antigen pretreatment solution for 10 minutes at RT (SuperSignal™ Western Blot Enhancer, 46640). After blocking for non-specific binding with 5% milk TBST, membranes were rinsed in TBST for an additional 5 minutes and incubated with primary antibody in antibody diluent overnight at 4°C (SuperSignal™ Western Blot Enhancer). Following 3 washes with TBST (3x 20 minutes), secondary antibodies were incubated for 1 hour at RT at 1:20 000-1:100 000. For optimized signal detection membranes were rigorously washed in TBST for an additional 1 hour prior to being visualized using the SuperSignal™ West Femto Maximum Sensitivity Substrate kit (Thermo Fisher Scientific, 34094). Please refer to **Table S1** for more details.

Lysosomal activity assays

CTSD kinetic activity assay

To preserve enzymatic activity the RPE and RO samples were lysed in native buffer (0.2M Triethylammonium bicarbonate buffer, Sigma Aldrich, T7408) containing protease inhibitors cocktail Complete Mini EDTA-free (Roche, 04693159001). Protein concentration was measured using the Pierce™ BCA Protein Assay Kit (Thermo Fisher Scientific, 23225). 50 µM MCA (7-Methoxycoumarin-4-acetic acid) diluted in assay buffer was used to generate a standard curve (0-25 µM) (Thermo Fisher Scientific, 265301). The kinetic assay was performed on black clear bottom 96-well plates (Thermo Fisher Scientific) in a total volume of 100 µL assay buffer. 5 µg protein lysate was diluted in 50mM sodium acetate assay buffer pH 4.0 (Thermo Fisher Scientific, AM9740). Protein lysate pre-incubated with 0.2mg/mL pepstatin inhibitor at 37°C for 15 minutes (Enzo Life Sciences) served as a negative control. Immediately prior to the initiation of the kinetic assay, Cathepsin D & E substrate (Enzo Life Sciences) was applied at a final concentration of 80µM. Fluorescence signal kinetics were recorded every 5 minutes over the course of 20 minutes upon addition of the substrate at 37°C with intermittent shaking. Measurements were taken at 320nm/400nm excitation/emission spectra using a Varioskan LUX Multimode Microplate Reader (Thermo Fisher Scientific) (Kurzawa-Akanbi et al., 2021).

Glucosylceramidase and Alpha-mannosidase activity assays

The glucosylceramidase activity assay (Abcam, 273339) was conducted in a total volume of 160 µL. 20µM 4-Methylumbelliferone Standard (4-MU) prepared in Assay Buffer was used to generate a standard curve (0-20µM). The samples were lysed using the provided Assay Buffer solution and concentration was measured using a Pierce™ BCA Protein Assay Kit (Thermo Fisher Scientific, 23225). 20x diluted substrate was added to 30 µg of protein lysate diluted in Assay Buffer. Samples were incubated on a white flat bottomed 96-well plate (Appleton Woods Ltd, SS246) at 37°C degrees for 30 minutes under minimal light exposure. Following incubation, 100 µL Glucosylceramidase Stop buffer was added to terminate the reaction in all samples. Fluorescence intensity was measured at 360nm/445nm excitation/emission spectra in end-point mode at 37°C. Alpha-mannosidase activity in the samples was determined analogously using a fluorometric end-point mode Alpha-mannosidase activity assay kit (Abcam, 282917).

Proteomics

Protein digestion

10 µg of total RO protein (5 µL) (n=3-4 different differentiation experiments/sample) were digested using S-Trap micro spin columns (Protifi, NY, USA). Each sample was denatured in 25 µL 5% SDS 50mM Triethylammonium bicarbonate (TEAB) (pH 8.5). The proteins were then reduced with 20mM DTT at 65°C for 30 minutes and alkylated with 40mM iodoacetamide at RT in the dark for 30 minutes. Acidification was achieved by adding 27.5% phosphoric acid to a final concentration of 2.5% (v/v). Protein sample was subsequently bound onto the micro spin columns in 6 volumes of loading buffer (90% methanol 100mM TEAB pH 8.0) and spun at 4000xg for 30s. Columns were rinsed 3x with loading buffer and flow-through was discarded. Protein digestion with trypsin (Worthington) was performed overnight at 37°C in 50mM TEAB pH 8.5, at a ratio of 10:1 protein to trypsin. Peptide elution was carried out over three consecutive washes: first 50 µL 50mM TEAB, second 50 µL 0.2% formic acid and third 50 µL 50% acetonitrile with 0.2% formic acid. The solution was subsequently frozen, desiccated in a vacuum concentrator and reconstituted in 0.2% formic acid.

The digestion of RPE cell lysates followed a similar protocol with some modifications. 15 µg total protein RPE sample (n=7 different differentiation experiments/sample) were adjusted to 23 µL and equal volumes of 2x concentrated S-trap lysis buffer was added (10% SDS in 100mM TEAB pH 8.5]. RPE samples were prepared using the same general steps of reduction, alkylation, and acidification. Digestion with trypsin was carried out for 1.5 hours at 47°C and peptides were eluted as outlined above.

Data Acquisition and processing

LCMS analysis for RO and RPE cells was conducted on an Exploris 480 Quadrupole-Orbitrap Mass Spectrometer (Thermo Fisher Scientific) and a Orbitrap Fusion™ Lumos™ Tribrid™ Mass Spectrometer (Thermo Fisher Scientific), respectively. For RO LCMS, equivalents of 1 µg of each peptide were loaded onto an Acclaim PepMap100 C18 LC Column (Thermo Fisher Scientific) and separated on a 75µm x25 cm C18 column (Thermo Fisher Scientific). For the loading of 1 µg RPE a 300µm x 5mm C18 PepMap trap cartridge (Thermo Fisher Scientific) was utilized and a 75µm x 50cm C18 column for separation (Thermo Fisher Scientific). The data was queried against the protein sequence database available on https://www.uniprot.org/uniprot/?query=proteome:UP000005640 using MaxQuant v2.0.3.0 (ROs), and Spectronaut v16.1 (Biognosys) (RPE) based on default settings such as cysteine alkylation: iodoacetamide, digestion enzyme: trypsin, and variable modifications: Oxidation (M), Acetyl (Protein N-term). Statistically significant changes in relative protein abundance were identified using modified

ANOVA (FDR<0.05, S0=0.1) followed by Tuckey's post hoc test (FDR<0.05). Metascape annotation of common pathways has been performed using https://metascape.org/gp/index.html#/main/step1 (Zhou et al., 2019).

Lipidomics

Samples were extracted in chloroform/methanol (2/1, v/v) and centrifuged at 1700 x *g* for 10 minutes. Phase partitioning was facilitated by the addition of 0.1 M KCL, whereby the lower organic phase containing the lipid fraction was retained. The lipid-enriched phase was subsequently dried in the presence of nitrogen gas prior to being reconstituted in methanol containing 5 mM ammonium formate. Lipidomic analysis was carried out under positive and negative ion modes over the mass to charge (*m/z*) range 250-2000 at a resolution of 100,000 using a Thermo Exactive Orbitrap mass spectrometer equipped with a heated electrospray ionization (HESI II) probe coupled to a Thermo Fisher Scientific ultimate 3000 RSLC system. Separation of lipids by column chromatography was performed using a Thermo Hypersil Gold C18 column (1.9µm; 2.1 mm × 100 mm) maintained at 50°C. Mobile phase A comprised an aqueous solution containing 10 mM ammonium formate and 0.1% (v/v) formic acid. Mobile phase B was made up of 90:10 isopropanol/acetonitrile (ACN) containing 10 mM ammonium formate and 0.1% (v/v) formic acid. The initial gradient conditions were 65%A/35%B. An increase the gradient of phase B was applied from 35% to 65% over 4 minutes, followed by 65%-100% over 15 minutes, with a hold for 2 minutes before re-equilibration to the starting conditions over 6 minutes. The gradient flow occurred at a rate of 400 µL/minute.

Transmission electron microscopy (TEM)

Samples were initially fixed in 2% glutaraldehyde 0.1 M sodium cacodylate and further subjected to an additional fixation by 2% osmium tetroxide. Samples were then dehydrated by a gradual increase. in acetone concentration, up to 100% then impregnated with increasing concentrations of epoxy resin. After 2x 100% resin steps, the samples were embedded in epoxy resin and polymerised at 60 degrees for 24 hours. Epoxy resin blocks were ultra-sectioned into slices of 70 nm thickness, collected on copper grids and further stained by uranyl acetate and lead citrate. TEM images were captured using a Hitachi HT7800 TEM microscope. TEM analyses entailed the blindfold selection of images to enable an unbiased evaluation of cellular morphology findings. Image segmentation and processing was conducted using the open-source Microscopy Image Browser software (Belevich et al., 2016).

References:

Belevich, I., Joensuu, M., Kumar, D., Vihinen, H. and Jokitalo, E. (2016) Microscopy Image Browser: A Platform for Segmentation and Analysis of Multidimensional Datasets. PLoS Biol, *14*, e1002340. https://doi.org/10.1371/journal.pbio.1002340.

Buskin, A. et al. (2018) Disrupted alternative splicing for genes implicated in splicing and ciliogenesis causes PRPF31 retinitis pigmentosa. Nat Commun. *9*, 4234. https://doi.org/ doi: 10.1038/s41467-018-06448-y.

Dorgau, B. et al. (2019) Decellularised extracellular matrix-derived peptides from neural retina and retinal pigment epithelium enhance the expression of synaptic markers and light responsiveness of human pluripotent stem cell derived retinal organoids. Biomaterials. *199*, 63-75. https://doi.org/10.1016/j.biomaterials.2019.01.028.

Kurzawa-Akanbi, M. et al. (2021) Neuropathological and biochemical investigation of Hereditary Ferritinopathy cases with ferritin light chain mutation: Prominent protein aggregation in the absence of major mitochondrial or oxidative stress. Neuropathology and Applied Neurobiology *47*, 26–42. https://doi.org/10.1111/nan.12634.

Kurzawa-Akanbi, M. et al. (2022) Retinal pigment epithelium extracellular vesicles are potent inducers of age-related macular degeneration disease phenotype in the outer retina. J Extracell Vesicles, *11*, 12295. https://doi.org/ 10.1002/jev2.12295.

Zhou, Y., Zhou, B., Pache, L., Chang, M., Khodabakhshi, A.H., Tanaseichuk, O., Benner, C. and Chanda, S.K. (2019) Metascape provides a biologist-oriented resource for the analysis of systems-level datasets. Nat Commun. *10*, 1523. https://doi.org/10.1038/s41467-019-09234-6.

Supplementary Figures

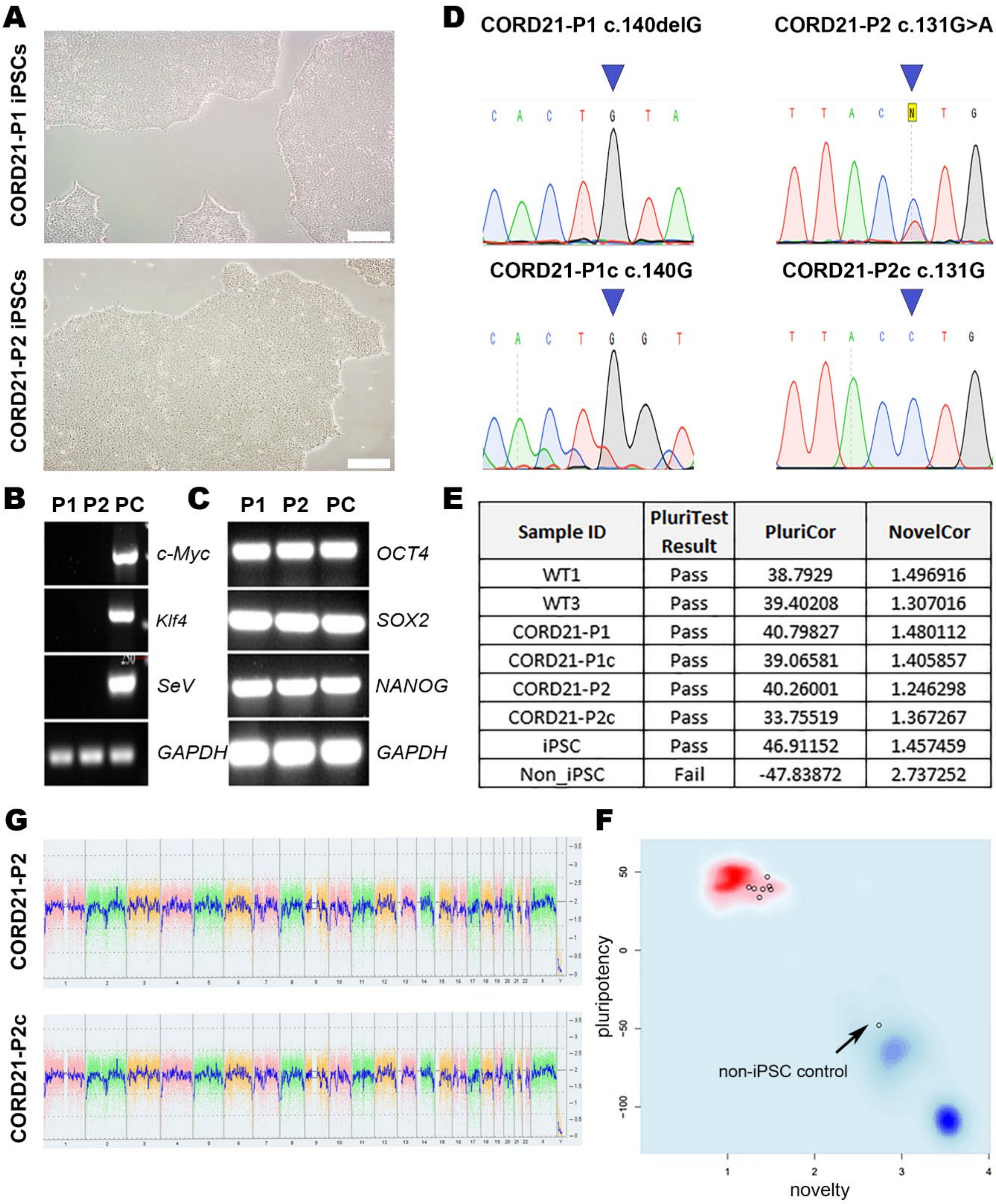

Sample ID	PluriTest Result	PluriCor	NovelCor
WT1	Pass	38.7929	1.496916
WT3	Pass	39.40208	1.307016
CORD21-P1	Pass	40.79827	1.480112
CORD21-P1c	Pass	39.06581	1.405857
CORD21-P2	Pass	40.26001	1.246298
CORD21-P2c	Pass	33.75519	1.367267
iPSC	Pass	46.91152	1.457459
Non_iPSC	Fail	-47.83872	2.737252

Figure S1. CORD21-iPSCs and the respective isogenic controls are pluripotent and lack genomic instabilities. (**A**) Brightfield images from the CORD21-P1 and -P2 iPSCs demonstrate a typical pluripotent stem cell colony-like morphology, scale bars=200 µm. (**B**) RT-PCR shows clearance of Sendai virus-associated transgenes in CORD21-P1 and -P2 iPSCs relative to positive control (PC) following iPSC reprogramming. (**C**) RT-PCR demonstrates expression of pluripotent marker genes (*OCT4*, *SOX2* and *NANOG)* relative to PC sample. *GAPDH* was used as a control. (**D**) DNA chromatograms show the absence of a guanine base at c.140 in CORD21-P1 and its respective presence in CORD21-P1c upon CRISPR-Cas9 correction. The heterozygous c.131G>A mutation present in CORD21-P2 is restored to the wild-type state in the CORD21-P2c isogenic control. (**E**) PluritestTM Table shows high pluripotency scores (PluriCor) for iPSC lines used in this study. (**F**) The pluripotency test plot is a visual representation of the samples based on pluripotency (y-axis) and the novelty score (x-axis) listed in the PluriTable (E). The x/y scatter plot shows the relative distribution of pluripotent (red) and non-pluripotent samples (blue) in the reference dataset. A non-iPSC sample indicated by arrow served as a negative control. (**G**) Whole genome view shows no chromosomal copy number abnormalities for somatic and sex chromosomes in CORD21-P2 and -P2c iPSCs. The smooth signal plot (y-axis) represents log2 ratios of microarray signal intensities, whereby a deviation from the normal chromosome copy number (CN=2) would indicate chromosomal copy number aberrations. Colours pertain to the raw chromosomal signal, whereby blue represents the normalized copy number signal.

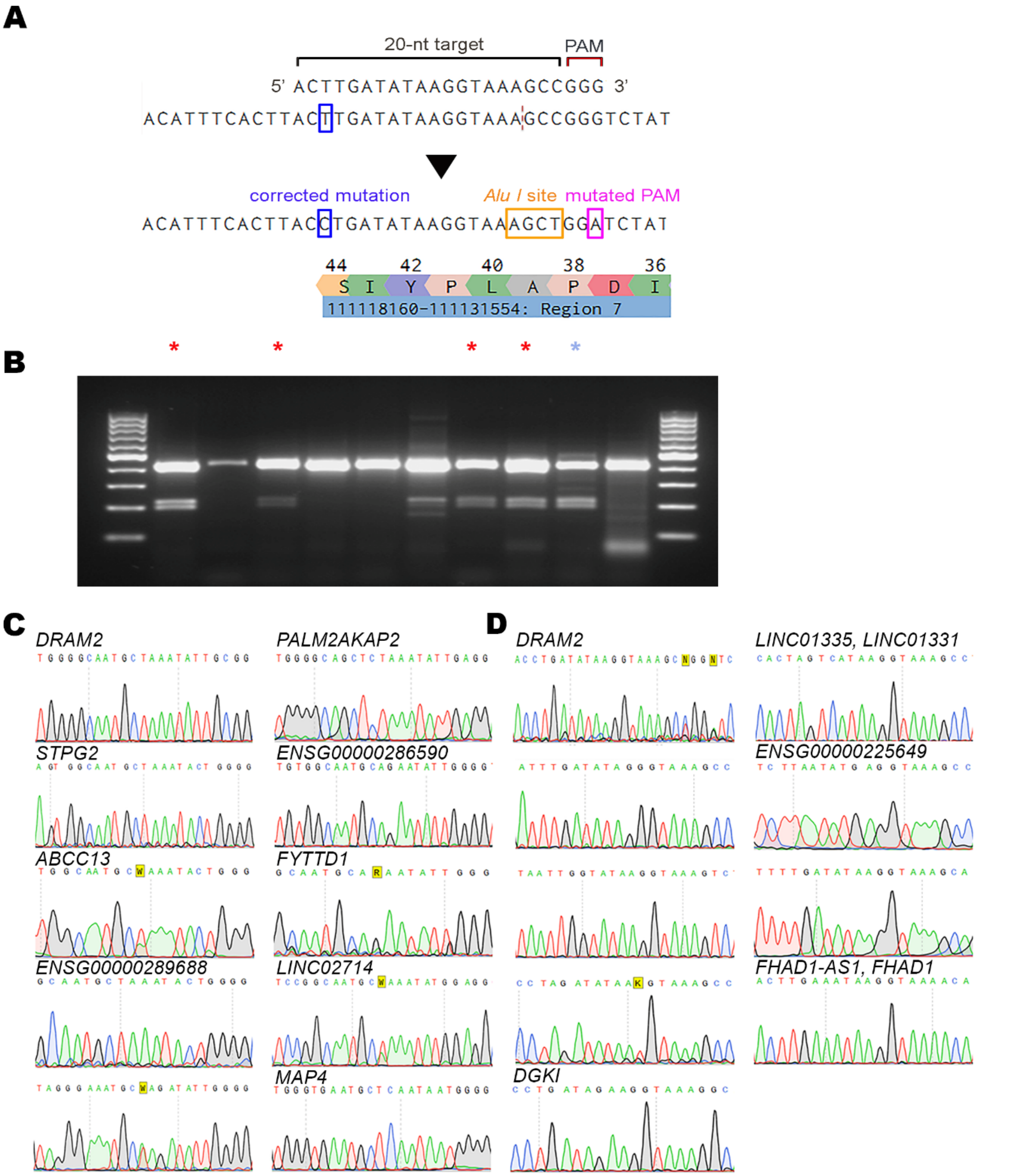

Figure S2. CRISPR-Cas9 *in situ* gene editing targets the *DRAM2* gene without off site effects in CORD21-P1c and -P2c iPSCs. (**A**) Sequence in the top panel shows gRNA used in CRISPR-Cas9 experiment (mutation site outlined by blue rectangle). Bottom panel shows genomic sequence following putative CRISPR-Cas9 correction of the c.131G>A mutation in CORD21-P2 iPSCs (corrected mutation outlined in blue, the *Alu I* and abrogated PAM sites marked in orange and magenta, respectively). (**B**) Representative gel image of CRISPR-edited clones following DNA extraction. iPSC clones marked by blue and red asterisks were selected as primary targets for Sanger sequencing due to producing expected band patterns of approximately 216 and 192 bps following *Alu I* restriction

digest. The clone marked by the blue asterisk corresponds to clone 72 which was used to generate the CORD21-P2c isogenic iPSC line. DNA chromatograms from Sanger sequencing confirm the absence of genomic alterations at sites homologous to the targeted genomic sequence as a result of the CRISPR-Cas9 editing of CORD21-P1c (n=10) (**C**) and CORD21-P2c iPSCs (n=9) (**D**). The name of genes covering these sequences can be seen in the top left corner of each chromatogram. Sites of homology were identified using Cas-OFFinder by allowing a maximum of three-mismatches in the targeted sequence (**Table S1**).

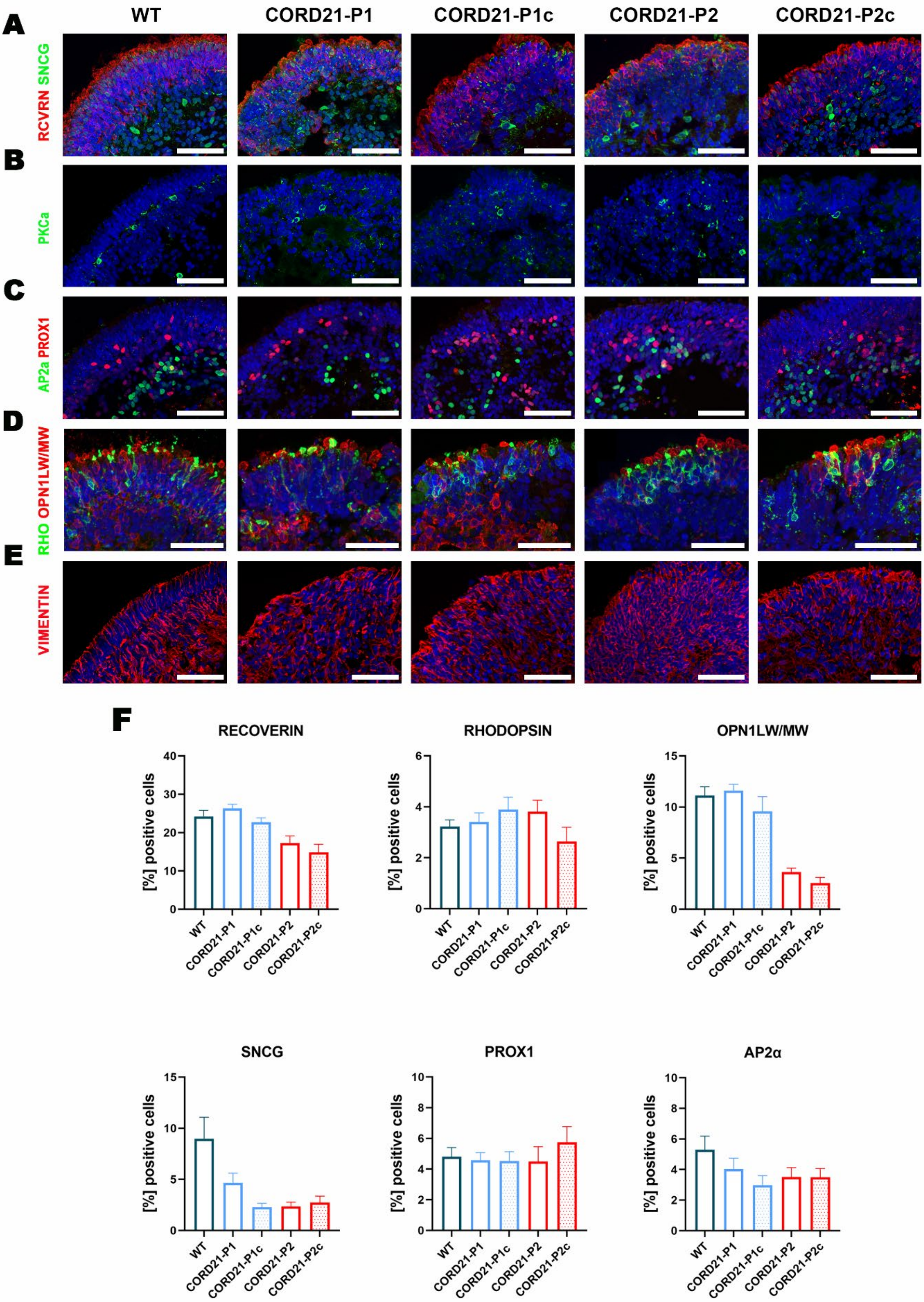
A
B
C
D
E
F
WT
CORD21-P1
CORD21-P1c
CORD21-P2
CORD21-P2c
RCVRN SNCG
PKCa
AP2a PROX1
RHO OPN1LW/MW
VIMENTIN
RECOVERIN
RHODOPSIN
OPN1LW/MW
SNCG
PROX1
AP2α
[%] positive cells

Figure S3. Characterisation of day 220 CORD21- and control-ROs by immunofluorescence (IF) analyses. IF experiment demonstrated the presence of all retinal cell types such as photoreceptor (**A**) (Recoverin, red) and ganglion cells (SNCG, green), (**B**) rod bipolar cells (PKCα, green), (**C**) horizontal (PROX1, red) and amacrine (AP2α, green) cells, (**D**) red/green cones (OPN1LW/MW, red) and rod photoreceptors (RHO, green) as well as (**E**) Müller glia (vimentin, red). Nuclei are counterstained with Hoechst (blue). These are representative examples from 15 ROs imaged from three different differentiation experiments/sample. Scale bars = 50µm for RCVRN/SNCG, PKCα, PROX1/AP2α and vimentin, and 20µm for RHO/OPN1LW/MW. (**F**) Quantification bar plots show % positive cells corresponding to the expression of each marker relative to the total number of cells. Data presented as mean + SEM (n=15 ROs imaged from three different differentiation experiments/sample).

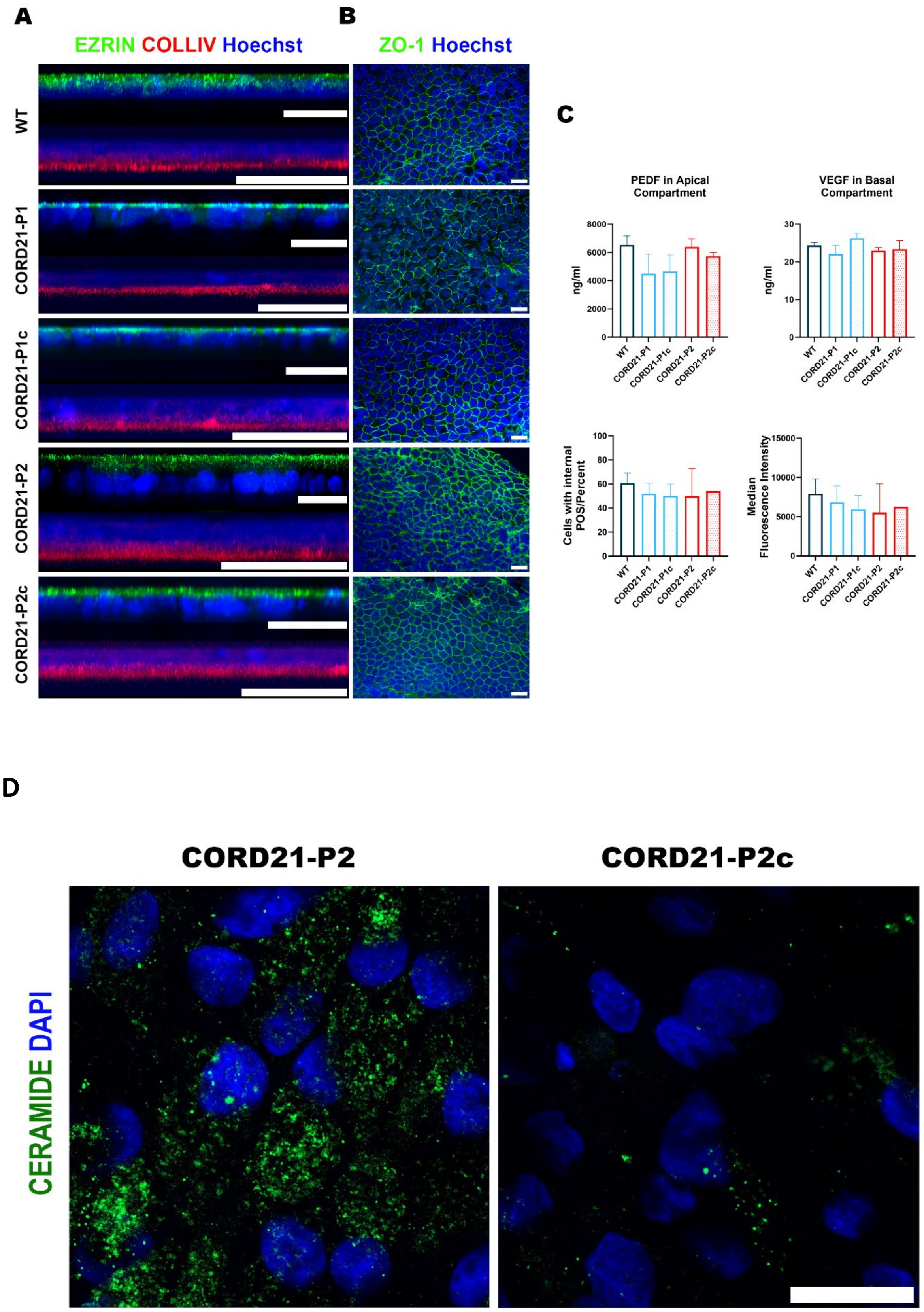
A
EZRIN COLLIV Hoechst
B
ZO-1 Hoechst
WT
CORD21-P1
CORD21-P1c
CORD21-P2
CORD21-P2c
C
PEDF in Apical Compartment
ng/ml
VEGF in Basal Compartment
ng/ml
Cells with internal POS/Percent
Median Fluorescence Intensity
WT
CORD21-P1
CORD21-P1c
CORD21-P2
CORD21-P2c
D
CORD21-P2
CORD21-P2c
CERAMIDE DAPI

Figure S4. RPE generation and characterisation by IF and ELISA assays. (**A**) CORD21- and control RPE cells display the apical expression of EZRIN and basal expression of Collagen IV (COL IV), scale bar=20µm. These are representative examples from 15 RPE transwells imaged from three different differentiation experiments/sample. (**B**) CORD21- and control RPE cells display the expression of the tight junction marker ZO-1, scale bar=20µm. (A, B) These are representative examples from 15 RPE transwells imaged from three different differentiation experiments/sample. (**C**) No significant differences in apical section of PEDF, basal secretion of VEGF and the ability to phagocytose photoreceptor outer segments (POSs) are observed between the CORD21- and control RPE cells. Data presented as mean + SEM (n=9 RPE transwells from three different differentiation experiments/sample). **(D)** Ceramide accumulation in CORD21-RPE cells. These are representative examples from 15 RPE transwells imaged from three different differentiation experiments. Scale bar=20µm.

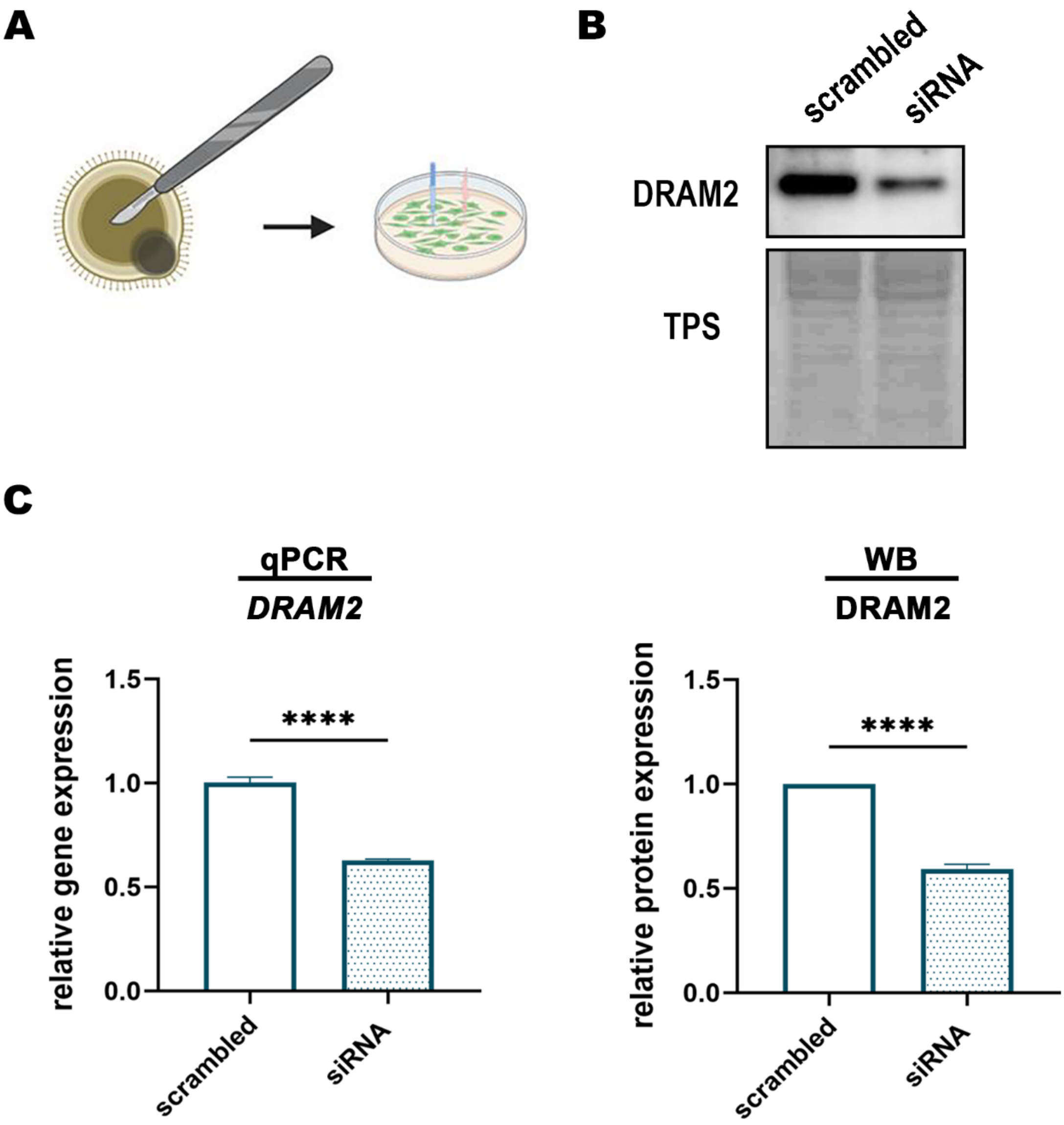

Figure S5. DRAM2 antibody validation and protein detection in ROs by western blot. (**A**) WT day 230 ROs were dissected and expanded on Matrigel™, and poly-L-ornithine coated 24-well plates. Treatment with 20µM *DRAM2* and scrambled siRNA was carried out for a period of 72 hours. (**B**) DRAM2 protein detection (~26kDa) in day 220 patient and control ROs. TPS stands for total protein stain showing equal sample loading. Quantitative analysis (bottom panel) confirms 50% reduction in DRAM2 protein expression. (**C**) Quantitative RT-PCR confirms *DRAM2* knockdown in siRNA treated ROs compared to scrambled control. (B-C) Data are presented as mean + SEM (n=3 different differentiation experiments each consisting of 24 ROs/sample). **** p<0.0001.

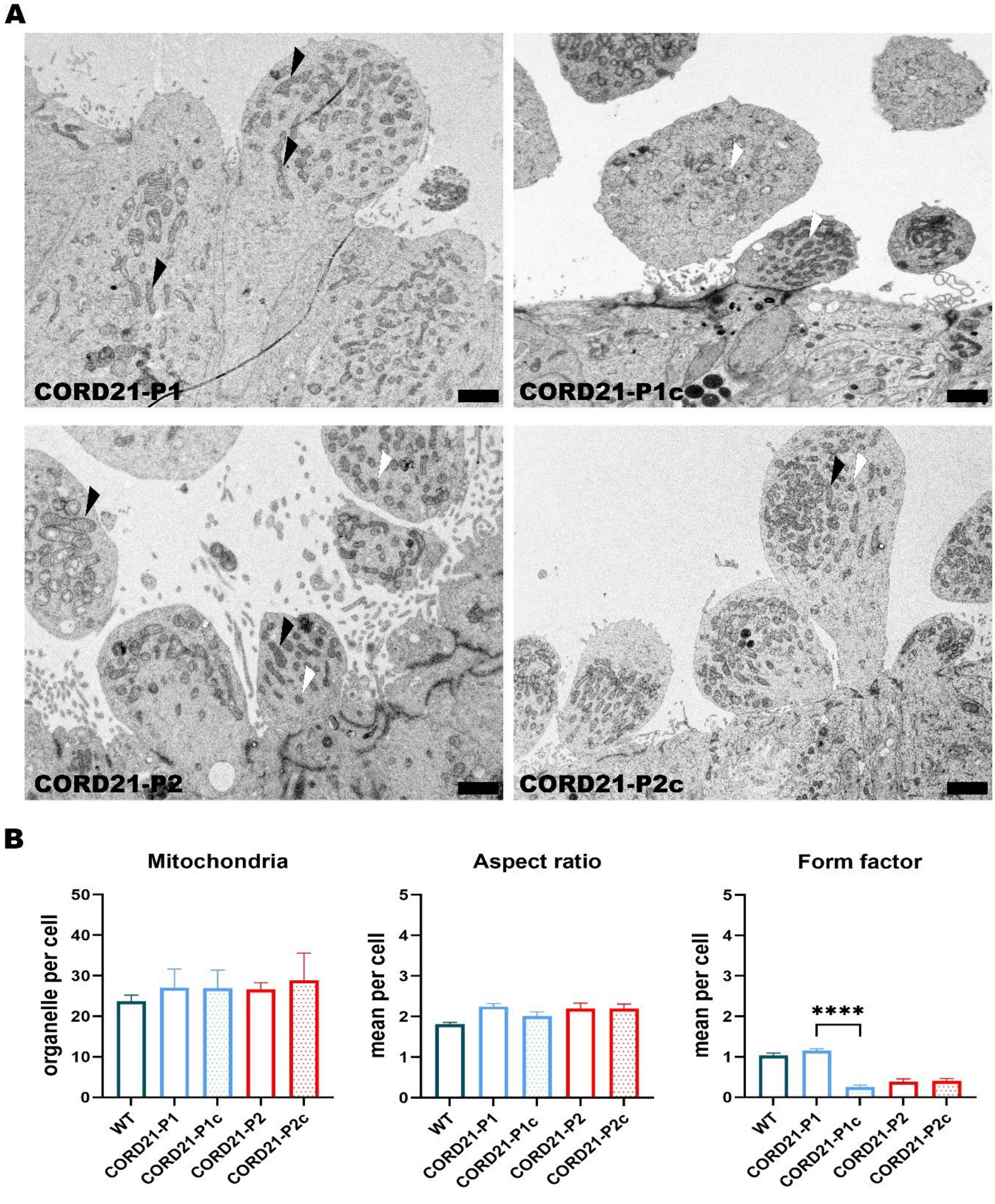

Figure S6. CORD21-P1-ROs show increased number of branched mitochondria relative to isogenic control. (**A**) CORD21-P1 image indicates the higher abundance of more branched mitochondria in photoreceptor cell bodies, and ISs as opposed to CORD21-P1c where less mitochondrial branching is apparent. Images from CORD21-P2 and CORD21-P2c demonstrate high levels of mitochondrial diversity, whereby both smaller and more elongated mitochondria are apparent in the photoreceptor cell bodies/inner segment. Elongated mitochondria are shown by black arrow heads and rounded mitochondria by white arrow heads, respectively. (**B**) Bar plots show no difference in mitochondrial

count per cell and aspect ratio. Significant differences are observed for form factor which indicates changed mitochondrial branching between CORD21-P1 and CORD21-P1c photoreceptors. Data are presented as mean + SEM. Mitochondrial segmentation analysis was carried out using the Microscopy Image Browser software (n=10 ROs from three different differentiation experiments/sample). Statistical difference for CORD21-P1vs -P1c is denoted by ****p<0.0001.

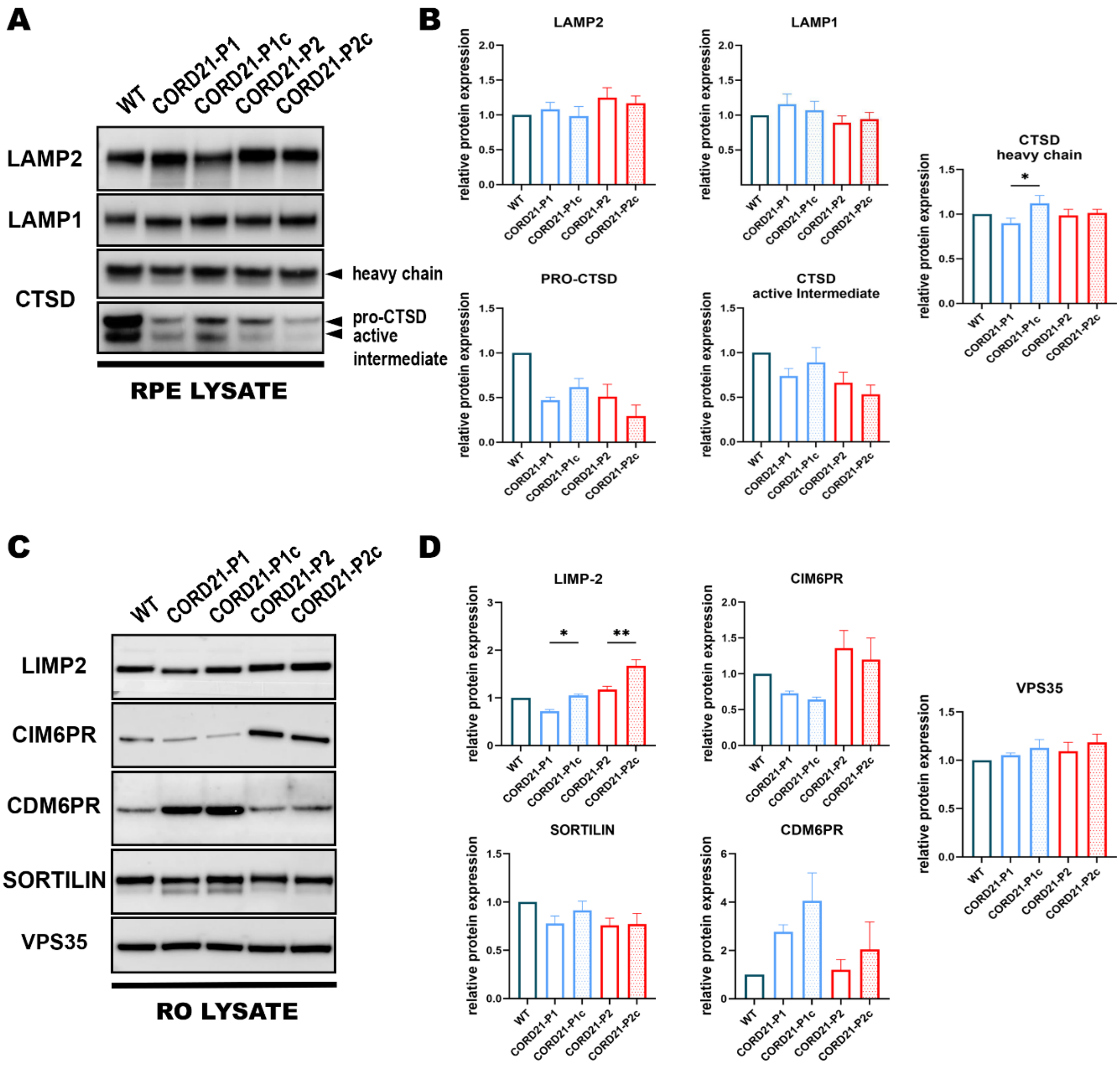

Figure S7. Additional screening of transport proteins suggests cellular transport dysfunction as indicated by the depletion of specific lysosomal receptors. (**A-B**) RPE cells show no differences in LAMP2 and LAMP1 expression and corroborate the downregulation of CTSD heavy chain in the lysates of CORD21--P1-RPE cells. Furthermore, no changes were observed for CTSD active intermediate. Data are presented as mean + SEM (n=3 different differentiation experiments each consisting of 2 wells of a 12-well plate of RPE cells/sample) and normalised to the WT sample. (**C-D**) Western blot analyses in day 220 RO lysates reveals a consistent downregulation in expression of GBA receptor LIMP2 in both CORD21-ROs. Conversely, no significant differences were established for the expression of CIM6PR, CDM6PR, VPS35 and sortilin. Data are presented as mean + SEM (n=3-4 differentiation experiments each consisting of 48 ROs/sample) and normalised to the WT sample. Equal protein loading was visualised by the total protein stain. Statistical comparisons for CORD21-P1vs -P1c and CORD21-P2vs-P2c are denoted by * p<.05, **p<.01.

Supplementary Table Legends

Table S1. A summary of CORD21 patient clinical information and the reagents used for CRISPR/Cas9 screening, RT-PCR, western blot and immunofluorescence analyses.

Table S2. A summary of proteomics analyses conducted in CORD21-ROs and RPE cells and the heterozygous isogenic controls.
