# Supplementary material for: Retinal cells derived from patients with DRAM2-dependent CORD21 dystrophy exhibit key lysosomal enzyme deficiency and lysosomal content accumulation
Source: Stem Cell Reports. 2024 Jul 3;19(8):1107–21. doi: 10.1016/j.stemcr.2024.06.002 (PMC11368688; doi:10.1016/j.stemcr.2024.06.002)
Supplement: Document S2. Article plus supplemental information [file mmc4.pdf]

Stem Cell Reports
Article

OPEN ACCESS

Retinal cells derived from patients with DRAM2-dependent CORD21 dystrophy exhibit key lysosomal enzyme deficiency and lysosomal content accumulation

Rozaliya Tsikandelova,[1] Eldo Galo,[1] Edvinas Cerniauskas,[1] Dean Hallam,[1] Maria Georgiou,[1] Rodrigo Cerna-Chavez,[1] Robert Atkinson,[1] Pavel Palmowski,[1] Florence Burté,[1] Tracey Davies,[2] David H. Steel,[1] Martin McKibbin,[3] Jacquelyn Bond,[3] Jennifer Haggarty,[4] Phil Whitfield,[5] Viktor Korolchuk,[1] Lyle Armstrong,[1] Chunbo Yang,[1] Birthe Dorgau,[1] Marzena Kurzawa-Akanbi,[1] and Majlinda Lako[1,6,*]
[1]Biosciences Institute, Newcastle University, Newcastle, UK
[2]Electron Microscopy Research Services, Newcastle University, Newcastle, UK
[3]Leeds Teaching Hospitals NHS Trust, Leeds UK and Leeds Institute for Medical Research, St. James's University Hospital, University of Leeds, Leeds, UK
[4]Shared Research Facilities, College of Medical, Veterinary and Life Sciences, University of Glasgow, Glasgow G12 8QQ, UK
[5]Glasgow Polyomics and Institute of Infection, Immunity and Inflammation, College of Medical, Veterinary and Life Sciences, University of Glasgow, Glasgow, UK
[6]Lead contact
*Correspondence: majlinda.lako@ncl.ac.uk
https://doi.org/10.1016/j.stemcr.2024.06.002

SUMMARY

Biallelic mutations in *DRAM2* lead to an autosomal recessive cone-rod dystrophy known as CORD21, which typically presents between the third and sixth decades of life. Although DRAM2 localizes to the lysosomes of photoreceptor and retinal pigment epithelium (RPE) cells, its specific role in retinal degeneration has not been fully elucidated. In this study, we generated and characterized retinal organoids (ROs) and RPE cells from induced pluripotent stem cells (iPSCs) derived from two CORD21 patients. Our investigation revealed that CORD21-ROs and RPE cells exhibit abnormalities in lipid metabolism, defects in autophagic flux, accumulation of aberrant lysosomal content, and reduced lysosomal enzyme activity. We identified potential interactions of DRAM2 with vesicular trafficking proteins, suggesting its involvement in this cellular process. These findings collectively suggest that DRAM2 plays a crucial role in maintaining the integrity of photoreceptors and RPE cells by regulating lysosomal function, autophagy, and potentially vesicular trafficking.

INTRODUCTION

Inherited retinal diseases (IRDs) are a primary cause of irreversible vision loss affecting 5.5 million people worldwide (Hanany et al., 2020). Next-generation sequencing has facilitated the identification of IRD-causing variants (Dockery et al., 2021; Neveling et al., 2012; Farrar et al., 2017) resulting in the annotation of a total of 341 IRD-associated genes (RetNet, the Retinal Information Network). Gene identification requires functional studies to prove their causative role and shed light onto pathogenic mechanisms. Validated human patient-derived retinal models are therefore crucial, underscored by the inability of murine models to fully recapitulate human retinal disease due to the lack of a macula (Volland et al., 2015), and the inconvenience of large animal models relating to higher cost, longer lifespan, and time required for disease manifestation (Winkler et al., 2020). The generation of patient induced pluripotent stem cell (iPSC)-derived retinal models, which are more representative of human retinal biology and display molecular features similar to those of the fetal human retina (Foltz and Clegg, 2019; Maeda and Takahashi, 2023), has advanced IRD understanding.

Biallelic mutations in the novel lysosome and autophagy regulatory gene, DNA damage regulated autophagy modulator 2 (*DRAM2*), are associated with an autosomal recessive form of retinal dystrophy known as CORD21 (OMIM # 616502), affecting both cones and rods (El-Asrag et al., 2015; Sergouniotis et al., 2015; Abad-Morales et al., 2019; Kuniyoshi et al., 2020; Krašovec et al., 2022). Most CORD21 patients present with macular involvement and loss of cone photoreceptors by the third decade of life. At more advanced stages, the disease presents with macular atrophy, peripheral retinal degeneration, and the inability to see in dim-light conditions (El-Asrag et al., 2015; Sergouniotis et al., 2015; Kuniyoshi et al., 2020; Abad-Morales et al., 2019). A total of 19 unique pathogenic *DRAM2* variants have been reported in fewer than 30 patients (Krašovec et al., 2022); however, the UK Biobank repository has an unpublished record of ~2,900 individuals with pathogenic or predicted pathogenic variants at the *DRAM2* locus, suggesting greater importance of *DRAM2* than currently recognized.

DRAM2 encodes a 266-amino-acid lysosomal membrane protein (O'Prey et al., 2009; Park et al., 2009), which facilitates the conversion of endogenous LC3-I (microtubule-associated protein light chain 3 I) to LC3-II (microtubule-associated protein light chain 3 II) (Yoon et al., 2012; Zeng et al., 2014) and the binding of lysosomal proteins LAMP1 and LAMP2 during autophagy (Kim et al., 2017). In the murine retina DRAM2 localizes to

This is an open access article under the CC BY license (http://creativecommons.org/licenses/by/4.0/).

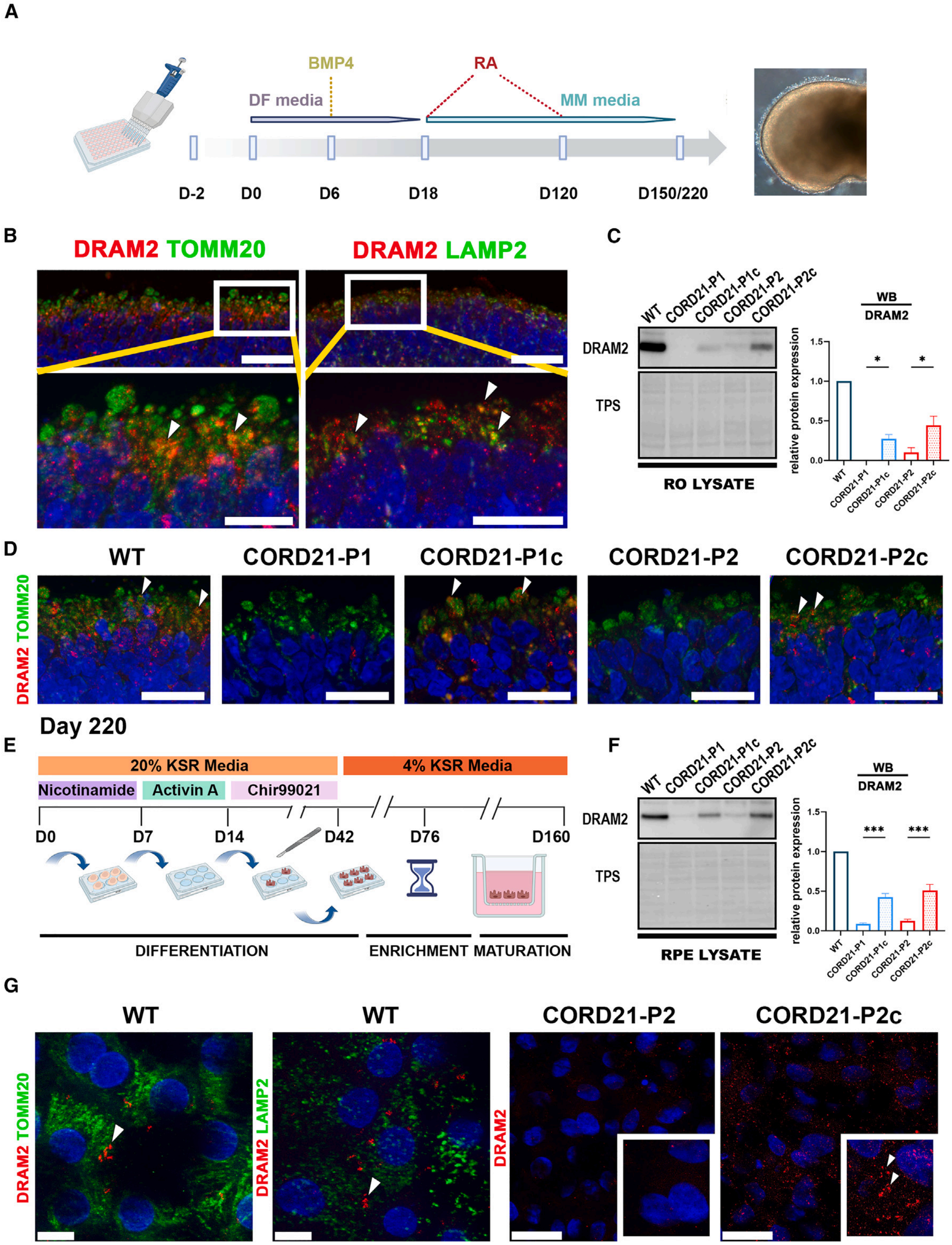

Figure 1. A significant reduction in DRAM2 expression is observed in CORD21-ROs and RPE cells
(A) Schematic diagram showing the RO differentiation procedure. DF-differentiation and MM-maintenance media.
(B) DRAM2 co-localizes with mitochondrial marker TOMM20 and lysosomal marker LAMP2 in the ISs of wild-type ROs (white arrowheads). These are representative images from 15 ROs imaged from three different differentiation experiments. Scale bars represent 20μm and 10μm in the top and bottom magnified panels, respectively.

(legend continued on next page)

photoreceptor inner segments (ISs) and the apical surface of retinal pigment epithelium (RPE) cells (El-Asrag et al., 2015). A recent single-cell RNA sequencing study has shown that *DRAM2* is ubiquitously expressed in the human eye, but the expression is low and comparable between the different retinal cell layers (Jones et al., 2023). Two human *DRAM2* isoforms (a and c) are ubiquitously expressed in all tissues, with the brain and retina displaying increased expression of isoform c compared to other tissues (Abad Morales et al., 2019).

To date, only one published study has investigated the function of DRAM2 in the context of retinal degeneration, showing significant loss of cone photoreceptors in *Dram2* knockout mice from 4 months of age. No difference in photopic or scotopic responses was detected in 21-month-old mice, suggesting that loss of Dram2 in mice leads to age-related degeneration, but the severity of the retinal phenotype does not impair visual function even in relatively old mice (Jones et al., 2023). To gain insights into the pathomechanisms underlining DRAM2-dependent cone-rod dystrophy, we generated iPSCs from two CORD21 patients and differentiated those to retinal organoids (ROs) and RPE cells. Both models showed loss of DRAM2 protein associated with abnormal lipid metabolism, autophagy flux defects, reduced lysosomal enzyme activity, and accumulation of aberrant lysosomal content.

RESULTS

DRAM2 expression is significantly reduced in CORD21-ROs and RPE cells

Dermal skin fibroblasts obtained from two CORD21 patients herein named as CORD21-P1 (homozygous for a loss-of-function variant at c.140delG) and CORD21-P2 (compound heterozygote for an intron-exon missense change c.131G>A and a nonsense frameshift variant c.494G>A, Table S1) were reprogrammed into iPSCs (Figure S1A). CORD21 iPSCs were free of Sendai transgenes and displayed the expression of pluripotency markers (Figures S1B and S1C). CRISPR-Cas9 *in situ* gene editing for one mutation (c.140delG in CORD21-P1 iPSCs and c.131G>A in CORD21-P2 iPSCs, Figures S2A and S2B) enabled the generation of heterozygous controls named herein as CORD21-P1c and CORD21-P2c (Figure S1D). CORD21 and corrected iPSCs were pluripotent and lacked genomic instabilities (Figures S1E–S1G) and off-target effects (Figures S2C and S2D).

CORD21 iPSCs and isogenic controls were differentiated to ROs (Figure 1A) and RPE cells (Figure 1E) alongside wild-type iPSCs (referred herein as WT) (Dorgau et al., 2022; Regent et al., 2019). Immunofluorescence (IF) analysis at day 220 of RO differentiation demonstrated the presence of all major retinal cell types in CORD21 and control-derived retinal tissues (Figures S3A–S3E). No changes in the abundance of cells immunostained with markers of all mature retinal cell types including cones and rods were observed between the CORD21- and control ROs (Figure S3F). Qualitative IF characterization indicated the preservation of apico-basal polarity and the presence of tight junction zonula occludens (ZO-1) staining in CORD21 and control RPE cells (Figures S4A and S4B). No changes were observed in the apical and basal secretion of pigment epithelium-derived factor (PEDF) and vascular endothelial growth factor (VEGF), respectively, or ability to phagocytose photoreceptor outer segments (POSs) between CORD21- and control RPE cells (Figure S4C).

To corroborate the specificity of DRAM2 protein detection by western blot (WB), we subjected day 220 WT ROs to *DRAM2*-small interfering RNA (siRNA) knockdown (Figure S5A). Approximately a 50% reduction in DRAM2 expression was confirmed by quantitative reverse-transcription PCR (RT-qPCR) and WB following 72 h of siRNA treatment (Figures S5B and S5C). IF analysis demonstrated punctate DRAM2 localization in the photoreceptor ISs specifically in the mitochondria and lysosomes marked by TOMM20 and LAMP2 immunostaining, respectively (Figure 1B). DRAM2 was localized to the mitochondria and lysosomes in RPE cells (Figure 1G). Complete loss of DRAM2 expression in CORD21-P1 and a significant DRAM2 reduction in CORD21-P2 ROs and RPE cells (Figures 1C and 1F)

(C) WB shows a significant reduction in DRAM2 expression in day 220 CORD21-ROs. Data are presented as mean +SEM (n = 3 different differentiation experiments each consisting of 48 ROs/sample), *p <0 .05.

(D) DRAM2 localizes to the ISs of wild-type and isogenic control (as indicated by white arrowheads) but is absent in CORD21-ROs. Scale bar, 20 μm. These are representative examples from 15 ROs imaged from three different differentiation experiments/sample.

(E) Schematic diagram of iPSC-directed differentiation to RPE cells.

(F) WB shows a significant reduction of DRAM2 protein abundance in CORD21-RPE cells. Conversely, DRAM2 is detected in the wild-type and the isogenic control ROs. Data are presented as mean +SEM (n = 3 different differentiation experiments each consisting of 2 wells of a 12-well plate of RPE cells/sample), ***p <0 .001.

(G) Co-localization of DRAM2 with TOMM20 and LAMP2 in RPE cells derived from WT iPSCs and absence of DRAM2 protein in CORD21-P2 RPE cells. A punctate pattern of protein expression can be seen in the CORD21-P2c isogenic control (white arrows). These are representative examples from 15 RPE transwells imaged from three different differentiation experiments. Scale bar, 20 μm.

were observed by WB; however, no DRAM2 expression was noted by IF in CORD21-ROs and RPE cells (Figures 1D and 1G).

Autophagy impairment and accumulation of lipopigments in CORD21-ROs

To evaluate the effect of *DRAM2* mutations on the state of autophagy flux in ROs, LC3 expression was evaluated following the sequential addition of 500 nM rapamycin for 24 h and/or 100nM bafilomycin over the course of the last 4 h of the treatment (Figure 2A). Untreated CORD21-P1 and -P2 ROs showed reduced conversion of LC3-I to LC3-II, as demonstrated by the retention of LC3 mainly in the form of LC3-I (Figure 2B). A complete loss of flux in CORD21-P1 ROs was indicated by the failure to accumulate LC3-II following single bafilomycin or rapamycin treatment in contrast to CORD21-P2 ROs, which showed a dramatic increase in LC3-II conversion (Figures 2B and 2C).

Transmission electron microscopy (TEM) revealed a significant accumulation of lysosome-like structures in the photoreceptors of CORD21-ROs (Figures 2D–2F), which were identified as curvilinear lipopigments (CLs) due to their strong resemblance to the granular lipopigments and curvilinear profiles associated with lipid deposition in various forms of neuronal ceroid lipofuscinoses (Anderson et al., 2013). No statistically significant changes in the abundance of lysosomes, the number of early (AVi) and late autophagic vehicles (AVd) (Figures 2E and 2F), and the number of mitochondria or gross alterations in mitochondrial shape between CORD21 and control photoreceptors (Figures S6A and S6B) were observed. The form factor was significantly higher in CORD21-P1 photoreceptors compared to controls, suggesting enhanced mitochondrial branching (Figures S6A and S6B).

CORD21-ROs show a consistent reduction in CTSD expression

Extensive WB screening of key lysosomal markers and transport receptors in RO and RPE lysates (Figures 3 and S7) revealed the likely hypoglycosylation of LAMP1 and LAMP2 in CORD21-P1 ROs (Nabi and Dennis, 1998) (Figures 3A and 3B). LAMP1 was likely hypoglycosylated in CORD21-P1 RPE cells, but no changes in LAMP2 expression were observed in either of the patient RPE cells (Figures S7A and S7B). A marked reduction was seen in the protein expression of endosomal sorting marker CD63 and lysosomal enzyme glucocerebrosidase (GBA) in the lysates of CORD21-P1 and CORD21-P2 ROs, respectively (Figures 3A and 3B). No changes in the expression of early and late endosomal markers RAB5 and RAB7 or the lysosomal enzyme cathepsin B (CTSB) were observed between CORD21- and control ROs (Figures 3A and 3B). A key finding was the significant depletion of lysosomal aspartyl protease cathepsin D (CTSD) heavy chain in CORD21-ROs (Figures 3A and 3B) and CORD21-P1 RPE lysates (Figures S7A and S7B). This was not accompanied by enhanced secretion in the media of any CTSD form for either ROs (Figures 3C and 3D) or RPE cells (data not shown). The GBA receptor LIMP2 was downregulated and hypoglycosylated in CORD21-ROs; however, no significant changes in the expression of CI-M6PR (cation-independent mannose 6-phosphate receptor), CD-M6PR (cation-dependent mannose 6-phosphate receptor), or M6PR-independent transport proteins, sortilin and VPS35, were noted (Figures S7C and S7D).

We performed mass spectrometry protein analysis of both ROs and RPE cells generated in this study (Figures 4 and 5, Table S2). A total of 4,559 and 3,321 proteins were identified in RPE cells and ROs, respectively (Figures 4B and 5B). Out of the 101 commonly changed proteins in ROs, 19 followed the same tendency for up- or downregulation (Figures 4C–4E, and; Table S2), revealing a common deficiency of CTSD, PPT1, and NPC2 lysosomal proteins in both CORD21-ROs (Table S2). The significant depletion of CTSD and PPT1 was corroborated by RPE proteomics for both patients (Figures 5C–5E, and Table S2). Metascape enrichment analyses in both retinal models outlined vesicle-mediated transport/response as a major affected biological process, whereas mitochondrial respiration (respiratory chain complex I) was shown to be specifically affected in the RPE cells (Figures 4E and 5E).

Following the identification of PPT1 and NPC2 by liquid chromatography-tandem mass spectrometry (LC-MS/MS) as key downregulated proteins, we validated the intracellular PPT1 and NPC2 deficiency (Figures 4F, 4G, 5F, and 5G) and the concomitant accumulation of PPT1 and NPC2 in the media of CORD21-ROs and RPE cells by WB (Figures 4F, 4G, 5F, and 5G). Further, CORD21-ROS and RPE cell lysates were characterized by statistically significantly reduced enzymatic activities for lysosomal degradation enzymes CTSD, GBA, and α-Mannosidase (Figures 4H and 5H).

Altered membrane-lipid composition and aberrant accumulation of lipids in CORD21-ROs and RPE cells

To ascertain whether lipid imbalance is associated with the observed abnormalities in crucial lysosomal proteins, CORD21-ROs and isogenic controls were subjected to lipidomics analysis. CORD21-ROs were significantly depleted in phosphatidylethanolamine (PE), phosphatidylserine (PS), phosphatidylcholine (PC), phosphatidylinositol (PI), and phosphatidylglycerol (PG) glycerophospholipids

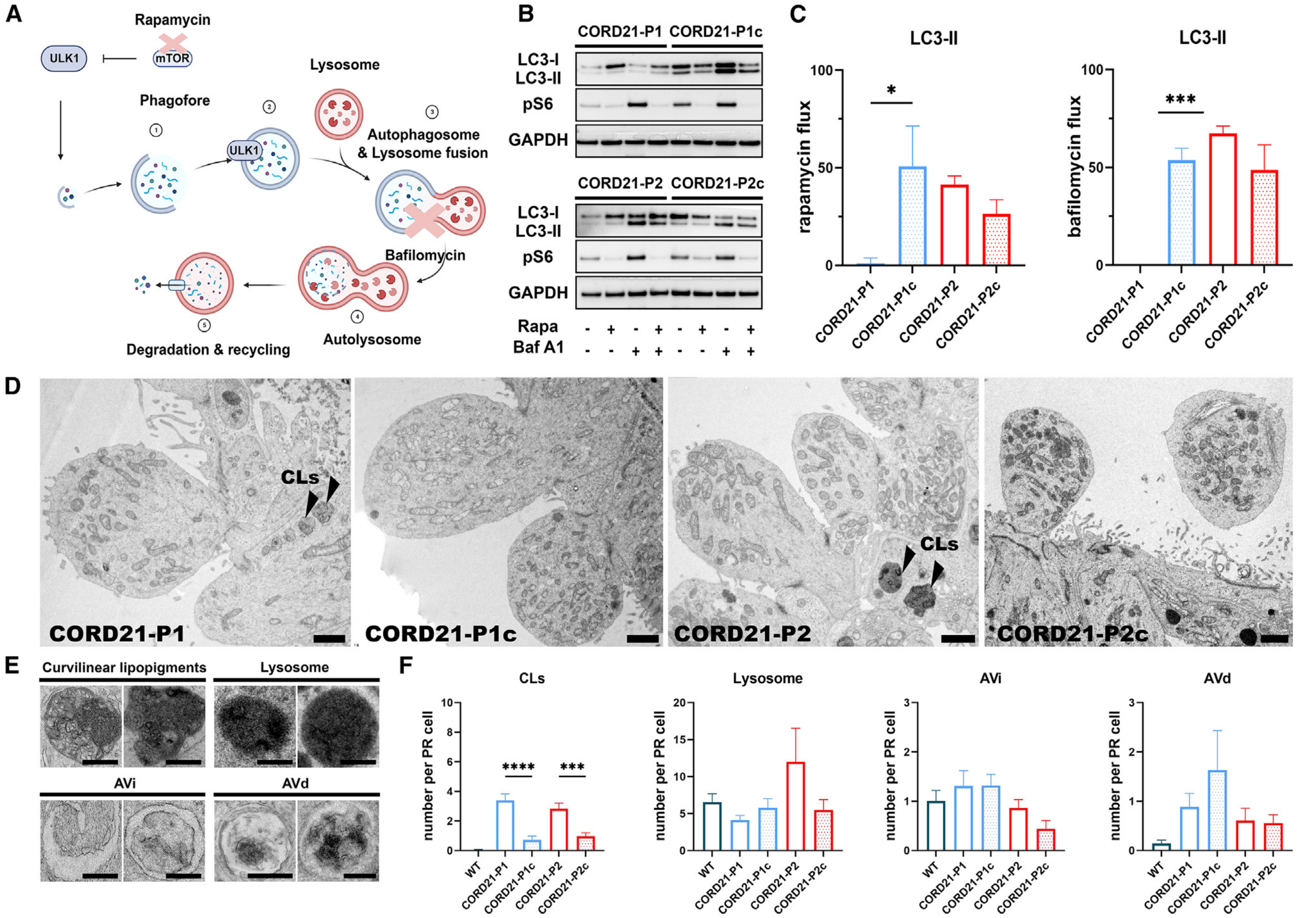

Figure 2. CORD21-ROs present with an autophagy impairment and the accumulation of CLs on TEM

(A) Diagram summarizes the effect of rapamycin and bafilomycin drugs on the autophagy pathway. Rapamycin, an mTOR inhibitor, relieves ULK1 from inhibition by mTOR. This leads to the assembly of phagophore double membrane around autophagic cargo and its subsequent maturation to a nascent autophagosome. Bafilomycin blocks the fusion of autophagosomes with lysosomes thereby preventing the formation of autolysosomes. LC3-II is attached to the double membrane of the autophagosome and serves as a marker for autophagosome turnover and degradation. An increase in pS6 expression suggested an inhibition of autophagy following bafilomycin treatment. Schematic was generated using BioRender.

(B and C) CORD21-P1 day 150 ROs show reduced rates of autophagic flux compared to isogenic control as apparent by the failure to accumulate LC3-II following single bafilomycin or rapamycin treatment. Data were normalized to GAPDH expression and presented as mean +SEM (n = 3 different differentiation experiments each consisting of 48 ROs/sample).

(D) CORD21-ROs display increased numbers of CLs (black arrowheads). By contrast no such features were present in the isogenic controls. Scale bar, 1 μm. These are representative examples from 10 ROs imaged from three different differentiation experiments/sample.

(E) Magnified images of quantified cellular structures (CL, scale bar, 500 nm; Lysosomes, scale bar, 125 nm; AVi, scale bar, 1.5 μm; 250 nm; AVd, scale bar, 500 nm, 250 nm).

(F) Bar charts showing an increase in CLs per photoreceptor in CORD21-ROs, without statistically significant changes in the numbers of lysosomes, early (AVi) and late autophagic vehicles (AVds). Data plotted as mean +SEM, assessed for normality and analyzed by non-parametric Kruskal-Wallis test (n = 10 ROs from three different differentiation experiments/sample), *p <0 .05***p <0 .001, ****p < 0.0001.

when compared to isogenic controls (Figure 6A). Consistent with a sphingolipid homeostasis defect, CORD21-ROs presented with an increase in monosialodihexosylgangliosides (GM3). A specific finding from the positive ion mode analysis was the accumulation of the isoprenoid alcohol dolichol (Figure 6A). Further, the lipidomic analysis revealed the enrichment of ceramide species in CORD21-ROs (Figure 6A). The buildup of ceramide in CORD21-ROs was shown to occur close to the area of photoreceptor cell bodies as established by IF analysis (Figure 6B) in contrast to control ROs

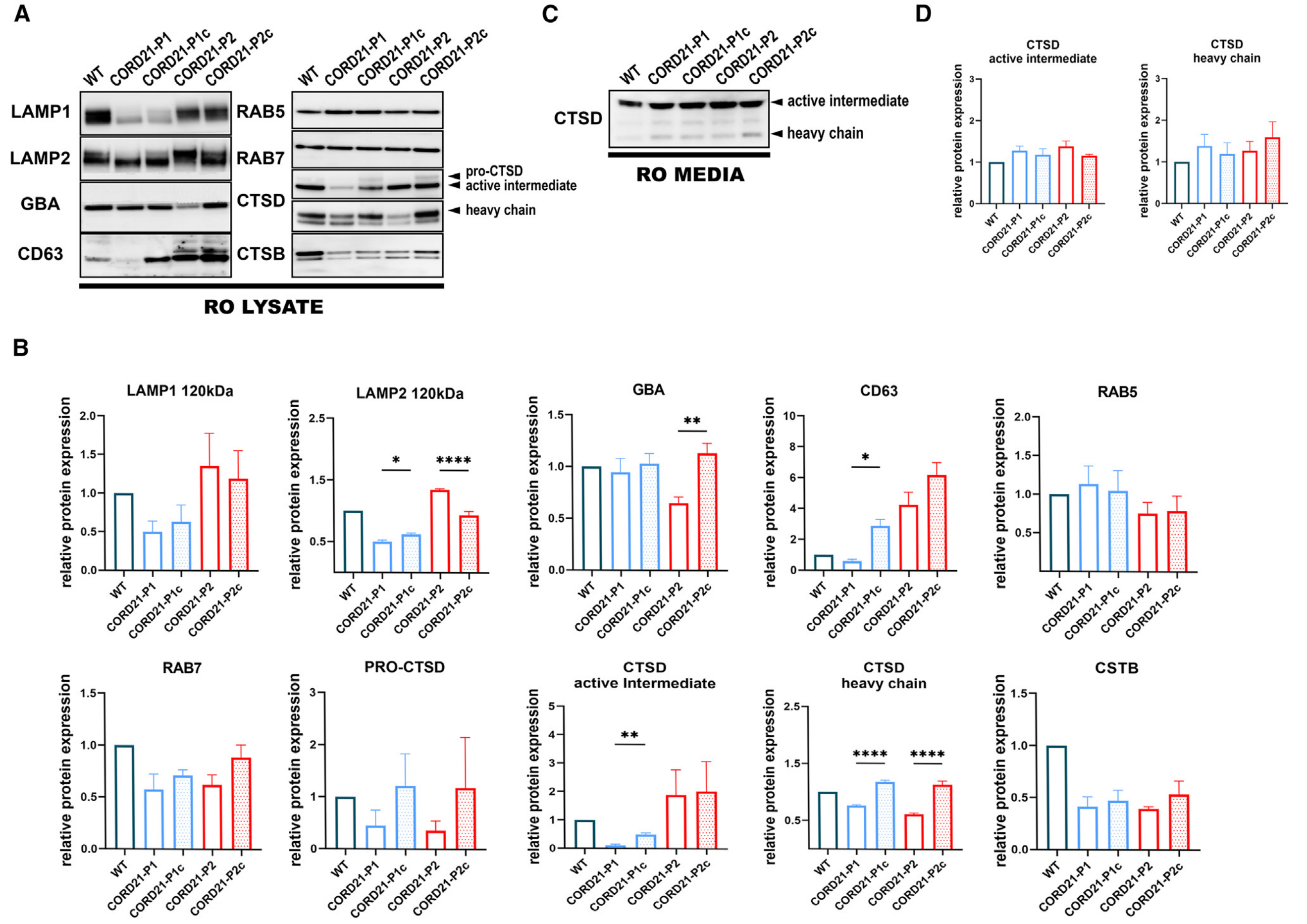

Figure 3. CTSD expression is reduced in day 220 CORD21-ROs lysates

(A and B) WB panel followed by quantitative analyses shows a significant CTSD heavy chain downregulation in CORD21-P1 and -P2 ROs. CD63 and GBA protein expression is significantly reduced in CORD21-P1 and -P2 ROs, respectively.

(C and D) Secreted CTSD heavy chain and active intermediate show no changes in expression levels between CORD21- and control ROs. (B and D) All data were presented as mean +SEM and normalized to the WT sample (n = 3–5 different differentiation experiments each consisting of 48 ROs/sample), $*p < 0.05$, $**p < 0.01$, $****p < 0.0001$.

where ceramide was found to co-localize with the IS area of photoreceptor cells, where DRAM2 is also abundant (Figure 6C). Ceramide accumulation was further corroborated in CORD21- RPE cells (Figure S4D).

TEM analysis of CORD21-RPE cells did not show any detectable ultrastructural abnormalities, particularly those associated with lysosomal content accumulation (data not shown); hence we assessed their ability to respond to POS-induced metabolic stress. The POS treatment revealed a variety of aberrant ultrastructural findings in CORD21-P1 POS (+) RPE (Figures 6D and 6E), including the accumulation of lipid-containing organelles (LCOs), lamellar bodies (LBs), and stage II mitochondria in the CORD21-P1 POS (+) sample relative to CORD21-P1 POS (−) and -P1c POS (+) RPE cells. TEM images from the CORD21-P2/P2c comparisons did not show any significant changes, corroborating the more severe phenotype observed in CORD21-P1 ROs and RPE cells throughout this study.

DRAM2 deficiency in CORD21-ROs and implications for clathrin-mediated transport

Proteomics analyses demonstrated downregulation of multiple subunits of the clathrin adaptors AP-1, AP-2, and AP-3 and vesicle-mediated transport/response as a major affected biological process in both CORD21-ROs and RPE cells (Table S2).Thus to ascertain a putative vesicular trafficking defect, we assessed the expression of major subunits of the clathrin adaptors, AP-1γ, AP-2α, and AP-3β in the lysates of ROs and RPE cells, revealing a significant downregulation of AP-1γ in CORD21-P1 ROs and of AP-3β in CORD21-P2 RPE relative to isogenic controls

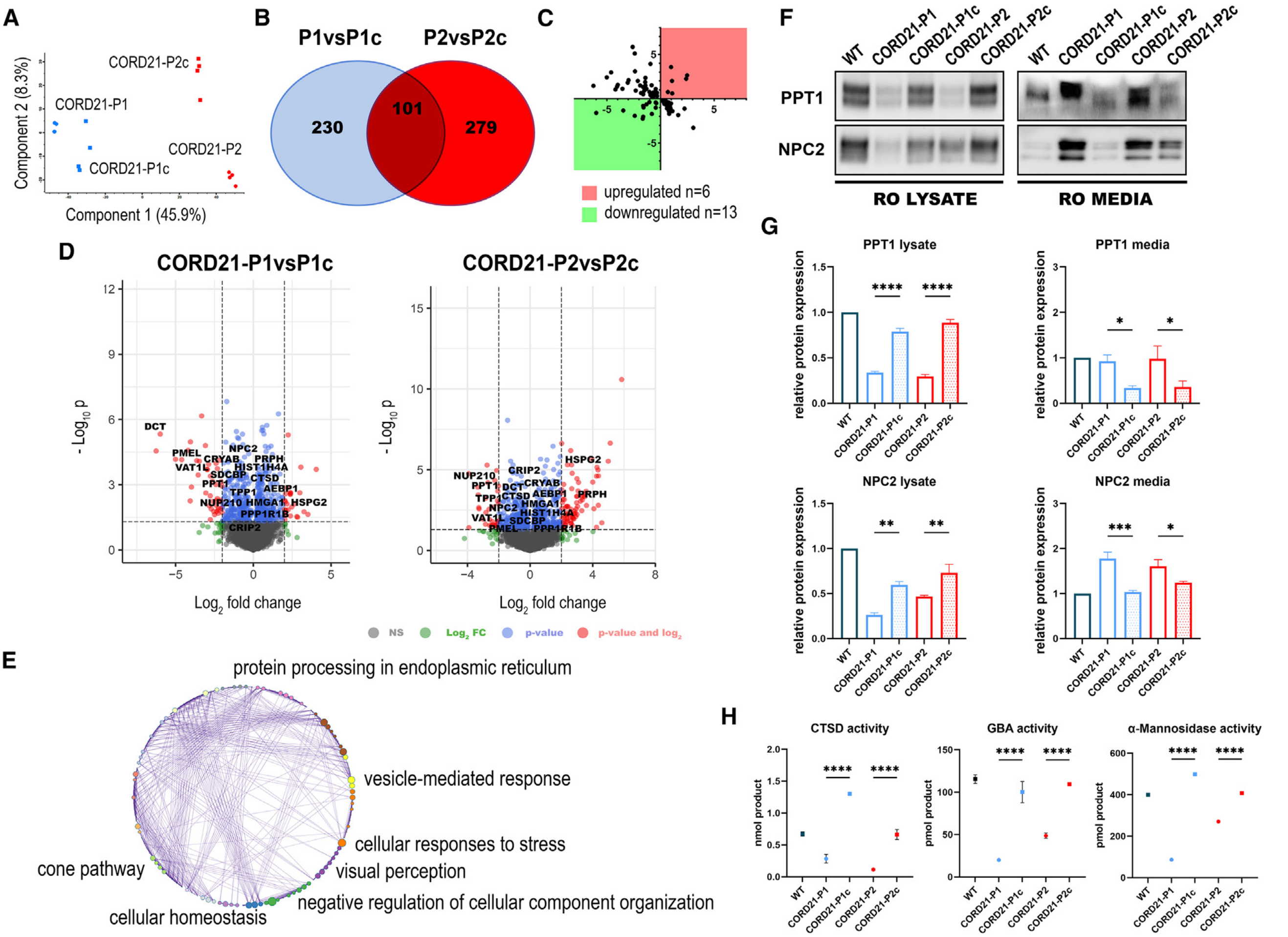

Figure 4. Differential protein analysis identifies vesicle-mediated response as a key biological process in CORD21-ROs

(A) Principal-component analysis reveals clear separation between CORD21-P1/P1c (outlined in blue) and CORD21-P2/P2c ROs (red).

(B) Venn diagram shows an overlap of 101 commonly changed proteins following Tuckey's *post hoc* test (false discovery rate [FDR]<0.05) (n = 3–4 different differentiation experiments each consisting of 48 ROs/sample).

(C) Dot plot highlighting commonly upregulated (red) and downregulated (green) proteins (n = 19).

(D) Volcano plots enable visual identification of tandemly changed proteins with logged fold change cutoff >2 that are also statistically significant (logged $p < 0.05$) (n = 19).

(E) Gene Ontology (GO) term enrichment of significantly changed proteins following the same trend by Metascape revealed major affected biological processes.

(F and G) PPT1 and NPC2 enzymes are severely deficient in the lysates of CORD21-P1 and CORD21-P2-ROs relative to controls. This deficiency associates with PPT1 and NPC2 hypersecretion to the extracellular media of matched CORD21-ROs. A total protein stain was used to confirm equal protein loading, and normalization was conducted to the WT sample. Data are presented as mean +SEM, n = 3 different differentiation experiments each consisting of 48 ROs/sample.

(H) Kinetic assay for the activity of CTSD shows reduced enzymatic activity in CORD21-ROs lysates relative to isogenic control (ANOVA). Endpoint enzymatic activity assays for GBA (ANOVA) and α-Mannosidase (ANOVA) demonstrated a similar reduction in enzymatic activities in day 220 CORD21-ROS. Data were shown as mean +SEM (n = 3 different differentiation experiments each consisting of 48 ROs/sample).

(Figures 7A and 7B). The GARP (Golgi-associated retrograde protein) component VPS53 was found to be specifically downregulated in CORD21-P2 ROs lysate (Figure 7A). IF experiments in WT ROs demonstrated partial co-localization of DRAM2 with clathrin and a stronger co-staining with AP-1 and AP-3 (Figure 7C).

DISCUSSION

CORD21 retinopathy is a form of recessive cone-rod dystrophy, typically presenting with a sight impairment, photoreceptor loss, and macular atrophy between the third and the sixth decades of life. A recent study using a *Dram2*

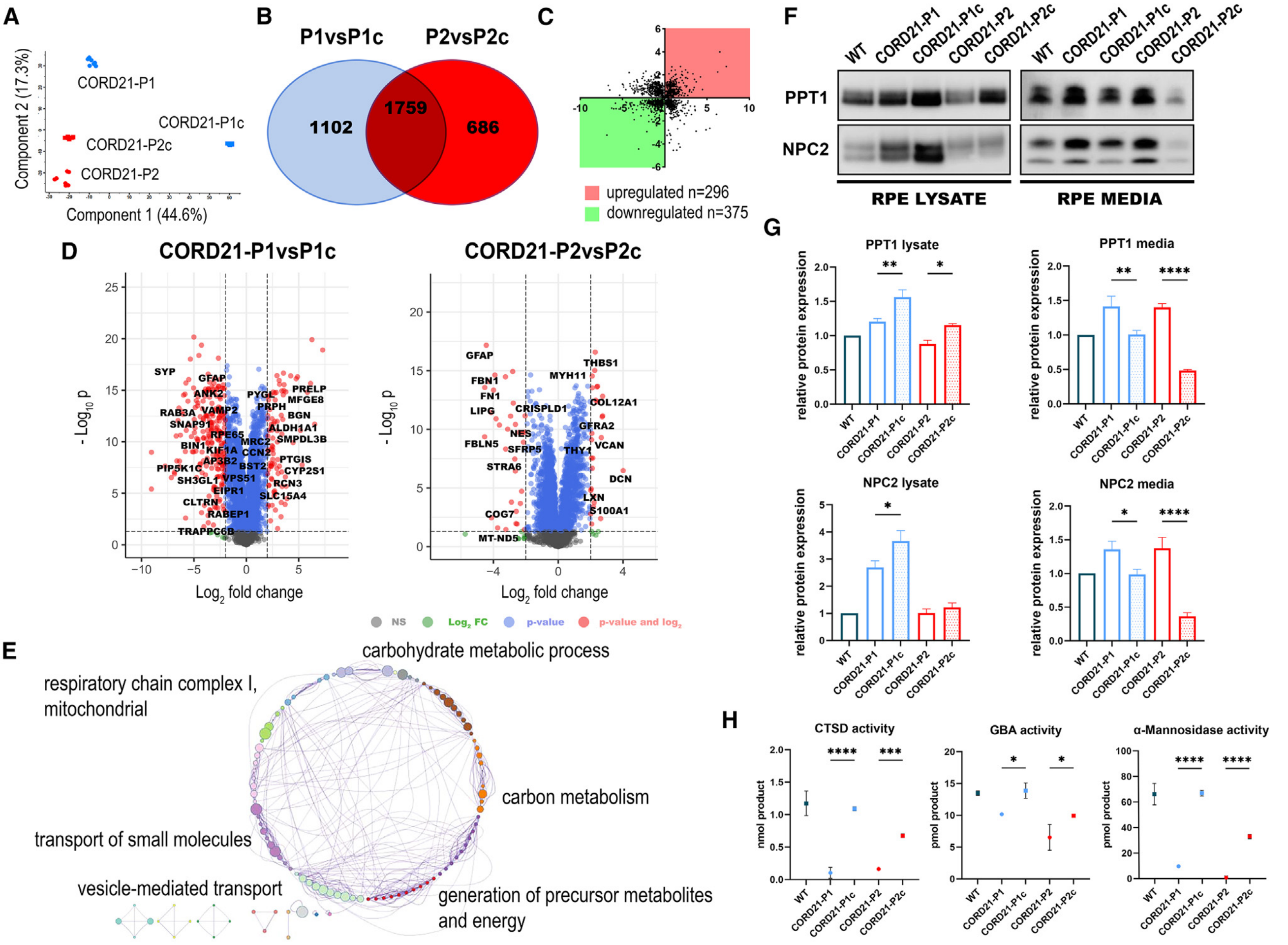

Figure 5. RPE proteome analysis of CORD21-RPE cells identifies changes in key proteins linked to vesicular-mediated transport

(A) Principal-component analysis (PCA) showed a distinct separation between CORD21-P1/P1c (blue) and CORD21-P2/P2c RPE cells (red).
(B) Venn diagram illustrates the 1,759 proteins that are commonly changed between P1/P1c and P2/P2c RPE cells (Tukey's *post hoc*, FDR<0.05, $n = 7$ different differentiation experiments each consisting of 2 wells of a 12-well plate of RPE cells/sample).
(C) Dot plot revealed a total of 296 (red) and 375 (green) proteins that are up- and downregulated, respectively.
(D) Volcano plots show key targets involved in cellular transport in both comparison groups with logged FC > 2 that represent statistically significant changes ($p < 0.05$) (P1/P1c, $n = 32$) (P2/P2c, $n = 20$).
(E) GO enrichment analysis of RPE differentially expressed proteins conducted using Metascape identified respiratory chain complex I, carbohydrate metabolic process, carbon metabolism, generation of precursor metabolites and energy, transport of small molecules, and vesicle-mediated transport as affected biological processes.
(F and G) WB data showed PPT1 and NPC2 RPE intracellular deficiency is likely due to aberrant secretion to the extracellular media. Equal protein loading was visualized by the total protein stain, and data were normalized to the WT sample. Data are shown as mean +SEM (n = 3–4 different differentiation experiments each consisting of 6 wells of a 12-well plate of RPE cells/sample).
(H) Reduced CTSD enzymatic activity in CORD21-RPE lysates relative to isogenic controls. Endpoint activity assays for GBA and α-Mannosidase demonstrated a similar enzymatic reduction in CORD21-RPE cell lysates. Data are shown as mean +SEM ($n = 3$ different differentiation experiments each consisting of 2 wells of a 12-well plate of RPE cells/sample).

knockout mouse model has shown age-related retinal degeneration but no visual dysfunction in relatively old mice (Jones et al., 2023). The difference between the clinical phenotypes of CORD21 cone-rod dystrophy and the *Dram2* knockout mouse model may be due to fundamental differences in retinal structure between mice and humans as well as the short lifespan in mouse, which does not allow modeling of the progressive age-related changes in man (Volland et al., 2015). To gain insights into the pathomechanism of the disease, we generated ROs and RPE cells from two human CORD21 patients, heterozygous isogenic and unaffected iPSC controls. Our data demonstrate a key

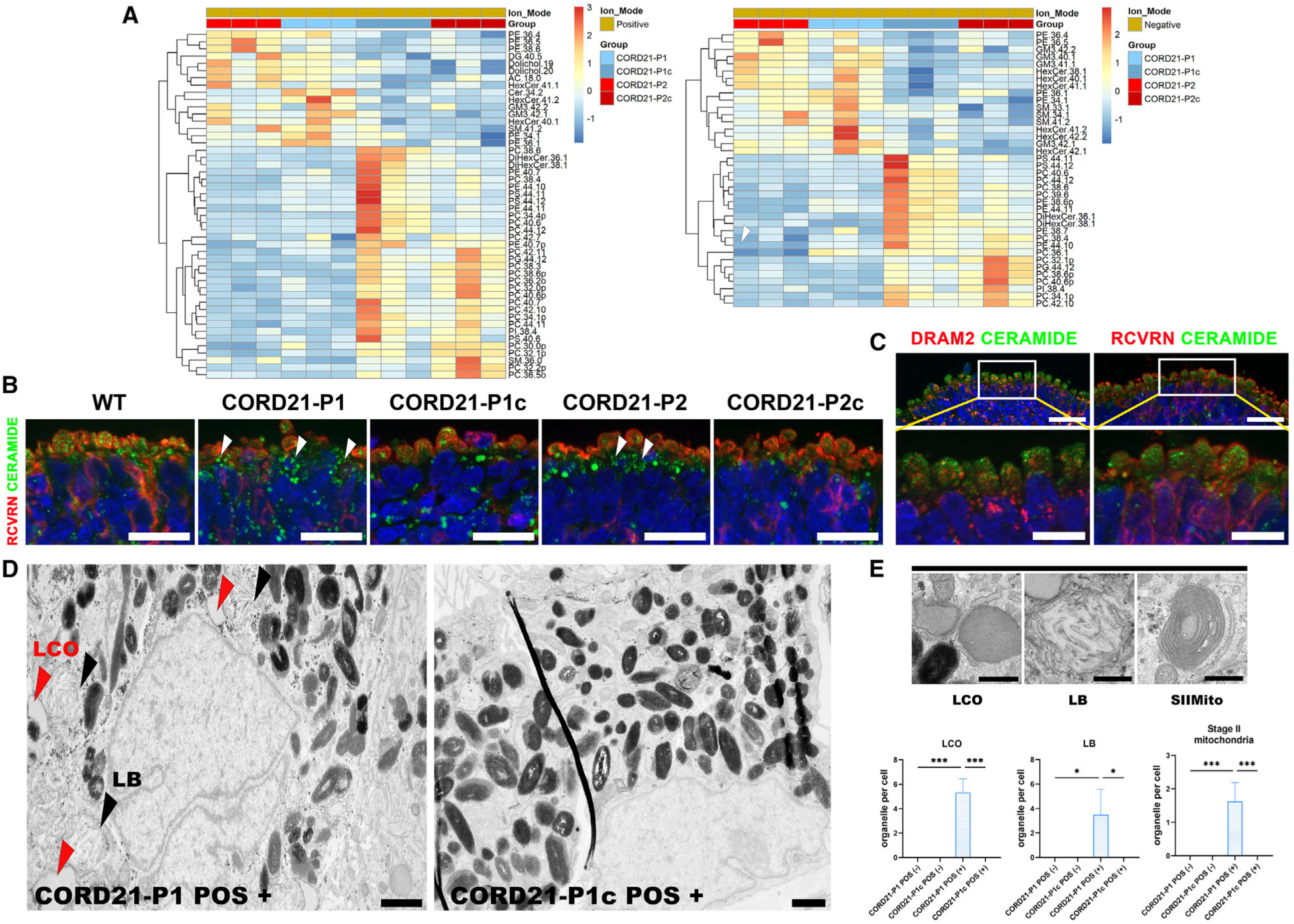

Figure 6. Lipidomic analysis of CORD21 and isogenic control ROs

(A) Heatmaps demonstrate statistically significant changes in lipid species under positive and negative ion modes of data acquisition based on t test and p value <0.05 cutoff (n = 4 different differentiation experiments each consisting of 48 ROs/sample).

(B) Recoverin (RCVRN) and ceramide double staining in day 220 ROs shows ceramide accumulation basally to photoreceptor ISs. In WT and isogenic controls, ceramide expression is mostly limited to the IS of the photoreceptor layer indicated by RCVRN staining. By contrast, in CORD21-Ros, ceramide accumulation extends beyond the ISs into the photoreceptor cell bodies, and further basally into the reaches of secondary neurons at the apical edge. These are representative examples from 15 ROs imaged from three different differentiation experiments. Scale bars represent 20 μm.

(C) Ceramide expression (green) pertains to the RCVRN+ photoreceptor layer (red, right image) where ceramide is co-expressed with DRAM2 within the IS region of wild-type ROs (red, left image). These are representative examples from 15 ROs imaged from three different differentiation experiments/sample. Scale bars top images 20 μm; bottom magnified images scale bars 10 μm.

(D) POS treatment leads to the accumulation of lipid-containing organelles (LCOs, red arrowheads) and lamellar bodies (LBs, black arrowheads) in CORD21-P1 POS (+) relative to untreated CORD21-P1 POS (−) or CORD21-P1c POS (+) RPE cells (scale bar, 1 μm).

(E) TEM analysis showed accumulation of LCOs, stage II abnormal mitochondria, and LBs in POS-treated CORD21-P1 RPE cells. LCO (scale bar, 0.5 μm), LBs (scale bar, 0.25 μm). Stage II aberrant mitochondria (SIIMito) (scale bar, 0.5 μm). Plots show the significant accumulation of LCOs, SIIMito, and LBs in CORD21-P1 POS (+) relative to CORD21-P1 POS (−) and CORD21-P1c POS (+) RPE cells (Kruskal-Wallis test). Data are presented as mean +SEM (n = 10–12 images of RPE cells from three different differentiation experiments/sample). Statistical comparisons for CORD21-P1 POS (−) vs. CORD21-P1 POS (+), CORD21-P1c POS (−) vs. CORD21-P1c POS (+) and CORD21-P1 POS (−) vs. CORD21-P1c POS (+) are denoted by *p <0 .05, **p <0 .01, ***p <0 .001.

lysosomal deficiency in CTSD, NPC2, and PPT1 enzymes in CORD21-ROs and RPE cells, associated with reduced lysosomal enzyme activity, impaired autophagic flux, abnormal lipid metabolism, and aberrant lysosomal content accumulation. Based on recent studies on mir144* in human monocytes, loss of DRAM2 expression in CORD21-ROs and RPE could lead to decreased autophagy (Kim et al., 2017), in line with our data demonstrating

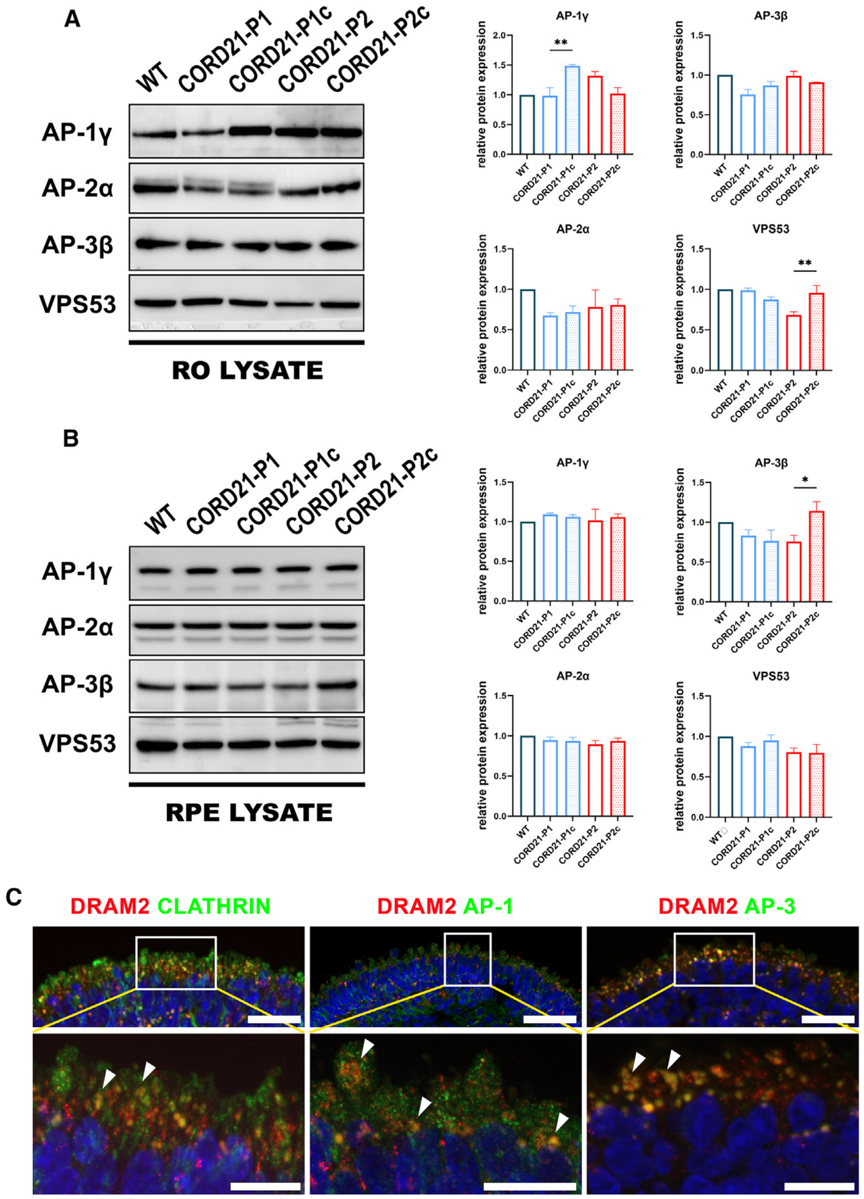

Figure 7. DRAM2 co-localizes with clathrin vesicle adaptors AP-1 and AP-3 and affects their expression

(A) A significant reduction in protein expression was established for GARP component VPS53 and AP-1γ adaptor for CORD21-P2 and -P1 RO lysates, respectively. Scale bars represent 20 μm. Data are presented as mean +SEM (n = 3–4 different differentiation experiments each consisting of 48 ROs/sample) and normalized to the WT sample.

(B) RPE cell analysis by WB demonstrated a significant downregulation of clathrin transporter AP-3β in the lysates of CORD21-P2 RPE cells relative to isogenic controls. Total protein stain was used to visualize equal protein loading, and data were normalized to the WT sample. Scale bars represent 20 μm.Data are shown as mean +SEM (n = 3–4 different differentiation experiments each consisting of 2 wells of a 12-well plate of RPE cells/sample). Statistical comparisons for CORD21-P1vs -P1c and CORD21-P2vs-P2c are denoted by **p* <0 .05, ***p* <0 .01.

(C) Clathrin (green) is detected in a dotty-like pattern across the entirety of the photoreceptor IS and can be seen to only partially co-stain with DRAM2 (red, left image). DRAM2 expression (red) strongly overlaps with that of transport vesicle proteins AP-1 (green, middle) and even more so with AP-3 (green, right) at the IS of ROs. Hoechst (blue) counterstains nuclei; white arrowheads show DRAM2 (red) co-localization with clathrin, AP-1, and AP-3 markers (green). These are representative examples from 15 ROs imaged from three different differentiation experiments. Scale bars represent 20 μm. Top panels scale bar, 20 μm; bottom panel scale bar, 10 μm.

the requirement of DRAM2 in the conversion of LC3-II. Together our data suggest a key role for DRAM2 in lysosomal and autophagic function in the retinal cells.

TEM and WB findings for CORD21-P1 ROs and RPE (c.140delG) correlated with a more exacerbated phenotype consistent with an early onset (22 years) and rapid disease progression observed in the CORD21-P1 patient (Table S1) (Sergouniotis et al., 2015; El-Asrag et al., 2015). Conversely, the CORD21-P2 patient (c. 131G>A, c.494 G>A), initially diagnosed at the age of 29, developed central macular atrophy characterized by severely attenuated electroretinograms at the age of 47 (Table S1). The slow-progressing DRAM2 phenotype in CORD21-P2 ROs and RPE is in line with residual DRAM2 protein expression which could be detected by WB (~30 kDa). Even though c.131G>A is predicted to impact mRNA splicing, it does not affect the retinal-specific *DRAM2c* isoform, underscoring the significance of *DRAM2a* as a disease-causing isoform in the development of CORD21-P1 and -P2 retinal phenotypes (El-Asrag et al., 2015).

We observed the accumulation of aberrant lysosomal structures in CORD21-ROs, a significant depletion of key lysosomal enzymes, namely CTSD, PPT1, and NPC2, and a significant reduction in CTSD activity. CTSD deficiency

is associated with mutations in the *CLN10* gene which cause *CLN10* disease, affecting visual and neuromotor development (Williams and Mole, 2012; Schulz et al., 2013). Indeed, DRAM2 deficiency reported here recapitulates *Cln10* mouse phenotypes as inferred by the decreased CTSD activity, the early presentation of lipopigments, and the associated autophagic impairment in retinal cells. The predominant cone phenotype preceding the loss of rods in *Cln10*$^{-/-}$mice (Bassal et al., 2021) is consistent with the classical presentation of CORD21 macular dystrophy (El-Asrag et al., 2015; Sergouniotis et al., 2015; Abad-Morales et al., 2019; Bassal et al., 2021); however, the CORD21-ROs did not reveal changes in the expression of mature cone and rod markers in agreement with the adult onset of CORD21 clinical phenotype (Krašovec et al., 2022).

In contrast to CTSD, NPC2 expression is significantly downregulated in the cytosol, but increased in the media, which could be due to impaired vesicular trafficking. NPC2 is a lysosomal lumen protein which sequesters free cholesterol and removes it from lysosomes (Infante et al., 2008; Kwon et al., 2009). *NPC2* mutations cause a cholesterol and sphingolipid storage disorder known as Niemann-Pick disease type C (NPC), which has severe implications for cognitive, liver, and spleen function (Ribeiro et al., 2001; Park et al., 2003; Vanier and Millat, 2003; Pentchev, 2004; Chang et al., 2005; Evans and Hendriksz, 2017). Disease phenotype arises from the inability of NPC mutants to downregulate cholesterol biosynthesis through the generation of oxysterols (Frolov et al., 2003). As cholesterol is an indispensable component of phospholipid bilayers, its aberrant distribution across cell membranes is likely to perturb the dynamics of cell trafficking (Puri et al., 1999; Radhakrishnan et al., 2008). Our lipidomics analysis conducted on CORD21-ROs and controls did not demonstrate the accumulation of cholesterol or ergosterol esters as outlined by Frohlich et al. (2015) but showed an accumulation of GM3 ganglioside and hexosylceramide species in CORD21-ROs, consistent with the neuronal phenotype of NPC2 deficiency (Vanier, 1999). The lysosomal accumulation of sphingolipids may incur toxicity by inhibiting glycerolipid synthesis (Wu et al., 1993; Contreras et al., 2006) and cause profound defects in lysosomal trafficking (Lloyd-Evans et al., 2008). Indeed, we observed increased levels of ceramide in both CORD21-ROs and RPE cells. Aberrant levels of ceramide have been shown to be toxic to retinal ganglion cells (Fan et al., 2021), photoreceptor (German et al., 2006; Chen et al., 2013), and RPE cells (Levitsky et al., 2020), providing a likely explanation for the retinal toxicity associated with CORD21 retinal dystrophy. Furthermore, an underlying sphingolipid defect is also in agreement with the decrease of GBA enzymatic activity in the lysates of both CORD21 retinal cell models.

An interesting finding from our lipidomics data was the increase of dolichol species in CORD21-ROs. Dolichol, a compound found in ceroid lipofuscin pigments, is implicated in the aging process and various lipid storage disorders (Leloir, 1977). A phosphorylated form of dolichol is involved in N-linked glycosylation of proteins (Rip et al., 1983). Congruent with this, we observed that lysosomal receptors LIMP2, LAMP1, and LAMP2 are likely hypoglycosylated in CORD21-P1 ROs, while no major changes were observed in the glycosylation pattern of CORD21-P2 ROs. Notably, LAMP1 is also likely hypoglycosylated in CORD21-P1 RPE cells (Figure S7A), suggesting that protein glycosylation abnormalities are typical only for CORD21-P1 retinal cells. The complete absence of DRAM2 expression in CORD21-P1 retinal cells, as noted previously, could be the underlying cause. This deficiency may be associated with glycosylation abnormalities occurring upstream in the cellular pathway, possibly within the endoplasmic reticulum (ER) or Golgi apparatus. These abnormalities could disrupt the normal metabolism of lysosomal membranes, which are rich in dolichol. Consequently, this disrupted metabolism could lead to the accumulation of dolichol within the cells.

Similar to NPC2, the cytosolic PPT1 deficiency was associated with extracellular release in CORD21-ROs and RPE cells. PPT1, which exhibits high enzymatic activity in the brain and the retina, is known to facilitate the lysosomal breakdown of lipidated proteins (Chattopadhyay and Pearce, 2000; Dearborn et al., 2016). PPT1 is encoded by *CLN1*, and mutations in this gene are associated with a severe neurodegenerative disorder known as infantile neuronal ceroid lipofuscinosis (INCL, also known as Batten disease) (Santavuori et al., 1973; Weleber, 1998, 2004; Jalanko and Braulke, 2009). Visual deterioration presents as one of the earliest signs of *CLN1* disease and is accompanied by the accumulation of ceroid in the retina (Santavuori, 1988), photoreceptor degeneration, and impairment of second-order neurons and retinal ganglion cells (Weleber et al., 2004). PPT1 has been shown to regulate lysosomal acidification via the lysosomal targeting of the V0a1 vacuolar ATPase (vATPase) (Bagh et al., 2017), and altered lysosomal pH underlies many lysosomal storage disorders (Hu et al., 2015). Hence, the downregulation of PPT1 in both CORD21-ROs and RPE cells could contribute to lysosomal malfunction, leading to the accumulation of CLs.

Comparative proteomic analyses indicated the involvement of DRAM2 in vesicular trafficking, as inferred from the downregulation of multiple AP-1 and AP-3 subunits observed in CORD21-ROs and RPE cells. These findings, combined with DRAM2 deficiency leading to reduced levels of critical lysosomal proteins including CTSD, NPC2, PPT1, MAN2B1 (Mannosidase alpha class 2B member 1), and GBA in ROs and RPE cells, suggest potential

vesicular mis-trafficking within the clathrin pathway, resulting in abnormal delivery of lysosomal resident proteins. The identified vesicular pathways implicated in the depletion of these essential lysosomal proteins likely converge on the regulation of lysosomal trafficking by clathrin adaptors AP-1 and AP-3 (Berger et al., 2007), despite discrepancies observed for AP-1 and AP-3 in our WB analyses. Notably, co-localization of DRAM2 with AP-1 and AP-3 in WT ROs further supports a role for DRAM2 in lysosomal function and the regulation of LC3-II conversion. Further experimental work is necessary to definitively establish the regulation of DRAM2 via clathrin-mediated transport, such as co-immunoprecipitation studies with AP-1 and AP-3. Future studies could also benefit from determining the precise subcellular localization of DRAM2 within the endolysosomal and vesicular transport systems.

In summary, our study provides novel insights into the cellular effects of DRAM2 deficiency in photoreceptors and RPE cells, contributing to our understanding of biological mechanisms underlying retinal degeneration.

EXPERIMENTAL PROCEDURES

Resource availability

Lead contact

Further information and requests for resources and reagents should be directed to and will be fulfilled by the lead contact, Majlinda Lako (majlinda.lako@ncl.ac.uk).

Materials availability

iPSCs generated in this study are available from the lead contact with a completed Materials Transfer Agreement.

Data and code availability

The proteomics data have been submitted into MassIVE: MSV000094178.

Ethics permission

Dermal skin fibroblasts were isolated from individuals diagnosed with CORD21 retinal dystrophy following acquisition of informed written consent in accordance with the Yorkshire and the Humber Research Ethics Committee (REC ref. no. 15/YH/0365).

Statistical analyses

Data were assessed for normality using a Shapiro-Wilk's test. Assuming a normal distribution, the data points across different groups were compared using a parametric one-way ANOVA test (Šídák's multiple comparisons test). When the data did not meet the normality criteria, a non-parametric Kruskal-Wallis test was applied. Comparisons were performed in pre-selected pairs (CORD21-P1 vs. P1c and CORD21-P2 vs. P2c), whereby the WT control was omitted from the statistical analysis. Statistical analysis was conducted using GraphPad Prism version 9.5.0. All data were presented as mean +SEM values, and statistical significance was assumed when $p \leq 0.05$ ($*p \leq 0.05$, $**p \leq 0.01$, $***p \leq 0.001$, $****p \leq 0.0001$).

For details on various assays performed during the course of this study, please refer to the supplemental information.

SUPPLEMENTAL INFORMATION

Supplemental information can be found online at https://doi.org/10.1016/j.stemcr.2024.06.002.

ACKNOWLEDGMENTS

The authors are grateful to Macular Society UK and EPSRC (EP/Y031016/1) for funding this work, Rachel Wilson for contributing to the derivation of CORD21-P2 iPSCs, Dan Singleton for contributing to TEM analyses, and BSc students Gabrielle Chai Jia Min and Sam Steel for performing pluripotency and genomic stability tests in CORD21-P1 and P1C iPSCs.

AUTHOR CONTRIBUTIONS

R.T. performed experiments, data collection and analyses, and manuscript and figure preparation. E.G., E.C., D.H., M.G., R.C.-C., R.A., P.P., F.B., T.D., J.H., and P.W. performed experiments, data collection and analyses, and figure preparation. D.H.S., M.M., and J.B. provided patient samples and contributed to study design. C.Y., V.K., B.D., and M.K.-A. contributed to study design, data analyses, and manuscript preparation. L.A. contributed to study design, performing experiments, and fund raising. M.L. contributed to study design, data analyses, manuscript preparation, fund raising, and overall study coordination.

DECLARATION OF INTERESTS

The authors declare no competing interests.

Received: April 4, 2024
Revised: June 3, 2024
Accepted: June 4, 2024
Published: July 3, 2024

REFERENCES

Abad-Morales, V., Burés-Jelstrup, A., Navarro, R., Ruiz-Nogales, S., MéndezVendrell, P., Corcóstegui, B., and Pomares, E. (2019). Characterization of the cone-rod dystrophy retinal phenotype caused by novel homozygous DRAM2 mutations. Exp. Eye Res. *187*, 107752. https://doi.org/10.1016/j.exer.2019.107752.

Anderson, G.W., Goebel, H.H., and Simonati, A. (2013). Human pathology in NCL. Biochim Biophys Acta *11*, 1807–1826. https://doi.org/10.1016/j.bbadis.2012.11.014.

Bagh, M.B., Peng, S., Chandra, G., Zhang, Z., Singh, S.P., Pattabiraman, N., Liu, A., and Mukherjee, A.B. (2017). Misrouting of v-ATPase subunit V0a1 dysregulates lysosomal acidification in a neurodegenerative lysosomal storage disease model. Nat. Commun. *8*, 14612. https://doi.org/10.1038/ncomms14612.

Bassal, M., Liu, J., Jankowiak, W., Saftig, P., and Bartsch, U. (2021). Rapid and Progressive Loss of Multiple Retinal Cell Types in Cathepsin D-Deficient Mice-An Animal Model of CLN10 Disease. Cells *10*, 696. https://doi.org/10.3390/cells10030696.

Berger, A.C., Salazar, G., Styers, M.L., Newell-Litwa, K.A., Werner, E., Maue, R.A., Corbett, A.H., and Faundez, V. (2007). The subcellular localization of the Niemann-Pick Type C proteins depends on the adaptor complex AP-3. J. Cell Sci. *120*, 3640–3652. https://doi.org/10.1242/jcs.03487.

Chang, T.Y., Reid, P.C., Sugii, S., Ohgami, N., Cruz, J.C., and Chang, C.C.Y. (2005). Niemann-Pick type C disease and intracellular cholesterol trafficking. J. Biol. Chem. *280*, 20917–20920. https://doi.org/10.1074/jbc.R400040200.

Chattopadhyay, S., and Pearce, D.A. (2000). Neural and extraneural expression of the neuronal ceroid lipofuscinoses genes CLN1, CLN2, and CLN3: functional implications for CLN3. Mol. Genet. Metab. *71*, 207–211. https://doi.org/10.1006/mgme.2000.3056.

Chen, H., Tran, J.T.A., Eckerd, A., Huynh, T.P., Elliott, M.H., Brush, R.S., and Mandal, N.A. (2013). Inhibition of *de novo* ceramide biosynthesis by FTY720 protects rat retina from light-induced degeneration. J. Lipid Res. *54*, 1616–1629. https://doi.org/10.1194/jlr.M035048.

Contreras, F.X., Sot, J., Alonso, A., and Goñi, F.M. (2006). Sphingosine increases the permeability of model and cell membranes. Biophys. J. *90*, 4085–4092. https://doi.org/10.1529/biophysj.105.076471.

Dearborn, J.T., Ramachandran, S., Shyng, C., Lu, J.Y., Thornton, J., Hofmann, S.L., and Sands, M.S. (2016). Histochemical localization of palmitoyl protein thioesterase-1 activity. Mol. Genet. Metab. *117*, 210–216. https://doi.org/10.1016/j.ymgme.2015.11.004.

Dockery, A., Whelan, L., Humphries, P., and Farrar, G.J. (2021). Next-generation sequencing applications for inherited retinal diseases. Int. J. Mol. Sci. *22*, 5684. https://doi.org/10.3390/ijms22115684.

Dorgau, B., Georgiou, M., Chaudhary, A., Moya-Molina, M., Collin, J., Queen, R., Hilgen, G., Davey, T., Hewitt, P., Schmitt, M., et al. (2022). Human retinal organoids provide a suitable tool for toxicological investigations: a comprehensive validation using drugs and compounds affecting the retina. Stem Cells Transl. Med. *11*, 159–177. https://doi.org/10.1093/stcltm/szab010.

El-Asrag, M.E., Sergouniotis, P.I., McKibbin, M., Plagnol, V., Sheridan, E., Waseem, N., Abdelhamed, Z., McKeefry, D., Van Schil, K., Poulter, J.A., et al. (2015). Biallelic mutations in the autophagy regulator DRAM2 cause retinal dystrophy with early macular involvement. Am. J. Hum. Genet. *96*, 948–954. https://doi.org/10.1016/j.ajhg.2015.04.006.

Evans, W.R.H., and Hendriksz, C.J. (2017). Niemann-Pick type C disease - the tip of the iceberg? A review of neuropsychiatric presentation, diagnosis and treatment. BJPsych Bull. *41*, 109–114. https://doi.org/10.1192/pb.bp.116.054072.

Fan, J., Liu, J., Liu, J., Chen, C., Koutalos, Y., and Crosson, C.E. (2021). Evidence for ceramide induced cytotoxicity in retinal ganglion cells. Exp. Eye Res. *211*, 108762. https://doi.org/10.1016/j.exer.2021.108762.

Farrar, G.J., Carrigan, M., Dockery, A., Millington-Ward, S., Palfi, A., Chadderton, N., Humphries, M., Kiang, A.S., Kenna, P.F., and Humphries, P. (2017). Toward an elucidation of the molecular genetics of inherited retinal degenerations. Hum. Mol. Genet. *26*, R2–R11. https://doi.org/10.1093/hmg/ddx185.

Foltz, L.P., and Clegg, D.O. (2019). Patient-derived induced pluripotent stem cells for modelling genetic retinal dystrophies. Prog. Retin. Eye Res. *68*, 54–66. https://doi.org/10.1016/j.preteyeres.2018.09.002.

Frohlich, F., Petit, C., Kory, N., Christiano, R., Hannibal-Bach, H.K., Graham, M., Liu, X., Ejsing, C.S., Farese, R.V., and Walther, T.C. (2015). The GARP complex is required for cellular sphingolipid homeostasis. Elife *4*, e08712. https://doi.org/10.7554/eLife.08712.

Frolov, A., Zielinski, S.E., Crowley, J.R., Dudley-Rucker, N., Schaffer, J.E., and Ory, D.S. (2003). NPC1 and NPC2 regulate cellular cholesterol homeostasis through generation of low density lipoprotein cholesterol-derived oxysterols. J. Biol. Chem. *278*, 25517–25525. https://doi.org/10.1074/jbc.M302588200.

German, O.L., Miranda, G.E., Abrahan, C.E., and Rotstein, N.P. (2006). Ceramide is a mediator of apoptosis in retina photoreceptors. Invest. Ophthalmol. Vis. Sci. *47*, 1658–1668. https://doi.org/10.1167/iovs.05-1310.

Hanany, M., Rivolta, C., and Sharon, D. (2020). Worldwide carrier frequency and genetic prevalence of autosomal recessive inherited retinal diseases. Proc. Natl. Acad. Sci. USA *117*, 2710–2716. https://doi.org/10.1073/pnas.1913179117.

Hu, Y.B., Dammer, E.B., Ren, R.J., and Wang, G. (2015). The endosomal-lysosomal system: from acidification and cargo sorting to neurodegeneration. Transl. Neurodegener. *4*, 18. https://doi.org/10.1186/s40035-015-0041-1.

Infante, R.E., Wang, M.L., Radhakrishnan, A., Kwon, H.J., Brown, M.S., and Goldstein, J.L. (2008). NPC2 facilitates bidirectional transfer of cholesterol between NPC1 and lipid bilayers, a step in cholesterol egress from lysosomes. Proc. Natl. Acad. Sci. USA *105*, 15287–15292. https://doi.org/10.1073/pnas.080732810.

Jalanko, A., and Braulke, T. (2009). Neuronal ceroid lipofuscinoses. Biochim. Biophys. Acta *1793*, 697–709. https://doi.org/10.1016/j.bbamcr.2008.11.004.

Jones, M.K., Orozco, L.D., Qin, H., Truong, T., Caplazi, P., Elstrott, J., Modrusan, Z., Chaney, S.Y., and Jeanne, M. (2023). Integration of human stem cell-derived *in vitro* systems and mouse preclinical models identifies complex pathophysiologic mechanisms in retinal dystrophy. Front. Cell Dev. Biol. *11*, 1252547. https://doi.org/10.3389/fcell.2023.1252547.

Kim, J.K., Lee, H.M., Park, K.S., Shin, D.M., Kim, T.S., Kim, Y.S., Suh, H.W., Kim, S.Y., Kim, I.S., Kim, J.M., et al. (2017). MIR144* inhibits antimicrobial responses against Mycobacterium tuberculosis in human monocytes and macrophages by targeting the autophagy protein DRAM2. Autophagy *13*, 423–441. https://doi.org/10.1080/15548627.2016.1241922.

Krašovec, T., Volk, M., Šuštar Habjan, M., Hawlina, M., Vidović Valentinčič, N., and Fakin, A. (2022). The clinical spectrum and disease course of DRAM2 retinopathy. Int. J. Mol. Sci. *23*, 7398. https://doi.org/10.3390/ijms23137398.

Kuniyoshi, K., Hayashi, T., Kameya, S., Katagiri, S., Mizobuchi, K., Tachibana, T., Kubota, D., Sakuramoto, H., Tsunoda, K., Fujinami, K., et al. (2020). Clinical course and electron microscopic findings in lymphocytes of patients with DRAM2-associated retinopathy. Int. J. Mol. Sci. *21*, 1331. https://doi.org/10.3390/ijms21041331.

Kwon, H.J., Abi-Mosleh, L., Wang, M.L., Deisenhofer, J., Goldstein, J.L., Brown, M.S., and Infante, R.E. (2009). Structure of N-terminal domain of NPC1 reveals distinct subdomains for binding and transfer of cholesterol. Cell *137*, 1213–1224. https://doi.org/10.1016/j.cell.2009.03.049.

Leloir, L.F. (1977). The role of dolichol in protein glycosylation. Adv. Exp. Med. Biol. *83*, 9–19. https://doi.org/10.1007/978-1-4684-3276-3_3.

Levitsky, Y., Hammer, S.S., Fisher, K.P., Huang, C., Gentles, T.L., Pegouske, D.J., Xi, C., Lydic, T.A., Busik, J.V., and Proshlyakov, D.A. (2020). Mitochondrial Ceramide Effects on the Retinal Pigment Epithelium in Diabetes. Int. J. Mol. Sci. *21*, 3830. https://doi.org/10.3390/ijms21113830.

Lloyd-Evans, E., Morgan, A.J., He, X., Smith, D.A., Elliot-Smith, E., Sillence, D.J., Churchill, G.C., Schuchman, E.H., Galione, A., and Platt, F.M. (2008). Niemann-Pick disease type C1 is a sphingosine storage disease that causes deregulation of lysosomal calcium. Nat. Med. *14*, 1247–1255. https://doi.org/10.1038/nm.1876.

Maeda, T., and Takahashi, M. (2023). iPSC-RPE in Retinal Degeneration: Recent Advancements and Future Perspectives. Cold Spring Harbor Perspect. Med. *13*, a041308. https://doi.org/10.1101/cshperspect.a041308.

Nabi, I.R., and Dennis, J.W. (1998). The extent of polylactosamine glycosylation of MDCK LAMP-2 is determined by its Golgi residence time. Glycobiology *8*, 947–953. https://doi.org/10.1093/glycob/8.9.947.

Neveling, K., Collin, R.W.J., Gilissen, C., van Huet, R.A.C., Visser, L., Kwint, M.P., Gijsen, S.J., Zonneveld, M.N., Wieskamp, N., de Ligt, J., et al. (2012). Next-generation genetic testing for retinitis pigmentosa. Hum. Mutat. *33*, 963–972. https://doi.org/10.1002/humu.22045.

O'Prey, J., Skommer, J., Wilkinson, S., and Ryan, K.M. (2009). Analysis of DRAM related proteins reveals evolutionarily conserved and divergent roles in the control of autophagy. Cell Cycle *8*, 2260–2265. https://doi.org/10.4161/cc.8.14.9050.

Park, S.M., Kim, K., Lee, E.J., Kim, B.K., Lee, T.J., Seo, T., Jang, I.S., Lee, S.H., Kim, S., Lee, J.H., and Park, J. (2009). Reduced expression of DRAM2/TMEM77 in tumor cells interferes with cell death. Biochem. Biophys. Res. Commun. *390*, 1340–1344. https://doi.org/10.1016/j.bbrc.2009.10.149.

Park, W.D., O'Brien, J.F., Lundquist, P.A., Kraft, D.L., Vockley, C.W., Karnes, P.S., Patterson, M.C., and Snow, K. (2003). Identification of 58 novel mutations in Niemann-Pick disease type C: correlation with biochemical phenotype and importance of PTC1-like domains in NPC1. Hum. Mutat. *22*, 313–325. https://doi.org/10.1002/humu.10255.

Pentchev, P.G. (2004). Niemann-Pick C research from mouse to gene. Biochim. Biophys. Acta *1685*, 3–7. https://doi.org/10.1016/j.bbalip.2004.08.005.

Puri, V., Watanabe, R., Dominguez, M., Sun, X., Wheatley, C.L., Marks, D.L., and Pagano, R.E. (1999). Cholesterol modulates membrane traffic along the endocytic pathway in sphingolipid-storage diseases. Nat. Cell Biol. *1*, 386–388. https://doi.org/10.1038/14084.

Radhakrishnan, A., Goldstein, J.L., McDonald, J.G., and Brown, M.S. (2008). Switch-like control of SREBP-2 transport triggered by small changes in ER cholesterol: a delicate balance. Cell Metab. *8*, 512–521. https://doi.org/10.1016/j.cmet.2008.10.008.

Regent, F., Morizur, L., Lesueur, L., Habeler, W., Plancheron, A., M'Barek, K., and Monville, C. (2019). Automation of human pluripotent stem cell differentiation toward retinal pigment epithelial cells for large-scale productions. Sci. Rep. *9*, 10646. https://doi.org/10.1038/s41598-019-47123-6.

Retinal Information Network. https://web.sph.uth.edu/RetNet/home.htm.

Ribeiro, I., Marcão, A., Amaral, O., Sá Miranda, M.C., Vanier, M.T., and Millat, G. (2001). Niemann-Pick type C disease: NPC1 mutations associated with severe and mild cellular cholesterol trafficking alterations. Hum. Genet. *109*, 24–32. https://doi.org/10.1007/s004390100531.

Rip, J.W., Chaudhary, N., and Carroll, K.K. (1983). Distribution and metabolism of dolichol and dolichyl phosphate in rat liver. Can. J. Biochem. Cell Biol. *61*, 1025–1031. https://doi.org/10.1139/o83-131.

Santavuori, P. (1988). Neuronal ceroid-lipofuscinoses in childhood. Brain Dev. *10*, 80–83. https://doi.org/10.1016/s0387-7604(88)80075-5.

Santavuori, P., Haltia, M., Rapola, J., and Raitta, C. (1973). Infantile type of so-called neuronal ceroidlipofuscinosis. 1. A clinical study of 15 patients. J. Neurol. Sci. *18*, 257–267. https://doi.org/10.1016/0022-510x(73)90075-0.

Schulz, A., Kohlschütter, A., Mink, J., Simonati, A., and Williams, R. (2013). NCL diseases - clinical perspectives. Biochim. Biophys. Acta *1832*, 1801–1806. https://doi.org/10.1016/j.bbadis.2013.04.008.

Sergouniotis, P.I., McKibbin, M., Robson, A.G., Bolz, H.J., De Baere, E., Müller, P.L., Heller, R., El-Asrag, M.E., Van Schil, K., Plagnol, V., et al. (2015). Disease Expression in Autosomal Recessive Retinal Dystrophy Associated With Mutations in the DRAM2 Gene. IOVS (Investig. Ophthalmol. Vis. Sci.) *56*, 8083–8090. https://doi.org/10.1167/iovs.15-17604.

Vanier, M.T. (1999). Lipid changes in Niemann-Pick disease type C brain: personal experience and review of the literature. Neurochem. Res. *24*, 481–489. https://doi.org/10.1023/a:1022575511354.

Vanier, M.T., and Millat, G. (2003). Niemann-Pick disease type C. Clin. Genet. *64*, 269–281. https://doi.org/10.1034/j.1399-0004.2003.00147.x.

Volland, S., Esteve-Rudd, J., Hoo, J., Yee, C., and Williams, D.S. (2015). A comparison of some organizational characteristics of the mouse central retina and the human macula. PLoS One *10*, e0125631. https://doi.org/10.1371/journal.pone.0125631.

Weleber, R.G. (1998). The dystrophic retina in multisystem disorders: the electroretinogram in neuronal ceroid lipofuscinoses. Eye *12*, 580–590. https://doi.org/10.1038/eye.1998.148.

Weleber, R.G., Gupta, N., Trzupek, K.M., Wepner, M.S., Kurz, D.E., and Milam, A.H. (2004). Electroretinographic and clinicopathologic correlations of retinal dysfunction in infantile neuronal ceroid lipofuscinosis (infantile Batten disease). Mol. Genet. Metab. *83*, 128–137. https://doi.org/10.1016/j.ymgme.2004.06.019.

Williams, R.E., and Mole, S.E. (2012). New nomenclature and classification scheme for the neuronal ceroid lipofuscinoses. Neurology *79*, 183–191. https://doi.org/10.1212/WNL.0b013e31825f0547.

Winkler, P.A., Occelli, L.M., and Petersen-Jones, S.M. (2020). Large Animal Models of Inherited Retinal Degenerations: A Review. Cells *9*, 882. https://doi.org/10.3390/cells9040882.

Wu, W.I., Lin, Y.P., Wang, E., Merrill, A.H., Jr., and Carman, G.M. (1993). Regulation of phosphatidate phosphatase activity from the yeast Saccharomyces cerevisiae by sphingoid bases. J. Biol. Chem. *268*, 13830–13837. https://doi.org/10.1016/S0021-9258(19)85178-5.

Yoon, J.-H., Her, S., Kim, M., Jang, I.-S., and Park, J. (2012). The expression of damage-regulated autophagy modulator 2 (DRAM2) contributes to autophagy induction. Mol. Biol. Rep. *39*, 1087–1093. https://doi.org/10.1007/s11033-011-0835-x.

Zeng, C.W., Chen, Z.H., Zhang, X.J., Han, B.W., Lin, K.Y., Li, X.J., Wei, P.P., Zhang, H., Li, Y., and Chen, Y.Q. (2014). MIR125B1 represses the degradation of the PML-RARA oncoprotein by an autophagy-lysosomal pathway in acute promyelocytic leukemia. Autophagy *10*, 1726–1737. https://doi.org/10.4161/auto.29592.

Stem Cell Reports, Volume *19*

Supplemental Information

Retinal cells derived from patients with DRAM2-dependent CORD21 dystrophy exhibit key lysosomal enzyme deficiency and lysosomal content accumulation

Rozaliya Tsikandelova, Eldo Galo, Edvinas Cerniauskas, Dean Hallam, Maria Georgiou, Rodrigo Cerna-Chavez, Robert Atkinson, Pavel Palmowski, Florence Burté, Tracey Davies, David H. Steel, Martin McKibbin, Jacquelyn Bond, Jennifer Haggarty, Phil Whitfield, Viktor Korolchuk, Lyle Armstrong, Chunbo Yang, Birthe Dorgau, Marzena Kurzawa-Akanbi, and Majlinda Lako

Retinal cells derived from patients with DRAM2-dependent CORD21 dystrophy exhibit key lysosomal enzyme deficiency and lysosomal content accumulation

Rozaliya Tsikandelova [1], Eldo Galo [1], Edvinas Cerniauskas [1], Dean Hallam [1], Maria Georgiou [1], Rodrigo Cerna-Chavez [1], Robert Atkinson [1], Pavel Palmowski [1], Florence Burté [1], Tracey Davies [2], David H Steel [1], Martin McKibbin [3], Jacquelyn Bond [3], Jennifer Haggarty [4], Phil Whitfield [5], Viktor Korolchuk [1], Lyle Armstrong [1], Chunbo Yang [1], Birthe Dorgau [1], Marzena Kurzawa-Akanbi [1] and Majlinda Lako [1#]

1. Biosciences Institute, Newcastle University, UK
2. Electron Microscopy Research Services, Newcastle University, UK
3. Leeds Teaching Hospitals NHS Trust, Leeds UK and Leeds Institute for Medical Research, St. James's University Hospital, University of Leeds, UK
4. Shared Research Facilities, College of Medical, Veterinary and Life Sciences, University of Glasgow, Glasgow G12 8QQ.
5. Glasgow Polyomics and Institute of Infection, Immunity and Inflammation, College of Medical, Veterinary and Life Sciences, University of Glasgow, Glasgow, UK.

[#] Lead contact and corresponding information:

Majlinda Lako

Biosciences Institute

Newcastle University

United Kingdom

Email: majlinda.lako@ncl.ac.uk

Supplementary Information contains:

Detailed Experimental Procedures

Supplementary Figures 1-8

Supplementary Table Legends

Detailed Experimental Procedures

Cell culture

Dermal skin fibroblast culture

Dermal skin fibroblasts were isolated from individuals diagnosed with CORD21 dystrophy following acquisition of informed written consent in accordance with the Yorkshire and the Humber Research Ethics Committee (REC ref. no. 15/YH/0365). Dermal fibroblasts were maintained in advanced Dulbecco's modified Eagle Medium (Thermo Fisher Scientific) supplemented with 10% FBS (Thermo Fisher Scientific), 1% Glutamax (Thermo Fisher Scientific) and 1% pen/strep (Thermo Fisher Scientific).

iPSC reprogramming

Dermal fibroblasts were transduced at a density of 30,000 cells/cm^2 using the CytoTune™-iPS 2.0 Reprogramming Kit (Life Technologies, A16517) following the manufacturer's instructions. The established patient iPSCs were assessed for pluripotency and clearance of Sendai virus-associated transgenes using primers shown in **Table S1**. The *DRAM2* gene mutations were corroborated by PCR using primer pairs shown in **Table S1**, followed by Sanger sequencing. In addition, iPSCs derived from dermal fibroblasts from two age-matched individuals (WT1 and WT3, collectively named WT) with no history of retinal disease were used as healthy controls as outlined in Buskin et al. 2018. Further information about the cell lines is available in **Table S1.**

iPSC culture

iPSCs were maintained at standard cell culture incubator conditions at 95% humidity, 5% C02 and 37°C. CORD21- and WT-iPSCs were grown and expanded on Matrigel™ Growth Factor Reduced Basement Membrane Matrix (Corning) using mTeSR™ Plus cell culture media (STEMCELL Technologies) supplemented with 1% penicillin-streptomycin (Thermo Fisher Scientific). Cells were passaged in a 1: 6 ratio every 4-5 days using 0.02% Versene- EDTA (Lonza) for 3–5 minutes at 37 °C. All iPSCs as well as differentiated retinal cultures were routinely tested for the presence of mycoplasma using the MycoAlert® Mycoplasma Detection Kit (Lonza, LT07-118) every 2-3 months.

Generation of the CORD21-P1c and -P2c heterozygous isogenic control iPSCs

The generation of CORD21-P1c and CORD21-P2c isogenic control iPSCs was enabled by the single nucleotide CRISPR/Cas9 correction of the c.140delG and the c. 131G>A mutations present in the CORD21-P1 and -P2 iPSCs, respectively. Target gRNA and ssODN sequences were designed using the Benchling CRISPR online tool (https://www.benchling.com/crispr) (**Table S1**). gRNA was generated using the GeneArt Precision gRNA Synthesis Kit (Thermo Fisher Scientific, A29377) following the

manufacturer's instructions. ssODN was synthesized by Thermo Fisher Scientific. Nucleofection was performed using P3 Primary Cell 4DNucleofectorTM X Kit according to the manual's instructions (Lonza, V4XP-3024). To boost the efficiency of HDR recombination, ~ $1x10^6$ CORD21-iPSCs were treated with 10 µM of NHEJ inhibitor SRC-7 (Sigma Aldrich, SML1546) for 8 hours prior to nucleofection. 200pM gRNA and 100pM TrueCut™ Cas9 Protein v2 enzyme (Thermo Fisher Scientific, A36497) were combined at room temperature (RT) for 10 minutes to allow for the formation of ribonucleoprotein complexes (RNPs). iPSCs were dissociated using StemPro Accutase for 5 minutes at 37° C (Gibco, A11105- 01) and centrifuged in the presence of 10µM Rock inhibitor (Y-27632, Fischer scientific, CD0141). gRNA/Cas9 RNPs and 200pM ssODN were added to iPSCs (Cas9 to gRNA/ssODN 1:2 ratio) and nucleofection was performed on a 4D-Nucleofector™ (Lonza). Matrigel™-coated Petri dishes were seeded with a drop of nucleofected solution and single cells were cultured in the presence of 10µM SRC-7 and 10µM Rock inhibitor. Medium was replaced within 24 hours and replenished every two days. Upon expansion of single-cell colonies, individual colonies were cultured on Matrigel™-coated 24-well plates followed by restriction digest analysis (CORD21-P1c, *HpyCH4V*, NEB, R0620L; CORD21-P2c, *Alu I*, NEB, R0137S). Clones showing correct band sizes were further sequenced to corroborate the successful correction of *DRAM2* mutant alleles (primers shown in **Table S1**).

Off-target sequencing

The online platform http://www.rgenome.net/cas-offinder/ was used to identify genomic regions bearing high sequence homology to the sites of CRISPR/Cas9 correction. Query sequences were allowed a maximum of up to three mismatches. Top 10 sequences exhibiting the highest sequence homology were selected and primers enclosing the respective genomic regions were designed for off-target sequencing as shown in **Table S1**.

Retinal Organoid Differentiation

iPSCs cultured in 6 well-plates were washed with PBS, dissociated using Accutase at about 80%-90% confluence (Gibco, Thermo Fisher Scientific) and seeded at a density of 7000 cells/well on U-bottom Lipidure pre-coated 96-well plates (AMSbio) in the presence of 10 µM ROCK inhibitor (Y27632, Tocris) (D-2). Upon formation of embryoid bodies (D0), differentiation medium was added to each well (41% IMDM, 41% HAM's F12, 15% KOSR, 1% GlutaMAX, 1% Chemically Defined Lipid Concentrate, 1% Pen/Strep (Thermo Fisher Scientific), and 225 µM 1-thioglycerol (M6145; Sigma)) and cultures were fed by carrying out half-media changes every two days. Differentiation medium was supplemented once with 2.25nM BMP4 (R&D, 314-BP-050/CF) at day 6, and medium was changed every three days until day 18. Subsequently, ROs were cultured in maintenance media (DMEM/F12, 10% FBS, 1%

GlutaMAX, 1% N2, 1% Pen/ Strep, 0.1 mM Taurine, 0.25 µg/ mL Fungizone), whereby 0.5 µM retinoic acid (RA) was added from day 18 to day 120 (Sigma Aldrich, R2625). Cultures were maintained by partial medium changes three times a week. A schematic of the RO differentiation protocol is shown in **Figure 1A**.

Immunofluorescence analysis of ROs

The organoid sections were left to dry at RT for an hour and washed 3x5 minutes with PBS (Thermo Fisher Scientific) to remove any remaining OCT. To minimize non-specific binding, sections were blocked for 1 hour at RT with a solution of PBS containing 10% goat serum and 0.3% Triton-X100. Sections were then double stained overnight at 4°C in a humidifier chamber using primary antibodies diluted in AD (Antibody diluent) buffer (0.001% BSA-PBS and 0.3% Triton-X, Sigma Aldrich, USA) (**Table S1**). Following incubation overnight, RO sections were washed 3x 10 minutes with AD buffer. Secondary antibodies diluted in PBS (1:1000) (**Table S1**) were applied to the sections and incubated for 2 hours at RT. Finally, the sections were washed three times with PBS for 15 minutes. Nuclei were counterstained with a 1:1000 dilution of Hoechst (cat. No. 33342, Thermo Fisher Scientific) in Vectashield (cat. No. H-1000-10, Vector Laboratories, CA). The slides were then covered with 24x60 mm coverslips, sealed on each side with nail polish, and stored at 4°C in the short term. For quality control, sections stained only with a secondary antibody were analysed for each staining to ensure the specificity of the fluorescent antibody signal. High-resolution fluorescence imaging was facilitated by optical sectioning on an Axio Imager microscope (Zeiss, Apotome, 10-15 Z-stacks). Images were acquired as maximum intensity projections on Zen (Zeiss). Quantification of immunostained RO sections were carried out on MATLAB (Mathworks) based on a protocol by Dorgau et al. (2019).

RPE differentiation

The differentiation procedure was commenced at 100% iPSC confluency (**Figure 1E**). During the initial 7 days of differentiation (D0-D7), the cells were cultured in DMEM/F-12, GlutaMAX™ medium (Thermo Fisher Scientific, 10565018) supplemented with 50µM β-mercaptoethanol (Sigma-Aldrich, M3148), 1xMEM NEAA (Thermo Fisher Scientific, 11140068), 20% KnockOut Serum Replacement (Thermo Fisher Scientific, 10828028) and 10 mM Nicotinamide (Sigma Aldrich, N0636). From day 7 to day 14 (D7-D14), nicotinamide was replaced by 100 ng/mL Activin A (Preprotech, 12014E-250UG). Between D14-D42, Activin A was substituted by 3 µM CHIR99021 (Sigma-Aldrich, SML1046). CHIR99021 supplementation was withdrawn at D42-84. Nascent RPE cells were allowed further time to mature in DMEM/F-12, GlutaMAX™ (Thermo Fisher Scientific, 10565018) medium containing 50µM β-mercaptoethanol (Sigma-Aldrich, M3148), 1xMEM NEAA (Thermo Fisher Scientific, 11140068) and 4%

KnockOut Serum Replacement (Thermo Fisher Scientific, 10828028). Fully mature and pigmented RPE cells seeded on hanging inserts showing TEER values > 250 Ω cm^2 were used for experiments described in this study.

Immunofluorescence analysis of RPE cells

Immunofluorescence analyses of RPE cells were performed as qualitative experiments using Zen software (Zeiss). In short, transwells were rinsed in PBS and incubated with 4% PFA for 30 minutes. The tissue was subsequently washed 3x5 minutes with PBS and flat RPE sheets were cut into multiple pieces. Additional fixation by methanol was performed at 4°C for 20 minutes when staining for tight junctions (ZO-1) and Collagen IV. To remove melanin pigment from the RPE a bleaching procedure was carried out using a Melanin Bleach kit (cat. No 24883, Polysciences), following the manufacturer's instructions. To minimize non-specific binding, RPE were then blocked and permeabilized simultaneously for 1 hour in PBS containing 10% Donkey Serum (cat. No. 7332100-LAM, Stratech) and 0.3% Triton-X-100. Primary antibodies were diluted in PBS containing 0.1% Triton-X-100 and 1% Donkey Serum-PBS and applied at 4°C overnight. The tissue was subsequently rinsed with PBS (3x5 minutes). Secondary antibodies diluted in PBS were incubated for 1 hour at RT. RPE sections were washed 3x5 minutes in PBS and counterstained for nuclei using Hoechst (1:1000 in PBS) for approximately 20 minutes. After an additional PBS wash, RPE sections were mounted on slides using Vectashield®. A secondary antibody only control was included in each set of experiments. For all antibody details please refer to **Table S1**.

Transepithelial electrical resistance (TEER)

Transepithelial electrical resistance was routinely conducted as a functional measurement of RPE barrier function using a volt-ohm meter (Millipore, MERS00002). RPE cells were equilibrated to RT, and a sterilized electrode was placed on either side of the transwell membranes. To determine unit area resistance (Ω · cm2), values pertaining to inserts containing media but devoid of cells (blanks) were subtracted from sample readings. Obtained values were multiplied by the surface area of the insert (0.33 cm^2 for a 24-well plate insert). RPE monolayer was considered mature for further analyses at TEER > 250 Ω cm^2. Measurements were taken in triplicates every two to three weeks.

Phagocytosis assay

<u>Labelling of photoreceptor outer segments (POS) with fluorescein isothiocyanate</u>

Bovine rod POSs (InVision BioResources, 98740) were labelled with 0.4mg/mL fluorescein isothiocyanate (FITC) (Sigma Aldrich, F7367) in basal RPE medium. The light sensitive POS solution was

placed on a shaking incubator shielded from light for 1 hour at RT. FITC-labelled POSs were washed 3x5 minutes with PBS, reconstituted at 10^6 POSs/mL in 73mM sucrose-PBS (Sigma Aldrich, S0389) and stored at -80°C.

<u>Flow cytometry analysis of phagocytic activity</u>

FITC-labelled POS were diluted in 10% FBS containing RPE medium and incubated with RPE on transwell inserts for 4 hours at 37°C. RPE cells were rinsed with PBS and dissociated into a single-cell suspension using TrypLE™ Select Enzyme (10x). The live cell dye DRAQ5™ (Abcam, ab108410) was resuspended in flow buffer (2% FBS in PBS) at a ratio of 1:40 and incubated with the dissociated cells for 10 minutes at 37°C. To quench any residual FITC fluorescence, cells were treated with 0.2% Trypan Blue for 10 minutes (Sigma Aldrich, 93595). Samples were centrifuged and further rinsed with 2% FBS-PBS. Flow cytometry analysis was conducted on a BD™ LSR II flow cytometer (BD Biosciences) with 10,000 events acquired for each sample. A sample kept at 4°C served as a negative control.

POS treatment of RPE cells

RPE were treated with 20 POSs/cell every day for 14 days and subjected to transmission electron microscopy analysis. An untreated sample was included as an internal control for each RPE cell line.

ELISA Detection of VEGF and PEDF secretion

A 96-well microplate was coated overnight with diluted Capture Antibody. Unbound antibodies were removed by washing and plate was blocked to prevent non-specific binding using Reagent Diluent at room temperature (RT). Following a round of washing, 100 µL of basal RPE media samples and standards were applied to the wells and incubated for 2 hours at RT. Following an additional washing step, the wells were treated with 100 µL Streptavidin-HRP for 20 minutes at RT in the dark. The plate was rinsed further to remove unbound HRP. To facilitate detection, substrate solution was added to the samples for 20 minutes. The reaction was terminated by the addition of a Stop Solution and optical density was measured at 450 nm using a Varioskan LUX Multimode Microplate ELISA reader (Thermo Fisher Scientific). An analogous procedure was carried out to determine PEDF levels in the apical compartment of RPE cells (insert media) using a Human Serpin F1/PEDF DuoSet ELISA kit by adhering to the manual's instructions (Biotechne, DY1177-05).

PCR

<u>RNA extraction</u>

RNA extraction from RO pellets was performed in a laminar flow hood. The hood was UV sterilized and decontaminated from RNA nucleases using RNaseZap™ (Thermo Fisher Scientific, AM9780). RNA was extracted using the ReliaPrep™ RNA Cell Miniprep System following the manufacturer's instructions (Promega, Z6012).

cDNA synthesis

First strand cDNA synthesis was initiated by converting up to 5 µg of RNA in a 5 µL reaction containing primer Oligo(dT)15 and Nuclease-Free Water (NFW). The same amount of RNA was converted across all biological replicates based on the sample showing the lowest RNA concentration. Samples were placed onto a pre-heated block at 70°C for 5 minutes and subsequently pre-chilled at 4°C for another 5 minutes. Each of the samples was centrifuged to remove lid condensation and placed briefly on ice. A reverse transcriptase mix was prepared using GoScript™ 5X Reaction buffer, $MgCl_2$, dNTPs, RNasin Ribonuclease Inhibitor and GoScript Reverse Transcriptase in accordance with the guidance manual. 5 µL RNA and 15 µL of the mix were then carefully mixed and RNA was converted to cDNA at the thermocycler conditions outlined in the manual. cDNA was stored at -20°C until further use.

RT-qPCR

To determine *DRAM2a* isoform expression by RT-qPCR, RNA samples were cleared from any potential DNA contamination using a TURBO DNA-free™ kit (Thermo Fisher Scientific, AM1907). Equal amounts of cDNA template were used to set up a 384-well qPCR reaction using a GoTaq qPCR Master mix kit (Promega, A6002) on Quantstudio™ 7 Flex Real-Time PCR system (Applied Biosystems). Data were normalized to the expression of *GAPDH* and gene expression was calculated using the $2^{(-\Delta\Delta CT)}$ method. Samples were run in triplicates and data were presented as means + SEM. *DRAM2* oligonucleotide sequences are shown in **Table S1**.

DRAM2 siRNA knockdown

To improve siRNA uptake efficiency, day 230 WT ROs were dissected and cultured on Matrigel™ Growth Factor Reduced Basement Membrane Matrix (Corning), and poly-L-ornithine (10 µg/mL) (Sigma Aldrich, A-004-C) coated 24-well plates for a single round of passaging. siRNA transfection was performed at 50% confluence using a Lipofectamine™ RNAiMAX Transfection reagent (Thermo Fisher Scientific, 13778030). 20µM *DRAM2* Silencer Select siRNA (4392420, Assay ID: s43281) and scrambled control (Thermo Fisher Scientific, 4390849) were incubated with the dissected ROs in an antibiotic and serum-free maintenance media for 72 hours and collected for qPCR and WB analysis.

Western blot

BCA protein assay

Cell pellets stored at -80°C were lysed for 5 minutes at RT in PhosphoSafe™ extraction buffer (Merck Millipore, 71296) supplemented with protease inhibitor cocktail Complete Mini EDTA-free (Roche, 04693159001). Rigorous pipetting was alternated by vortexing and rest on ice for 20 minutes until samples were completely lysed. Samples were spun at 4°C for 10 minutes at 1000xg, and the concentration of the sample supernatants was determined using a Pierce™ BCA Protein Assay Kit (Thermo Fisher Scientific, 23225). Absorbance was measured at 562 nm on a Varioskan LUX Multimode Microplate Reader (Thermo Fisher Scientific).

Media sample collection and concentration

Media samples were collected for ROs at D220 and from RPE which had been starved for 7 days. Prior to use, Amicon 10 kDa MWCO ultrafiltration units (Merck, UFC801096) were washed with 4 mL sterile distilled water and centrifuged for 10 minutes at 3000xg. 5 mL of media was loaded onto membranes and concentrated to a final volume of 500 µL, whereby protease inhibitors cocktail was added to each sample (Complete Mini EDTA-free, Roche, 04693159001).

SDS-PAGE

4x NuPAGE™ LDS Sample Buffer (Thermo Fisher Scientific, NP0007) and 10x NUPAGE Sample reducing agent (Thermo Fisher Scientific, NP0004) were added to 10 µg of total protein lysate. Samples were subsequently heated for 10 minutes at 70°C and loaded onto precast 4-12% gradient polyacrylamide Bis-Tris gels (Thermo Fisher Scientific, NP0321PK2, WG1403BOX). NuPAGE™ MES SDS Running Buffer (20x) (Thermo Fisher Scientific, NP0002) and MOPS SDS Running Buffer (20x) (Thermo Fisher Scientific, NP0001) were used for the separation of small (<70kDa) to large (>70kDa) molecular targets, respectively. PageRuler™ Plus Prestained Protein Ladder 10 to 250kDa (Thermo Fisher Scientific, 26619) or SeeBlue™ Plus2 Prestained Protein standard (Thermo Fisher Scientific, LC5925) served as molecular weight reference. Please refer to **Table S1** for a comprehensive description of the Western blot conditions for individual protein targets.

Gel transfer

Dry transfer was performed on an iBlot 2 Dry Blotting system (Thermo Fisher Scientific) using Blot™ 2 Transfer PVDF Stacks (Thermo Fisher Scientific, IB24002 and IB24001) (20V for 1 minute, 23V for 4 minutes and 25V for 2 minutes). Transfer of autophagy markers, including DRAM2, was carried out at 10V for a total of 7 minutes.

Reversible total protein staining

Equal loading of protein samples for WB was corroborated using the Pierce™ Reversible Protein Stain Kit following the manual's instructions (Thermo Fisher Scientific, 24585). PVDF membranes were washed with ultrapure ddH20 and incubated with Memcode™ Sensitizer for 2 minutes under shaking conditions. Subsequently, membranes were stained with a total protein stain for 1 minute and rinsed three times with Memcode™ Destain. Rigorous washing was carried out on a shaker with a 1:1 Methanol/Destain solution, followed by an additional brief 8 washes with ddH20 to reduce the background staining produced by the reversible total protein stain. Membranes were imaged colorimetrically on an Amersham Imager 600 (GE Healthcare). Finally, the staining was erased following a 10 minute incubation with a 1:1 Eraser/Methanol solution and membranes were briefly washed with ultrapure ddH20.

Membrane blocking and antibody incubation

Membranes were blocked for non-specific binding with 5% milk TBST (Tris-buffered saline with 0.1% Tween®20 detergent) at RT for 1 hour. Incubation with primary antibodies in 5% milk TBST was carried out on a rotating platform overnight at 4°C (**Table S1**). Membranes were then washed 3x 5 minutes with TBST at RT. Secondary antibodies were incubated at 1:1000 for 1 hour at RT (**Table S1**) and washed 3x 5 minutes with TBST. To enable visualization, chemiluminescence substrate was prepared using the SuperSignal™ West Pico PLUS Chemiluminescent Substrate kit (Thermo Fisher Scientific, 34579). Image acquisition was carried out on an Amersham Imager 600 (GE Healthcare). Band quantitation analysis was performed on Image J (NIH).

Detection of low-abundance protein

For detection of low-abundance proteins, PVDF membranes post protein transfer were washed with ultrapure ddH20 briefly and pre-treated with an antigen pretreatment solution for 10 minutes at RT (SuperSignal™ Western Blot Enhancer, 46640). After blocking for non-specific binding with 5% milk TBST, membranes were rinsed in TBST for an additional 5 minutes and incubated with primary antibody in antibody diluent overnight at 4°C (SuperSignal™ Western Blot Enhancer). Following 3 washes with TBST (3x 20 minutes), secondary antibodies were incubated for 1 hour at RT at 1:20 000-1:100 000. For optimized signal detection membranes were rigorously washed in TBST for an additional 1 hour prior to being visualized using the SuperSignal™ West Femto Maximum Sensitivity Substrate kit (Thermo Fisher Scientific, 34094). Please refer to **Table S1** for more details.

Lysosomal activity assays

CTSD kinetic activity assay

To preserve enzymatic activity the RPE and RO samples were lysed in native buffer (0.2M Triethylammonium bicarbonate buffer, Sigma Aldrich, T7408) containing protease inhibitors cocktail Complete Mini EDTA-free (Roche, 04693159001). Protein concentration was measured using the Pierce^{TM} BCA Protein Assay Kit (Thermo Fisher Scientific, 23225). 50 µM MCA (7-Methoxycoumarin-4-acetic acid) diluted in assay buffer was used to generate a standard curve (0-25 µM) (Thermo Fisher Scientific, 265301). The kinetic assay was performed on black clear bottom 96-well plates (Thermo Fisher Scientific) in a total volume of 100 µL assay buffer. 5 µg protein lysate was diluted in 50mM sodium acetate assay buffer pH 4.0 (Thermo Fisher Scientific, AM9740). Protein lysate pre-incubated with 0.2mg/mL pepstatin inhibitor at 37°C for 15 minutes (Enzo Life Sciences) served as a negative control. Immediately prior to the initiation of the kinetic assay, Cathepsin D & E substrate (Enzo Life Sciences) was applied at a final concentration of 80µM. Fluorescence signal kinetics were recorded every 5 minutes over the course of 20 minutes upon addition of the substrate at 37°C with intermittent shaking. Measurements were taken at 320nm/400nm excitation/emission spectra using a Varioskan LUX Multimode Microplate Reader (Thermo Fisher Scientific) (Kurzawa-Akanbi et al., 2021).

Glucosylceramidase and Alpha-mannosidase activity assays

The glucosylceramidase activity assay (Abcam, 273339) was conducted in a total volume of 160 µL. 20µM 4-Methylumbelliferone Standard (4-MU) prepared in Assay Buffer was used to generate a standard curve (0-20µM). The samples were lysed using the provided Assay Buffer solution and concentration was measured using a Pierce^{TM} BCA Protein Assay Kit (Thermo Fisher Scientific, 23225). 20x diluted substrate was added to 30 µg of protein lysate diluted in Assay Buffer. Samples were incubated on a white flat bottomed 96-well plate (Appleton Woods Ltd, SS246) at 37°C degrees for 30 minutes under minimal light exposure. Following incubation, 100 µL Glucosylceramidase Stop buffer was added to terminate the reaction in all samples. Fluorescence intensity was measured at 360nm/445nm excitation/emission spectra in end-point mode at 37°C. Alpha-mannosidase activity in the samples was determined analogously using a fluorometric end-point mode Alpha-mannosidase activity assay kit (Abcam, 282917).

Proteomics

Protein digestion

10 µg of total RO protein (5 µL) (n=3-4 different differentiation experiments/sample) were digested using S-Trap micro spin columns (Protifi, NY, USA). Each sample was denatured in 25 µL 5% SDS 50mM Triethylammonium bicarbonate (TEAB) (pH 8.5). The proteins were then reduced with 20mM DTT at 65°C for 30 minutes and alkylated with 40mM iodoacetamide at RT in the dark for 30 minutes. Acidification was achieved by adding 27.5% phosphoric acid to a final concentration of 2.5% (v/v). Protein sample was subsequently bound onto the micro spin columns in 6 volumes of loading buffer (90% methanol 100mM TEAB pH 8.0) and spun at 4000xg for 30s. Columns were rinsed 3x with loading buffer and flow-through was discarded. Protein digestion with trypsin (Worthington) was performed overnight at 37°C in 50mM TEAB pH 8.5, at a ratio of 10:1 protein to trypsin. Peptide elution was carried out over three consecutive washes: first 50 µL 50mM TEAB, second 50 µL 0.2% formic acid and third 50 µL 50% acetonitrile with 0.2% formic acid. The solution was subsequently frozen, desiccated in a vacuum concentrator and reconstituted in 0.2% formic acid.

The digestion of RPE cell lysates followed a similar protocol with some modifications. 15 µg total protein RPE sample (n=7 different differentiation experiments/sample) were adjusted to 23 µL and equal volumes of 2x concentrated S-trap lysis buffer was added (10% SDS in 100mM TEAB pH 8.5]. RPE samples were prepared using the same general steps of reduction, alkylation, and acidification. Digestion with trypsin was carried out for 1.5 hours at 47°C and peptides were eluted as outlined above.

Data Acquisition and processing

LCMS analysis for RO and RPE cells was conducted on an Exploris 480 Quadrupole-Orbitrap Mass Spectrometer (Thermo Fisher Scientific) and a Orbitrap Fusion™ Lumos™ Tribrid™ Mass Spectrometer (Thermo Fisher Scientific), respectively. For RO LCMS, equivalents of 1 µg of each peptide were loaded onto an Acclaim PepMap100 C18 LC Column (Thermo Fisher Scientific) and separated on a 75µm x25 cm C18 column (Thermo Fisher Scientific). For the loading of 1 µg RPE a 300µm x 5mm C18 PepMap trap cartridge (Thermo Fisher Scientific) was utilized and a 75µm x 50cm C18 column for separation (Thermo Fisher Scientific). The data was queried against the protein sequence database available on https://www.uniprot.org/uniprot/?query=proteome:UP000005640 using MaxQuant v2.0.3.0 (ROs), and Spectronaut v16.1 (Biognosys) (RPE) based on default settings such as cysteine alkylation: iodoacetamide, digestion enzyme: trypsin, and variable modifications: Oxidation (M), Acetyl (Protein N-term). Statistically significant changes in relative protein abundance were identified using modified

ANOVA (FDR<0.05, S0=0.1) followed by Tuckey's post hoc test (FDR<0.05). Metascape annotation of common pathways has been performed using https://metascape.org/gp/index.html#/main/step1 (Zhou et al., 2019).

Lipidomics

Samples were extracted in chloroform/methanol (2/1, v/v) and centrifuged at 1700 x *g* for 10 minutes. Phase partitioning was facilitated by the addition of 0.1 M KCL, whereby the lower organic phase containing the lipid fraction was retained. The lipid-enriched phase was subsequently dried in the presence of nitrogen gas prior to being reconstituted in methanol containing 5 mM ammonium formate. Lipidomic analysis was carried out under positive and negative ion modes over the mass to charge (*m/z*) range 250-2000 at a resolution of 100,000 using a Thermo Exactive Orbitrap mass spectrometer equipped with a heated electrospray ionization (HESI II) probe coupled to a Thermo Fisher Scientific ultimate 3000 RSLC system. Separation of lipids by column chromatography was performed using a Thermo Hypersil Gold C18 column (1.9µm; 2.1 mm × 100 mm) maintained at 50°C. Mobile phase A comprised an aqueous solution containing 10 mM ammonium formate and 0.1% (v/v) formic acid. Mobile phase B was made up of 90:10 isopropanol/acetonitrile (ACN) containing 10 mM ammonium formate and 0.1% (v/v) formic acid. The initial gradient conditions were 65%A/35%B. An increase the gradient of phase B was applied from 35% to 65% over 4 minutes, followed by 65%-100% over 15 minutes, with a hold for 2 minutes before re-equilibration to the starting conditions over 6 minutes. The gradient flow occurred at a rate of 400 µL/minute.

Transmission electron microscopy (TEM)

Samples were initially fixed in 2% glutaraldehyde 0.1 M sodium cacodylate and further subjected to an additional fixation by 2% osmium tetroxide. Samples were then dehydrated by a gradual increase. in acetone concentration, up to 100% then impregnated with increasing concentrations of epoxy resin. After 2x 100% resin steps, the samples were embedded in epoxy resin and polymerised at 60 degrees for 24 hours. Epoxy resin blocks were ultra-sectioned into slices of 70 nm thickness, collected on copper grids and further stained by uranyl acetate and lead citrate. TEM images were captured using a Hitachi HT7800 TEM microscope. TEM analyses entailed the blindfold selection of images to enable an unbiased evaluation of cellular morphology findings. Image segmentation and processing was conducted using the open-source Microscopy Image Browser software (Belevich et al., 2016).

References:

Belevich, I., Joensuu, M., Kumar, D., Vihinen, H. and Jokitalo, E. (2016) Microscopy Image Browser: A Platform for Segmentation and Analysis of Multidimensional Datasets. PLoS Biol, *14*, e1002340. https://doi.org/10.1371/journal.pbio.1002340.

Buskin, A. et al. (2018) Disrupted alternative splicing for genes implicated in splicing and ciliogenesis causes PRPF31 retinitis pigmentosa. Nat Commun. *9*, 4234. https://doi.org/ doi: 10.1038/s41467-018-06448-y.

Dorgau, B. et al. (2019) Decellularised extracellular matrix-derived peptides from neural retina and retinal pigment epithelium enhance the expression of synaptic markers and light responsiveness of human pluripotent stem cell derived retinal organoids. Biomaterials. *199*, 63-75. https://doi.org/10.1016/j.biomaterials.2019.01.028.

Kurzawa-Akanbi, M. et al. (2021) Neuropathological and biochemical investigation of Hereditary Ferritinopathy cases with ferritin light chain mutation: Prominent protein aggregation in the absence of major mitochondrial or oxidative stress. Neuropathology and Applied Neurobiology *47*, 26–42. https://doi.org/10.1111/nan.12634.

Kurzawa-Akanbi, M. et al. (2022) Retinal pigment epithelium extracellular vesicles are potent inducers of age-related macular degeneration disease phenotype in the outer retina. J Extracell Vesicles, *11*, 12295. https://doi.org/ 10.1002/jev2.12295.

Zhou, Y., Zhou, B., Pache, L., Chang, M., Khodabakhshi, A.H., Tanaseichuk, O., Benner, C. and Chanda, S.K. (2019) Metascape provides a biologist-oriented resource for the analysis of systems-level datasets. Nat Commun. *10*, 1523. https://doi.org/10.1038/s41467-019-09234-6.

Supplementary Figures

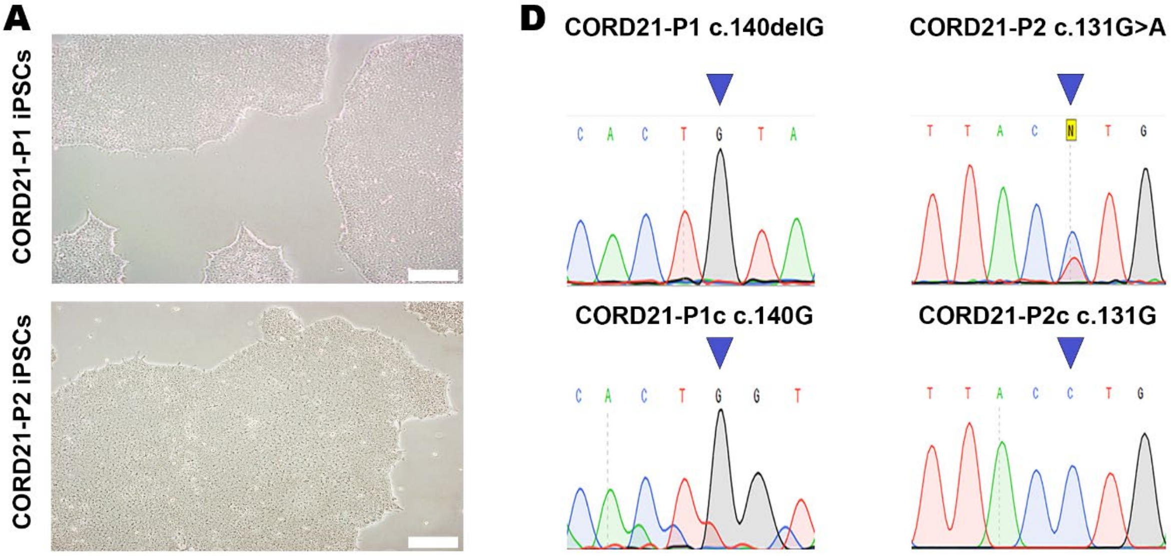

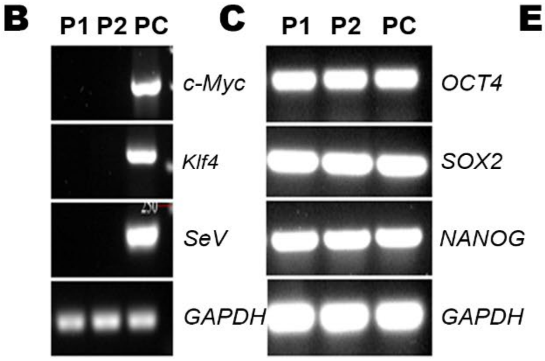

E

Sample ID	PluriTest Result	PluriCor	NovelCor
WT1	Pass	38.7929	1.496916
WT3	Pass	39.40208	1.307016
CORD21-P1	Pass	40.79827	1.480112
CORD21-P1c	Pass	39.06581	1.405857
CORD21-P2	Pass	40.26001	1.246298
CORD21-P2c	Pass	33.75519	1.367267
iPSC	Pass	46.91152	1.457459
Non_iPSC	Fail	-47.83872	2.737252

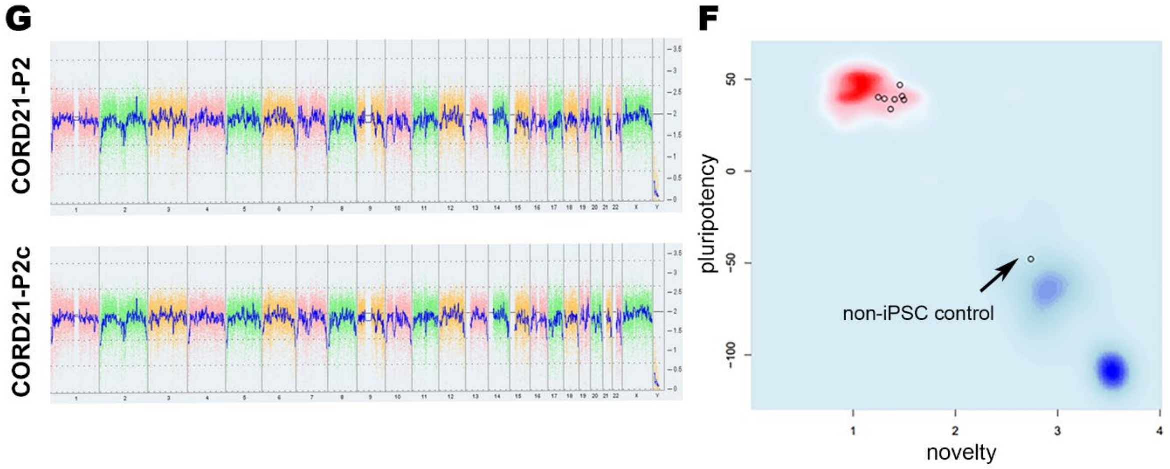

Figure S1. CORD21-iPSCs and the respective isogenic controls are pluripotent and lack genomic instabilities. (**A**) Brightfield images from the CORD21-P1 and -P2 iPSCs demonstrate a typical pluripotent stem cell colony-like morphology, scale bars=200 µm. (**B**) RT-PCR shows clearance of Sendai virus-associated transgenes in CORD21-P1 and -P2 iPSCs relative to positive control (PC) following iPSC reprogramming. (**C**) RT-PCR demonstrates expression of pluripotent marker genes (*OCT4*, *SOX2* and *NANOG)* relative to PC sample. *GAPDH* was used as a control. (**D**) DNA chromatograms show the absence of a guanine base at c.140 in CORD21-P1 and its respective presence in CORD21-P1c upon CRISPR-Cas9 correction. The heterozygous c.131G>A mutation present in CORD21-P2 is restored to the wild-type state in the CORD21-P2c isogenic control. (**E**) PluritestTM Table shows high pluripotency scores (PluriCor) for iPSC lines used in this study. (**F**) The pluripotency test plot is a visual representation of the samples based on pluripotency (y-axis) and the novelty score (x-axis) listed in the PluriTable (E). The x/y scatter plot shows the relative distribution of pluripotent (red) and non-pluripotent samples (blue) in the reference dataset. A non-iPSC sample indicated by arrow served as a negative control. (**G**) Whole genome view shows no chromosomal copy number abnormalities for somatic and sex chromosomes in CORD21-P2 and -P2c iPSCs. The smooth signal plot (y-axis) represents log2 ratios of microarray signal intensities, whereby a deviation from the normal chromosome copy number (CN=2) would indicate chromosomal copy number aberrations. Colours pertain to the raw chromosomal signal, whereby blue represents the normalized copy number signal.

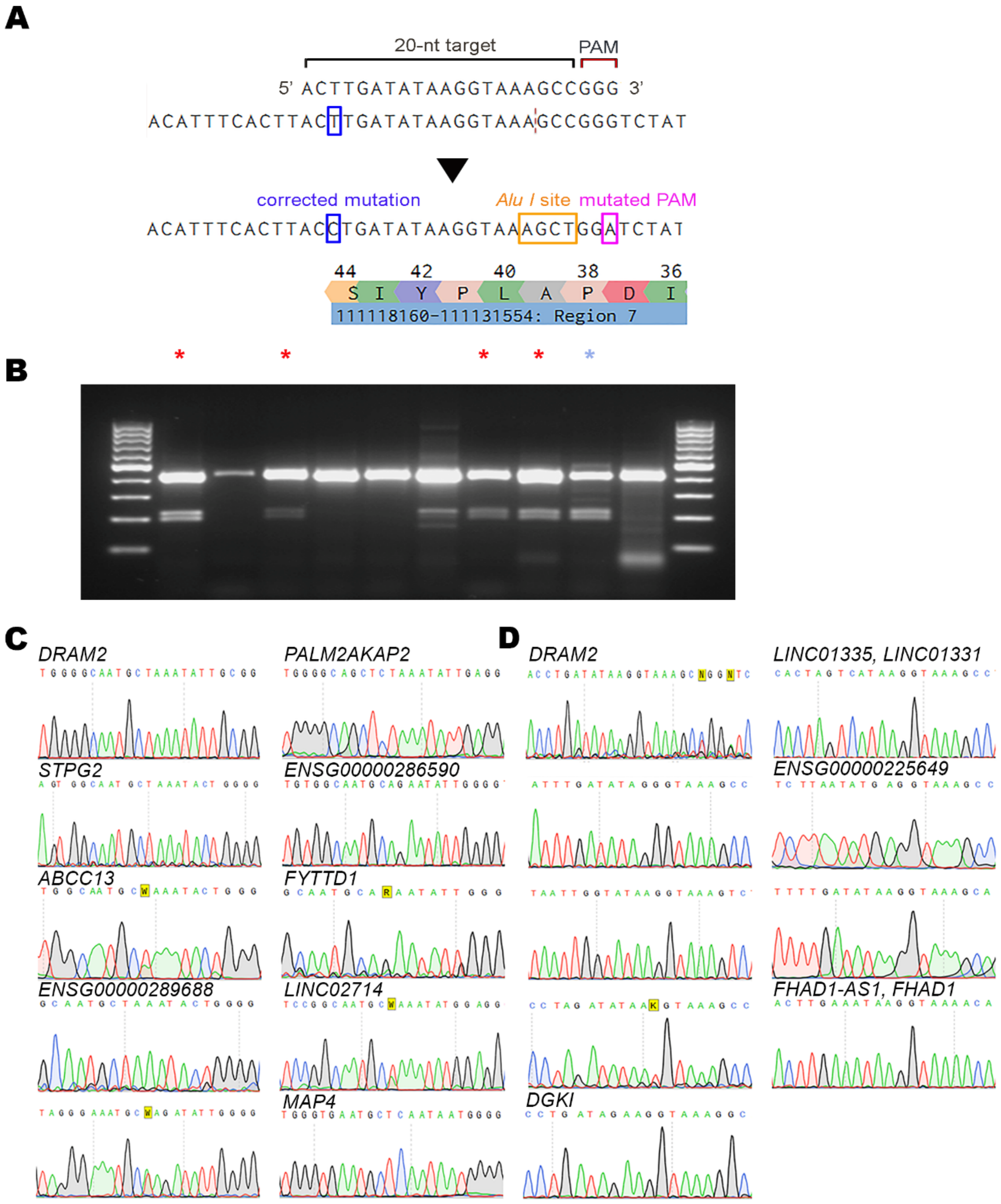

Figure S2. CRISPR-Cas9 *in situ* gene editing targets the *DRAM2* gene without off site effects in CORD21-P1c and -P2c iPSCs. (**A**) Sequence in the top panel shows gRNA used in CRISPR-Cas9 experiment (mutation site outlined by blue rectangle). Bottom panel shows genomic sequence following putative CRISPR-Cas9 correction of the c.131G>A mutation in CORD21-P2 iPSCs (corrected mutation outlined in blue, the *Alu I* and abrogated PAM sites marked in orange and magenta, respectively). (**B**) Representative gel image of CRISPR-edited clones following DNA extraction. iPSC clones marked by blue and red asterisks were selected as primary targets for Sanger sequencing due to producing expected band patterns of approximately 216 and 192 bps following *Alu I* restriction

digest. The clone marked by the blue asterisk corresponds to clone 72 which was used to generate the CORD21-P2c isogenic iPSC line. DNA chromatograms from Sanger sequencing confirm the absence of genomic alterations at sites homologous to the targeted genomic sequence as a result of the CRISPR-Cas9 editing of CORD21-P1c (n=10) (**C**) and CORD21-P2c iPSCs (n=9) (**D**). The name of genes covering these sequences can be seen in the top left corner of each chromatogram. Sites of homology were identified using Cas-OFFinder by allowing a maximum of three-mismatches in the targeted sequence (**Table S1**).

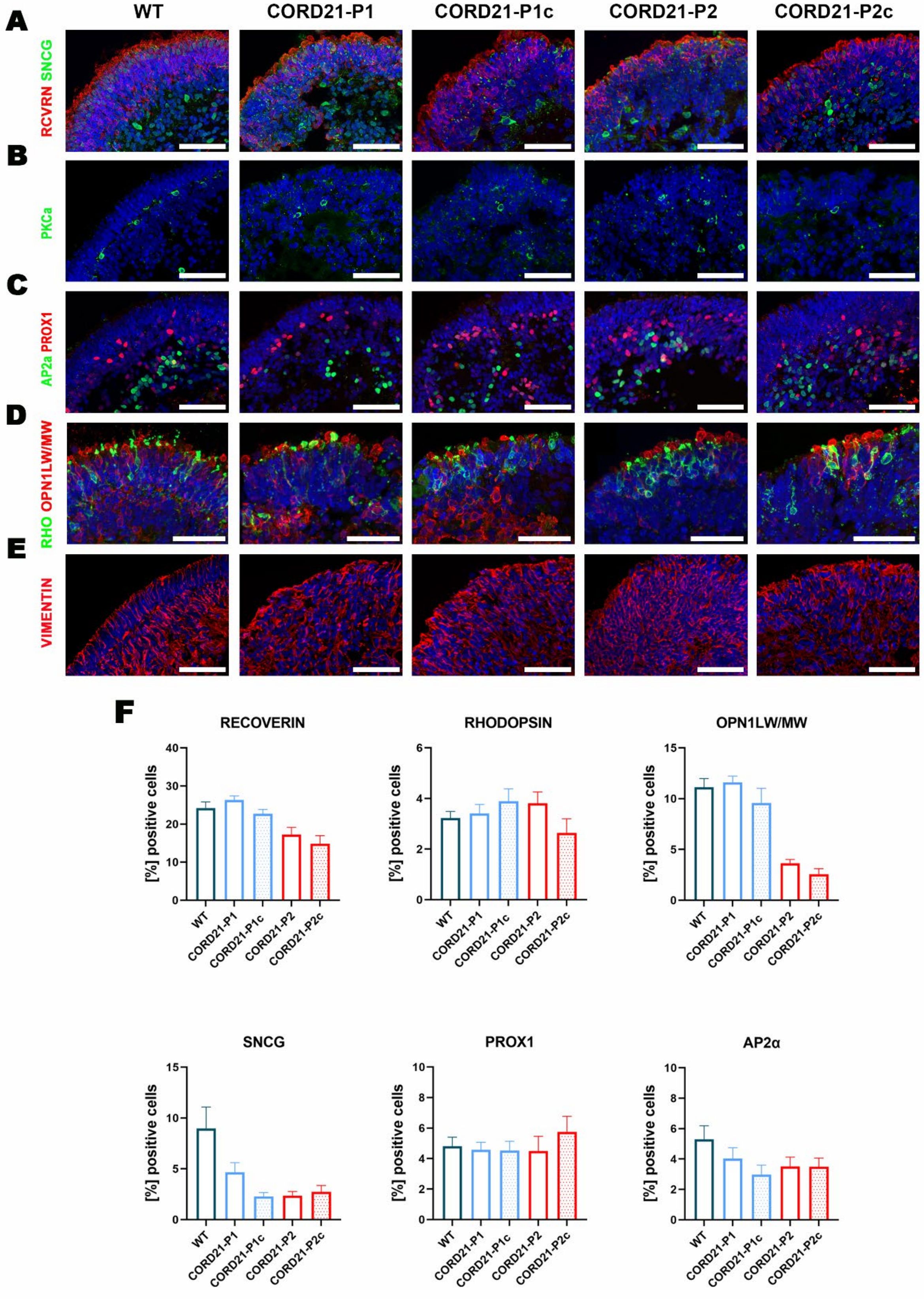
A
B
C
D
E
F
WT
CORD21-P1
CORD21-P1c
CORD21-P2
CORD21-P2c
RCVRN SNCG
PKCa
AP2a PROX1
RHO OPN1LW/MW
VIMENTIN
RECOVERIN
RHODOPSIN
OPN1LW/MW
SNCG
PROX1
AP2α
[%] positive cells

Figure S3. Characterisation of day 220 CORD21- and control-ROs by immunofluorescence (IF) analyses. IF experiment demonstrated the presence of all retinal cell types such as photoreceptor (**A**) (Recoverin, red) and ganglion cells (SNCG, green), (**B**) rod bipolar cells (PKCα, green), (**C**) horizontal (PROX1, red) and amacrine (AP2α, green) cells, (**D**) red/green cones (OPN1LW/MW, red) and rod photoreceptors (RHO, green) as well as (**E**) Müller glia (vimentin, red). Nuclei are counterstained with Hoechst (blue). These are representative examples from 15 ROs imaged from three different differentiation experiments/sample. Scale bars = 50µm for RCVRN/SNCG, PKCα, PROX1/AP2α and vimentin, and 20µm for RHO/OPN1LW/MW. (**F**) Quantification bar plots show % positive cells corresponding to the expression of each marker relative to the total number of cells. Data presented as mean + SEM (n=15 ROs imaged from three different differentiation experiments/sample).

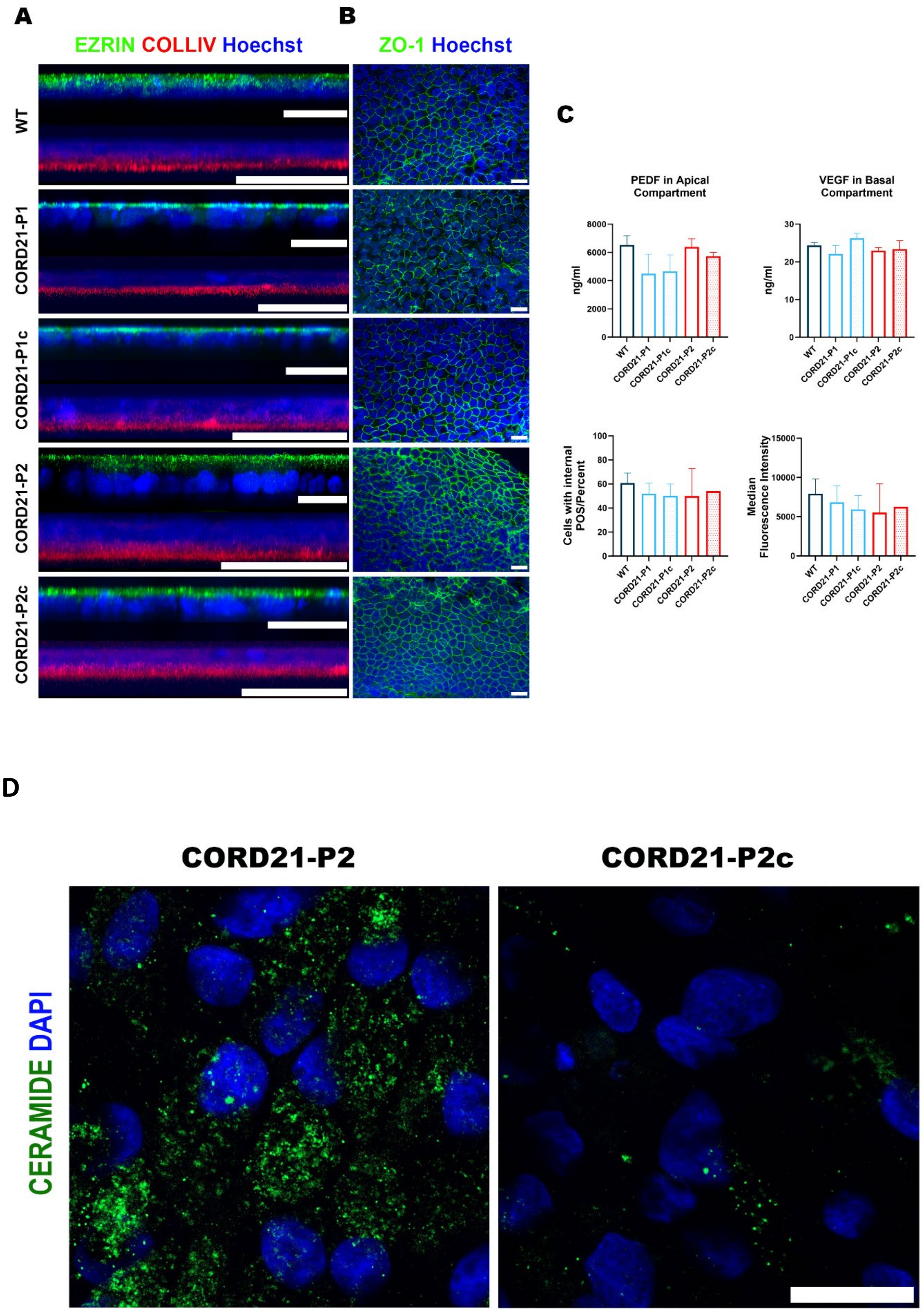
A
EZRIN COLLIV Hoechst
WT
CORD21-P1
CORD21-P1c
CORD21-P2
CORD21-P2c
B
ZO-1 Hoechst
C
PEDF in Apical Compartment
ng/ml
VEGF in Basal Compartment
Cells with internal POS/Percent
Median Fluorescence Intensity
WT
CORD21-P1
CORD21-P1c
CORD21-P2
CORD21-P2c
D
CORD21-P2
CORD21-P2c
CERAMIDE DAPI

Figure S4. RPE generation and characterisation by IF and ELISA assays. (**A**) CORD21- and control RPE cells display the apical expression of EZRIN and basal expression of Collagen IV (COL IV), scale bar=20μm. These are representative examples from 15 RPE transwells imaged from three different differentiation experiments/sample. (**B**) CORD21- and control RPE cells display the expression of the tight junction marker ZO-1, scale bar=20μm. (A, B) These are representative examples from 15 RPE transwells imaged from three different differentiation experiments/sample. (**C**) No significant differences in apical section of PEDF, basal secretion of VEGF and the ability to phagocytose photoreceptor outer segments (POSs) are observed between the CORD21- and control RPE cells. Data presented as mean + SEM (n=9 RPE transwells from three different differentiation experiments/sample). **(D)** Ceramide accumulation in CORD21-RPE cells. These are representative examples from 15 RPE transwells imaged from three different differentiation experiments. Scale bar=20μm.

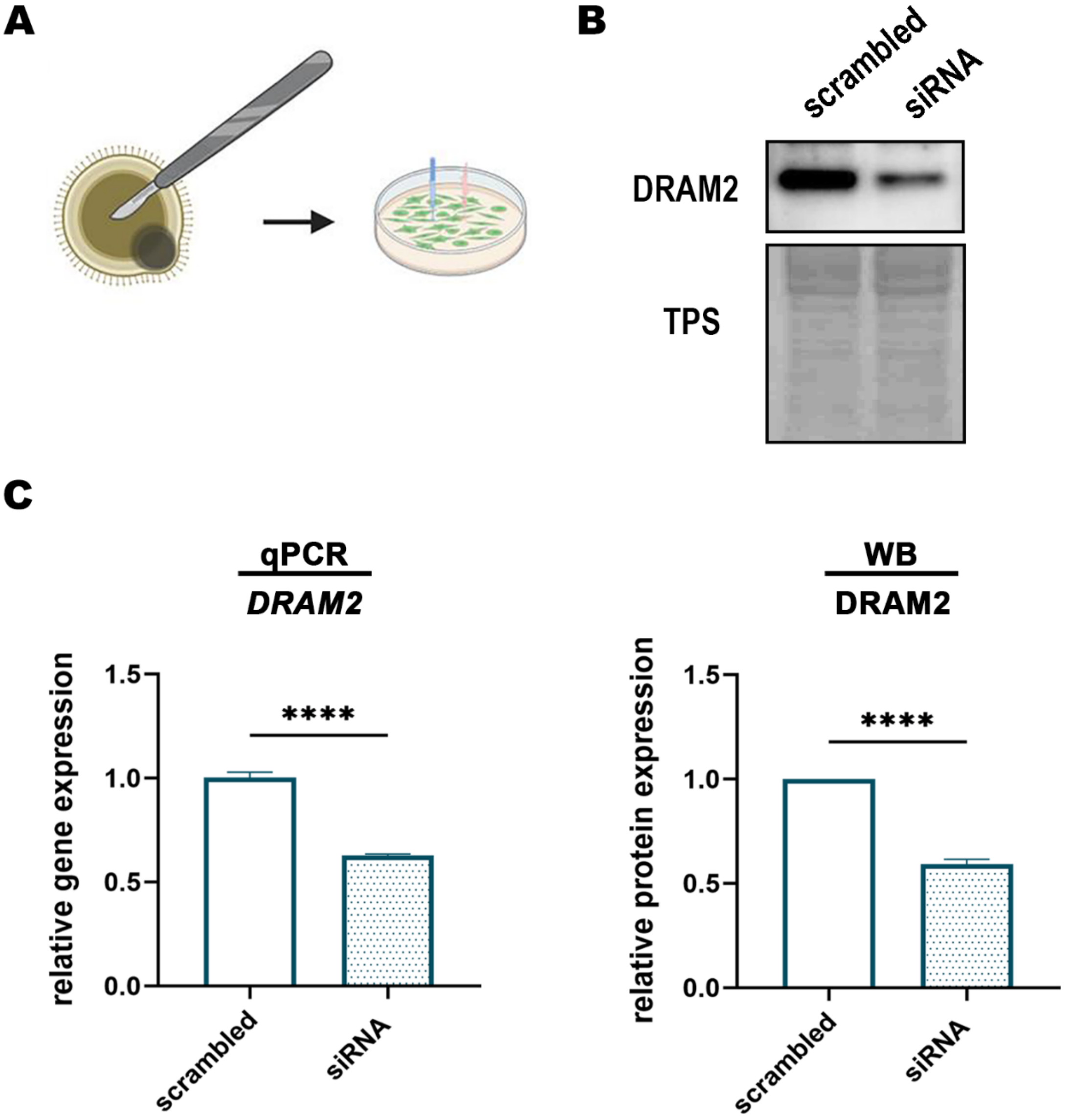

Figure S5. DRAM2 antibody validation and protein detection in ROs by western blot. (**A**) WT day 230 ROs were dissected and expanded on Matrigel™, and poly-L-ornithine coated 24-well plates. Treatment with 20µM *DRAM2* and scrambled siRNA was carried out for a period of 72 hours. (**B**) DRAM2 protein detection (~26kDa) in day 220 patient and control ROs. TPS stands for total protein stain showing equal sample loading. Quantitative analysis (bottom panel) confirms 50% reduction in DRAM2 protein expression. (**C**) Quantitative RT-PCR confirms *DRAM2* knockdown in siRNA treated ROs compared to scrambled control. (B-C) Data are presented as mean + SEM (n=3 different differentiation experiments each consisting of 24 ROs/sample). **** $p<0.0001$.

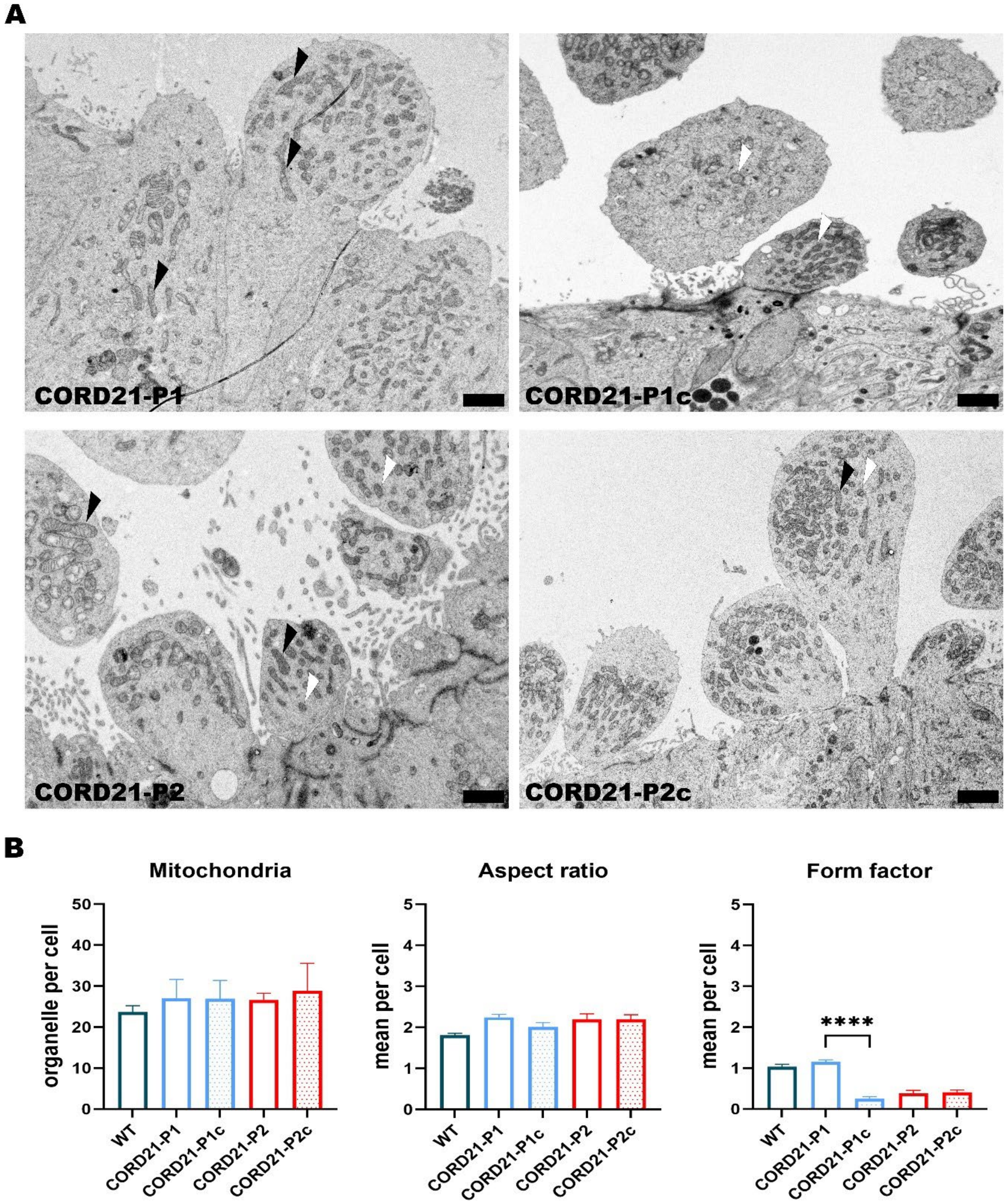

Figure S6. CORD21-P1-ROs show increased number of branched mitochondria relative to isogenic control. (**A**) CORD21-P1 image indicates the higher abundance of more branched mitochondria in photoreceptor cell bodies, and ISs as opposed to CORD21-P1c where less mitochondrial branching is apparent. Images from CORD21-P2 and CORD21-P2c demonstrate high levels of mitochondrial diversity, whereby both smaller and more elongated mitochondria are apparent in the photoreceptor cell bodies/inner segment. Elongated mitochondria are shown by black arrow heads and rounded mitochondria by white arrow heads, respectively. (**B**) Bar plots show no difference in mitochondrial

count per cell and aspect ratio. Significant differences are observed for form factor which indicates changed mitochondrial branching between CORD21-P1 and CORD21-P1c photoreceptors. Data are presented as mean + SEM. Mitochondrial segmentation analysis was carried out using the Microscopy Image Browser software (n=10 ROs from three different differentiation experiments/sample). Statistical difference for CORD21-P1vs -P1c is denoted by ****$p<0.0001$.

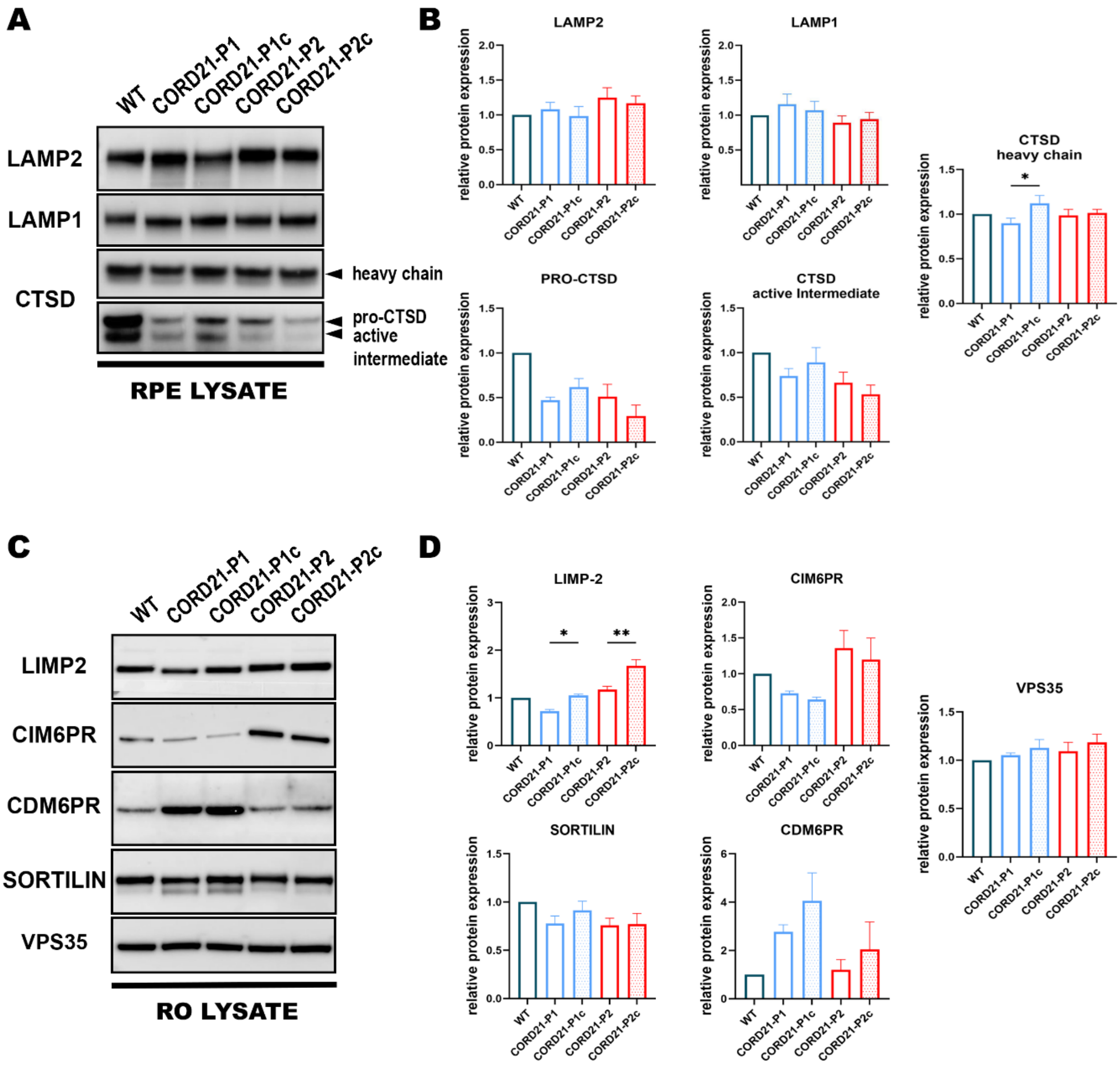

Figure S7. Additional screening of transport proteins suggests cellular transport dysfunction as indicated by the depletion of specific lysosomal receptors. (**A-B**) RPE cells show no differences in LAMP2 and LAMP1 expression and corroborate the downregulation of CTSD heavy chain in the lysates of CORD21--P1-RPE cells. Furthermore, no changes were observed for CTSD active intermediate. Data are presented as mean + SEM (n=3 different differentiation experiments each consisting of 2 wells of a 12-well plate of RPE cells/sample) and normalised to the WT sample. (**C-D**) Western blot analyses in day 220 RO lysates reveals a consistent downregulation in expression of GBA receptor LIMP2 in both CORD21-ROs. Conversely, no significant differences were established for the expression of CIM6PR, CDM6PR, VPS35 and sortilin. Data are presented as mean + SEM (n=3-4 differentiation experiments each consisting of 48 ROs/sample) and normalised to the WT sample. Equal protein loading was visualised by the total protein stain. Statistical comparisons for CORD21-P1vs -P1c and CORD21-P2vs-P2c are denoted by * p<.05, **p<.01.

Supplementary Table Legends

Table S1. A summary of CORD21 patient clinical information and the reagents used for CRISPR/Cas9 screening, RT-PCR, western blot and immunofluorescence analyses.

Table S2. A summary of proteomics analyses conducted in CORD21-ROs and RPE cells and the heterozygous isogenic controls.
